# Personalized mechanical ventilation guided by ultrasound in patients with acute respiratory distress syndrome (PEGASUS): study protocol for an international randomized clinical trial

**DOI:** 10.1186/s13063-024-08140-7

**Published:** 2024-05-07

**Authors:** Jante S. Sinnige, Marry R. Smit, Aniruddha Ghose, Harm-Jan de Grooth, Theis Skovsgaard Itenov, Eleni Ischaki, John Laffey, Frederique Paulus, Pedro Póvoa, Charalampos Pierrakos, Luigi Pisani, Oriol Roca, Marcus J. Schultz, Konstanty Szuldrzynski, Pieter R. Tuinman, Claudio Zimatore, Lieuwe D. J. Bos, for the PEGASUS investigators, A. K. M. Arif Uddin Ahmed, Mohammad Jhahidul Alam, Mohsammad Rafiqual Alam, Anjan Bal, Samarjit Barua, Rajdeep Biswas, Mohammed Abdur Rahaman Chowdhury, Safiqul Mostafa Chy, Satyajit Dhar, Pranay Kumar Dutta, Syeda Nafisa Khatoon, Ranjan Kumar Nath, Nahid Nowroz, Mithun Shil, Rachid Attou, Leonel Barreto Gutierrez, Keitiane Kaefer, Morten Bestle, Lars Hein, Thomas Hildebrandt, Jacob Jensen, Sanne Lauritzen, Ulf Pedersen, Lone Poulsen, Harry Giannopoulos, Katerina Vaporidi, Lauren Ferguson, Yvelynne Kelly, Sabina Mason, Aisling McMahon, Bairbre McNicholas, Daniele Biasucci, Gianmaria Cammarota, Maurizio Follino, Salvatore Grasso, Antonio Latela, Giovanna Magnesa, Fabrizia Massaro, Leonarda Maurmo, Marco Pezzuto, Savino Spadaro, Luigi Vetrugno, Massimo Zambon, Daan Filippini, Peter Klompmaker, Amne Mousa, Dominik Daszuta, Miłosz Jankowski, Irene Aragao, Heloisa Castro, Vasco Costa, Cristina Torrão, Toni Antoni, Marta Arroyo, Marta Briva, Nuria Duran, Marina García-de-Acilu, Gemma Goma, Ana Ochagavia, Michelle Chew, Mariangela Pellegrini, Gaetano Perchiazzi

**Affiliations:** 1 Department of Intensive Care, Amsterdam University Medical Centres (UMC), University of Amsterdam, Meibergdreef 9, Amsterdam, AZ 1105 The Netherlands; 2 Department of Medicine, Chattogram Medical Centre, Chattogram, Bangladesh; 3 Department of Intensive Care, UMC, Vrije Universiteit, Amsterdam, HV 1081 The Netherlands; 4 Department of Anesthesiology and Intensive Care, Copenhagen University Hospital - Bispebjerg and Frederiksberg, Copenhagen, Denmark; 5 Department of Clinical Medicine, University of Copenhagen, Copenhagen, Denmark; 6 First Department of Intensive Care Medicine, University of Athens Medical School, 10676 Athens, AZ Greece; 7 Anaesthesia and Intensive Care Medicine, School of Medicine, Galway University Hospitals, University of Galway, Galway, H91 TK33 Ireland; 8 NOVA Medical School, CHRC, NOVA University of Lisbon, Lisbon, Portugal; 9 Center for Clinical Epidemiology and Research Unit of Clinical Epidemiology, OUH Odense University Hospital, Odense, Denmark; 10 Department of Intensive Care, Hospital de São Francisco Xavier, CHLO, Lisbon, Portugal; 11 Department of Intensive Care, Brugmann University Hospital, Université Libre de Bruxelles, 1050 Brussels, Belgium; 12 Department of Precision-Regenerative Medicine and Jonic Area (DiMePRe-J), Section of Anesthesiology and Intensive Care Medicine, University of Bari Aldo Moro, Bari, Italy; 13 Servei de Medicina Intensiva, Parc Taulí Hospital Universitari, Institut de Recerca Part Taulí (I3PT-CERCA), Parc del Taulí 1, 08028 Sabadell, Spain; 14 Departament de Medicina, Universitat Autònoma de Barcelona, Bellaterra, Spain; 15 Mahidol Oxford Tropical Medicine Research Unit (MORU), Mahidol University, Bangkok, 10400 Thailand; 16 Nuffield Department of Medicine, University of Oxford, Oxford, OX3 7BN UK; 17 Department of Anaesthesiology and Intensive Care, National Institute of Medicine of the Ministry of Interior and Administration, 02-507 Warsaw, Poland; 18 Amsterdam Cardiovascular Sciences, Amsterdam UMC, Vrije Universiteit Amsterdam, De Boelelaan 1117, Amsterdam, Netherlands; 19 Department of Emergency and Organ Transplantation, University of Bari Aldo Moro, 70124 Bari, Italy; 20 Department of Pulmonology, Amsterdam UMC, University of Amsterdam, Amsterdam, AZ 1105 The Netherlands; 21 Laboratory of Experimental Intensive Care and Anaesthesiology (L.E.I.C.A.), University of Amsterdam, Amsterdam, AZ 1105 The Netherlands

**Keywords:** Acute respiratory distress syndrome, Personalized medicine, Mechanical ventilation, Lung ultrasound

## Abstract

**Background:**

Acute respiratory distress syndrome (ARDS) is a frequent cause of hypoxemic respiratory failure with a mortality rate of approximately 30%. Identifying ARDS subphenotypes based on “focal” or “non-focal” lung morphology has the potential to better target mechanical ventilation strategies of individual patients. However, classifying morphology through chest radiography or computed tomography is either inaccurate or impractical. Lung ultrasound (LUS) is a non-invasive bedside tool that can accurately distinguish “focal” from “non-focal” lung morphology. We hypothesize that LUS-guided personalized mechanical ventilation in ARDS patients leads to a reduction in 90-day mortality compared to conventional mechanical ventilation.

**Methods:**

The Personalized Mechanical Ventilation Guided by UltraSound in Patients with Acute Respiratory Distress Syndrome (PEGASUS) study is an investigator-initiated, international, randomized clinical trial (RCT) that plans to enroll 538 invasively ventilated adult intensive care unit (ICU) patients with moderate to severe ARDS. Eligible patients will receive a LUS exam to classify lung morphology as “focal” or “non-focal”. Thereafter, patients will be randomized within 12 h after ARDS diagnosis to receive standard care or personalized ventilation where the ventilation strategy is adjusted to the morphology subphenotype, i.e., higher positive end-expiratory pressure (PEEP) and recruitment maneuvers for “non-focal” ARDS and lower PEEP and prone positioning for “focal” ARDS. The primary endpoint is all-cause mortality at day 90. Secondary outcomes are mortality at day 28, ventilator-free days at day 28, ICU length of stay, ICU mortality, hospital length of stay, hospital mortality, and number of complications (ventilator-associated pneumonia, pneumothorax, and need for rescue therapy). After a pilot phase of 80 patients, the correct interpretation of LUS images and correct application of the intervention within the safe limits of mechanical ventilation will be evaluated.

**Discussion:**

PEGASUS is the first RCT that compares LUS-guided personalized mechanical ventilation with conventional ventilation in invasively ventilated patients with moderate and severe ARDS. If this study demonstrates that personalized ventilation guided by LUS can improve the outcomes of ARDS patients, it has the potential to shift the existing one-size-fits-all ventilation strategy towards a more individualized approach.

**Trial registration:**

The PEGASUS trial was registered before the inclusion of the first patient, https://clinicaltrials.gov/ (ID: NCT05492344).

**Supplementary Information:**

The online version contains supplementary material available at 10.1186/s13063-024-08140-7.

## Administrative information

Note: the numbers in curly brackets in this protocol refer to SPIRIT checklist item numbers. The order of the items has been modified to group similar items (see http://www.equator-network.org/reporting-guidelines/spirit-2013-statement-defining-standard-protocol-items-for-clinical-trials/).

**Table:** 

Title {1}	Personalized Mechanical Ventilation Guided by UltraSound in Patients with Acute Respiratory Distress Syndrome (PEGASUS): study protocol for an international randomized clinical trial.
Trial registration {2a and 2b}.	https://clinicaltrials.gov, ID: NCT05492344
Protocol version {3}	Protocol version 4.0, date 02–11-2022
Funding {4}	Funding from the Amsterdam UMC.
Author details {5a}	^*^ Correspondence: Jante S. Sinnige, j.s.sinnige@amsterdamumc.nl. ^1^ Department of Intensive Care, Amsterdam University Medical Centres (UMC), University of Amsterdam, 1105 AZ Amsterdam, The Netherlands. ^2^ Department of Medicine, Chattogram Medical Centre, Chattogram, Bangladesh. ^3^ Department of Intensive Care, Amsterdam UMC, Vrije Universiteit, 1081 HV Amsterdam, The Netherlands. ^4^ Department of anesthesiology and intensive care, Copenhagen University Hospital—Bispebjerg and Frederiksberg, Copenhagen, Denmark. ^5^ Department of Clinical Medicine, University of Copenhagen, Copenhagen, Denmark. ^6^ First Department of Intensive Care Medicine, University of Athens Medical School, 10,676 AZ Athens, Greece. ^7^ Anaesthesia and Intensive Care Medicine, Galway University Hospitals and School of Medicine, University of Galway, H91 TK33, Ireland. ^8^ NOVA Medical School, CHRC, NOVA University of Lisbon, Portugal. ^9^ Center for Clinical Epidemiology and Research Unit of Clinical Epidemiology, OUH Odense University Hospital, Denmark. ^10^ Department of Intensive Care, Hospital de São Francisco Xavier, CHLO, Lisbon, Portugal. ^11^ Department of Intensive Care, Brugmann University Hospital, Université Libre de Bruxelles,1050 Brussels, Belgium. ^12^ Department of Precision-Regenerative Medicine and Jonic Area (DiMePRe-J), Section of Anesthesiology and Intensive Care Medicine, University of Bari "Aldo Moro", Bari, Italy. ^13^ Servei de Medicina Intensiva, Parc Taulí Hospital Universitari, Institut de Recerca Part Taulí (I3PT-CERCA), Parc del Taulí 1, 08028 Sabadell, Spain. ^14^ Departament de Medicina, Universitat Autònoma de Barcelona, Bellaterra, Spain. ^15^ Mahidol Oxford Tropical Medicine Research Unit (MORU), Mahidol University, Bangkok 10,400, Thailand. ^16^ Nuffield Department of Medicine, University of Oxford, Oxford OX3 7BN, UK. ^17^ Department of Anaesthesiology and Intensive Care, National Institute of Medicine of the Ministry of Interior and Administration, 02–507 Warsaw, Poland. ^18^ Amsterdam Cardiovascular Sciences, Amsterdam UMC, Vrije Universiteit Amsterdam, De Boelelaan 1117, Amsterdam, Netherlands ^19^ Department of Emergency and Organ Transplantation, University of Bari Aldo Moro, 70,124 Bari, Italy. ^20^ Department of Pulmonology, Amsterdam UMC, University of Amsterdam, 1105 AZ Amsterdam, The Netherlands. ^21^ Laboratory of Experimental Intensive Care and Anaesthesiology (L.E.I.C.A.), University of Amsterdam, 1105 AZ Amsterdam, The Netherlands.
Name and contact information for the trial sponsor {5b}	Dr. Lieuwe D. J. Bos Department of Intensive Care Amsterdam University Medical Centers Meibergdreef 9 1105 AZ Amsterdam The Netherlands Email address: l.d.bos@amsterdamumc.nl
Role of sponsor {5c}	The sponsor is responsible for the study design, overseeing data collection, management of sites, analysis and interpretation of data, writing of the report, and the decision to submit the report for publication.

## Introduction

### Background and rationale {6a}

Patients with acute respiratory distress syndrome (ARDS) present with acute hypoxemic respiratory failure due to exudative pulmonary edema [1]. ARDS develops in about 25% of patients undergoing mechanical ventilation in the intensive care unit (ICU) and is associated with a mortality of 30–40% as well as high morbidity in survivors [2, 3]. Respiratory support is aimed at limiting ventilator-induced lung injury through low tidal volume ventilation in all patients, and prone positioning in patients with low PaO_2_/FiO_2_ ratio despite optimization of ventilator settings. Aside from ARMA, numerous trials attempting to establish the optimal management of ARDS have been conducted in unselected ARDS patient populations but have failed to improve outcomes [4], likely related to considerable heterogeneity within the ARDS population [5, 6].

Personalized medicine could potentially address the considerable heterogeneity within ARDS by tailoring treatment to individual patient characteristics [5]. Based on radiological appearance, ARDS patients can be divided into a “non-focal” or a “focal” subphenotype, with “non-focal” ARDS manifesting as a diffuse and patchy loss of aeration distributed diffusely in the lungs and “focal” ARDS having predominantly dorsal-inferior consolidations [7]. “Non-focal” ARDS patients tend to respond better to higher levels of positive end-expiratory pressure (PEEP) and recruitment maneuvers while patients with “focal” ARDS respond better to lower levels of PEEP and prone positioning [8].

While computed tomography (CT) is considered the gold standard to define these morphology subphenotypes, it demands transportation of the critically ill patient, is not available in every hospital and requires interpretation by an experienced physician [8, 9]. Chest X-ray (CXR) is a more available bedside modality to define the morphology subphenotype; however, it has a higher risk of misclassification [10]. This problem had a significant impact on the LIVE study, a randomized controlled trial (RCT), where patients received personalized ventilation mostly based on CXR, and 20% of patients were misclassified because of poor interobserver agreement in the interpretation of chest images [8]. Even though there was no overall mortality benefit in all patients (correctly classified and misclassified), patients with correctly classified lung morphology did benefit from a personalized ventilation strategy with a 10% decrease in mortality, while patients who were misclassified had a substantial increase in mortality when exposed to a misaligned personalized ventilation strategy. Thus, accurate classification seems sensible before starting a personalized ventilation strategy based on lung morphology.

Lung ultrasound (LUS) is gaining popularity in the ICU setting because it can adequately assess lung aeration compared to CT and it is readily available at the bedside [11, 12]. Moreover, LUS is easy to learn and it offers a very high interobserver agreement [13, 14, 15]. Recently, our group developed a LUS method for the classification of lung morphology in ARDS patients [16]. The method was trained and validated using multicenter international datasets of simultaneously acquired LUS and CT exams. This LUS method could correctly distinguish “focal” from “non-focal” lung morphology with a sensitivity of 77%, a specificity of 100%, and an accuracy of 89% when compared to the gold standard chest CT, and thus could play an important role in guiding personalized ventilation in ARDS patients.

The PEGASUS study integrates the results of the LIVE study in correctly classified patients and the accurate diagnostic evaluation of lung morphology using ultrasound to assess the effect of personalized ventilation in ARDS patients. A pilot phase will be included to evaluate the correct classification of the LUS images, safety of the delivered intervention and to ensure the protocol adherence. We hypothesize that personalized mechanical ventilation based on lung morphology assessed by LUS leads to a reduced mortality compared to conventional mechanical ventilation in ARDS patients [17, 8, 16].

### Objectives {7}

The primary objective of this study is to determine if personalized mechanical ventilation based on lung morphology assessed by LUS reduces all-cause mortality at day 90 compared to conventional mechanical ventilation in ARDS patients. The secondary objectives of this study are to evaluate if personalized mechanical ventilation based on lung morphology assessed by LUS leads to a reduced mortality at day 28, more ventilator-free days (VFD) at day 28, a shorter ICU length of stay, lower ICU mortality, shorter hospital length of stay, lower hospital mortality, lower number of patients with complications (ventilator-associated pneumonia (VAP), pneumothorax), and less need for adjunctive (recruitment, prone position) and rescue therapies (extracorporeal membrane oxygenation (ECMO), inhaled vasodilators, airway pressure release ventilation, neuromuscular blockage, dialysis, tracheostomy). The objective of the pilot phase is to ensure feasibility of the study, accurate application and interpretation of the LUS algorithm, and delivery of personalized mechanical ventilation within the “safe limits”.

### Trial design {8}

This is an investigator-initiated, multicenter, international, superiority RCT (1:1). This study includes a pilot phase to evaluate the feasibility of the intervention arm with a personalized treatment strategy.

## Methods: participants, interventions and outcomes

### Study setting {9}

The study will run in ± 40 academic and non-academic centers in- and outside the European Union. A current list of participating centers can be found on https://clinicaltrials.gov/ (ID: NCT05492344).

### Eligibility criteria {10}

Patients will be included when they meet all of the following inclusion criteria: admitted to a participating ICU, invasively ventilated and fulfil the Berlin criteria for moderate or severe ARDS [6], and none of the following exclusion criteria are present: age under 18, participation in other interventional studies with conflicting endpoints, conditions in which LUS is not feasible or possible (e.g., subcutaneous emphysema, wounds), mechanical ventilation for longer than 7 consecutive days in the past 30 days, diagnosis of ARDS for longer than 12 h, history of ARDS in the previous month, body mass index higher than 40 kg/m^2^, intracranial hypertension, broncho-pleural fistula, chronic respiratory diseases requiring long-term oxygen therapy or respiratory support, pulmonary fibrosis with a vital capacity < 50% (severe or very severe), patients who are moribund or facing end of life, receiving or planned to receive ECMO, patients who receive invasive ventilation in home setting due to a neurological disease, previously randomized in this study, and if no informed or deferred consent could be obtained.

Hospitals with an intensive care unit are able to participate in the PEGASUS study when ethical approval is obtained and all the facilities needed for the PEGASUS study are present (ultrasound, arterial blood gas analysis) and staff are trained for the interventions (e.g., lung recruitment, defining morphology subphenotype, prone positioning).

### Who will take informed consent? {26a}

The local principal investigator, who is trained by a member of the steering committee, or her/his delegate, will obtain consent. Patients admitted for ventilator support to the ICU are, without exception, not able to give informed consent. In the participating countries, local regulations will determine when and how informed consent can be obtained from a proxy (legal representative) and when deferred informed consent can be used. The most liberal approach mandated by the study protocol is deferred proxy consent. In this situation, patients are randomized and informed consent from a legal representative will be obtained as soon as possible, but always within 72 h after randomization. If informed consent is not obtained within 72 h or if a legal representative declines participation, the patient will be excluded and data will not be used. When a patient dies before informed consent can be obtained from the legal representative, the data will be used and representative will be informed about the study. This approach has been approved by the ethical board in the Netherlands, Italy, Denmark, Belgium, Bangladesh, Greece, and Ireland. If the ethical board of a participating site does not approve deferred consent due to local regulations, informed consent before randomization by proxy will be accepted as an alternative. When possible, informed consent will also be obtained from the patient when the patient is recovered.

### Additional consent provisions for collection and use of participant data and biological specimens {26b}

N/a. The PEGASUS study does not collect extra personal data or biological specimens that fall outside of the scope of a standard informed consent procedure and therefore additional consent provisions are not required.

## Interventions

### Explanation for the choice of comparators {6b}

**Fig. 1:**
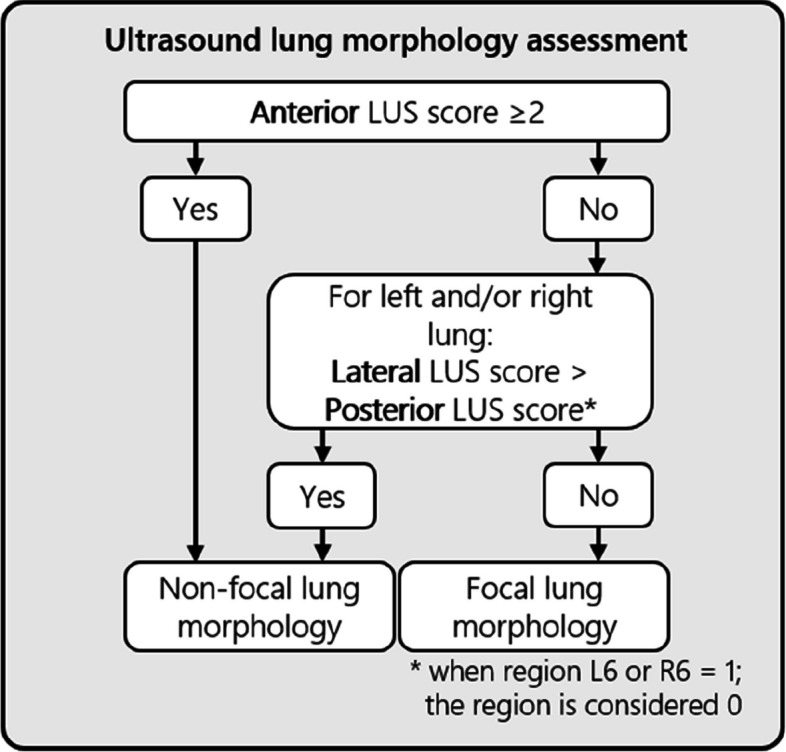
This logarithm is used to determine the lung morphology by ultrasound of eligible patients. The scores are LUS aeration scores explained in supplement 1 based on a 12-region LUS exam. *LUS* lung ultrasound

Patients who meet all the inclusion criteria and none of the exclusion criteria will receive a 12-region LUS exam (supplement 1) to determine lung morphology using the algorithm presented in Fig. 1. Patients will be randomly assigned within 12 h after ARDS diagnosis to the intervention group, with personalized mechanical ventilation, or to the standard care, where patients will receive standard care. The standard care group will be ventilated according to the current guidelines advised by the European Society of Intensive Care Medicine (ESICM) (Table 1). In these patients, the PEEP level will be selected according to the low PEEP/ high FiO_2_ table from the ALVEOLI study maintaining an end-inspiratory plateau pressure (Pplat) below 30 cmH_2_O (Table 2) [18]. Prone position is encouraged if PaO_2_/FiO_2_ ratio is ≤ 150 and recruitment maneuvers will be used as rescue therapy.

**Table 1:** Ventilation strategy for randomization and lung morphology groups

	**Standard of care**	**Personalized group**	
		“Focal”	“Non-focal”
**Mode of ventilation**	Pressure controlled, volume controlled or pressure support	Pressure controlled, volume controlled or pressure support	Pressure controlled, volume controlled or pressure support
**Tidal volume**	6 mL/kg PBW	6 to 8 mL/kg PBW	4 to 6 mL/kg PBW
**PEEP**	Table 2	≤ 9 cm H_2_O	≥ 15 cm H_2_O
**Recruitment maneuver**	Only for rescue	Only for rescue	Daily^a^
**Prone positioning**	PaO_2_/FiO_2_ < 150	PaO_2_/FiO_2_ < 200	PaO_2_/FiO_2_ < 150

Ventilation strategies according to our study protocol per randomization arm and lung morphology. Formula for calculating the tidal volume size with PBW are 50 + 0.91 × (centimeters of height − 152.4) for males and 45.5 + 0.91 × (centimeters of height − 152.4) for females [19]. ^a^A more detailed description of the ventilation strategies can be found in supplement 2, including the recruitment maneuver. *PBW* predicted body weight, *PEEP* positive end-expiratory pressure, *PaO*_*2*_ partial pressure of oxygen in arterial blood, *FiO*_*2*_ fraction of inspired oxygen

**Table 2:** FiO_2_ and PEEP strategy for the standard care group

**FiO**_**2**_	0.3	0.4	0.4	0.5	0.5	0.6	0.7	0.7	0.7	0.8	0.9	0.9	0.9	1.0
PEEP	5	5	8	8	10	10	10	12	14	14	14	16	18	18–24

During start or deterioration of patients included in the standard of care group, this table is used to determine the PEEP and FiO2 settings according to the low PEEP/ high FiO_2_ table of the ALVEOLI study. *FiO*_*2*_ fraction of inspired oxygen, *PEEP* positive end-expiratory pressure in cm H_2_O [18]

### Intervention description {11a}

**Fig. 2:**
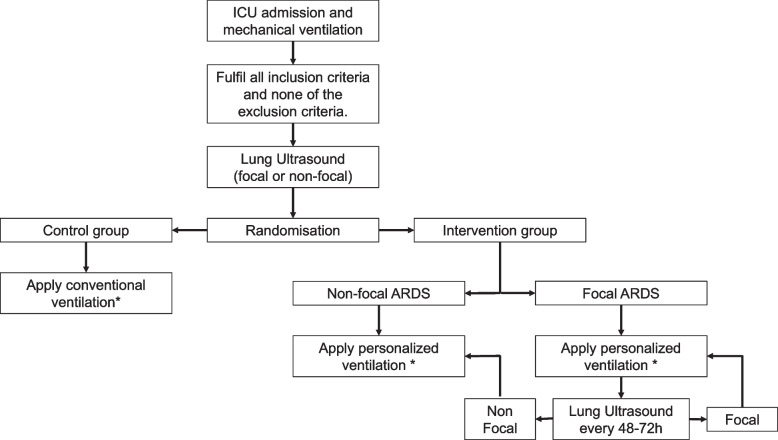
Flow diagram for enrollment of a PEGASUS participant. Patients with “focal” ARDS randomized in the intervention group will receive follow-up LUS exams to evaluate if their condition deteriorates to a “non-focal” ARDS. * ventilation strategy in Table 1. *ARDS* acute respiratory distress syndrome, *LUS* lung ultrasound

Patients assigned to the intervention group will have their ventilator settings adjusted based on the lung morphology (Table 1). A detailed handbook for mechanical ventilation in study participants can be found in supplement 2. In “focal” patients randomized to the personalized ventilation group, a LUS exam will be repeated every 48–72 h to assess whether they have developed “non-focal” ARDS during their ICU stay. When a patient classified as “focal” develops “non-focal” ARDS, the settings are adjusted to the “non-focal” group (Fig. 2, Table 1).

### Criteria for discontinuing or modifying allocated interventions {11b}

Criteria for discontinuation or modification of the intervention targets are described in the supplement 2. The oxygenation, pressure, and ventilation targets can be loosened during interventions (e.g., bronchoscopy, placing the patient in the prone position) or modified when the patient is not ventilated in a lung-protective way (e.g., Pplat > 30 cm H_2_O). Specifically, in patients with “focal” ARDS and personalized ventilation, the PEEP can be increased when the FiO_2_ is higher than 80% and the PaO_2_/FiO_2_ is below 100 mmHg in the prone position for more than 6 h, the physician is allowed to set the PEEP above 9 cmH_2_O and recruitment maneuvers can be applied. In patients with “non-focal” ARDS, prone positioning can be applied when the PaO_2_/FiO_2_ is lower than 150 mmHg for 6 h in supine position and the FiO_2_ is higher than 80%. When a “non-focal” ARDS patient is breathing spontaneously, PEEP can be decreased to 10 cmH_2_O, and recruitment maneuvers are now only performed at the physician’s discretion. For patient-triggered breaths, the tidal volume margins are no longer required.

### Strategies to improve adherence to interventions {11c}

At every study site, there will be a dedicated study team that is trained by a member of the steering committee. Training consists of attending an initiation visit, reading standard operating procedures (SOP’s), and a study handbook. Furthermore, to mitigate the potential for misclassification in centers with limited LUS experience, we provide each center with comprehensive training via e-learning, including an exam and live support during the first two included patients. The Steering Committee provided a ventilation handbook for detailed guidance on how to ventilate patients according to the study protocol and other best practices in lung-protective mechanical ventilation to minimize heterogeneity in patient management within the study. The dedicated study team will give clinical lessons to all nurses and physicians before starting the trial. Furthermore, they will monitor the ventilation settings of included patients on a daily basis. A ventilator card with study guidelines will be placed at the bedside of the patient to enhance protocol adherence. If the ventilation parameters deviate from the protocol, a member of the study site is required to write a protocol deviation form. The protocol adherence will be evaluated in the pilot study and in every meeting with the Data and Safety Monitoring Board (DSMB).

### Relevant concomitant care permitted or prohibited during the trial {11d}

It is not allowed to perform LUS exams for the determination of the lung morphology outside of the study protocol. Furthermore, ventilator settings cannot be changed based on LUS exams that were performed outside of the study protocol. Other imaging modalities are permitted during the study. However, the classification of lung morphology conducted through the LUS exam is leading.

In controlled modes of ventilation, the default inspiration-to-expiration ratio will be 1:2. Expiratory time will be prolonged in case expiratory flow limitation is detected. The respiratory rate will be adjusted to obtain an arterial blood pH > 7.25 but preferably under 35 breaths per minute. The oxygenation target ranges for SpO_2_ and PaO_2_ are 88 to 95%, and 7.3 to 10.7 kPa, respectively [19].

The attending physician decides when to extubate a patient, based on general extubation criteria or with following a successful spontaneous breathing trial (SBT) with a T–piece or ventilation with minimal support (pressure support level < 10 cm H_2_O).

Early tracheostomy provides no advantage over late tracheotomy [20]. Therefore, tracheostomy is only to be performed on strict indications and preferably not earlier than 10 days after intubation. If a patient is treated with ECMO, the ventilator is set according to the local protocol for ventilation under ECMO. This means that the patient does not have to be ventilated according to the ventilation strategy of the randomization arm anymore. Sedation will follow the local guidelines for sedation in each participating unit. In general, these guidelines favor the use of analgo-sedation over hypno-sedation and use of bolus over continuous infusion of sedating agents. A Richmond Agitation Sedation Scale (RASS) score of − 2 to 0 is seen as adequate sedation [21, 22].

The routine use of neuromuscular blockage is not recommended. If neuromuscular blockade is required, single injections are preferred over continuous infusions.

If patients are expected to need ventilation for longer than 48 h and/or are expected to stay in the ICU for longer than 72 h, preventive measurements against VAP must be instituted according to the local guidelines.

A fluid balance targeted at normovolemia and diuresis of ≥ 0.5 ml/kg/h should be maintained with diuretics or by crystalloid infusions.

Thrombosis prophylaxis will be given according to local guidelines.

### Provisions for post-trial care {30}

There is no need for provisions for post-trial care after the patient is discharged from the ICU. The organization of study insurance depends on local regulations.

### Outcomes {12}

The primary endpoint is all-cause mortality at day 90 (diagnosis of ARDS considered as day 0). Secondary outcomes are mortality at day 28, VFD at day 28, ICU length of stay, ICU mortality, hospital length of stay, hospital mortality, and number of complications (VAP using the clinical pulmonary infection score (CPIS), pneumothorax, and need for rescue therapy). After a pilot phase, feasibility of LUS, correct interpretation of LUS images, and correct application of the intervention within the safe limits of mechanical ventilation is evaluated to inform a stop–go decision.

### Participant timeline {13}

**Fig. 3:**
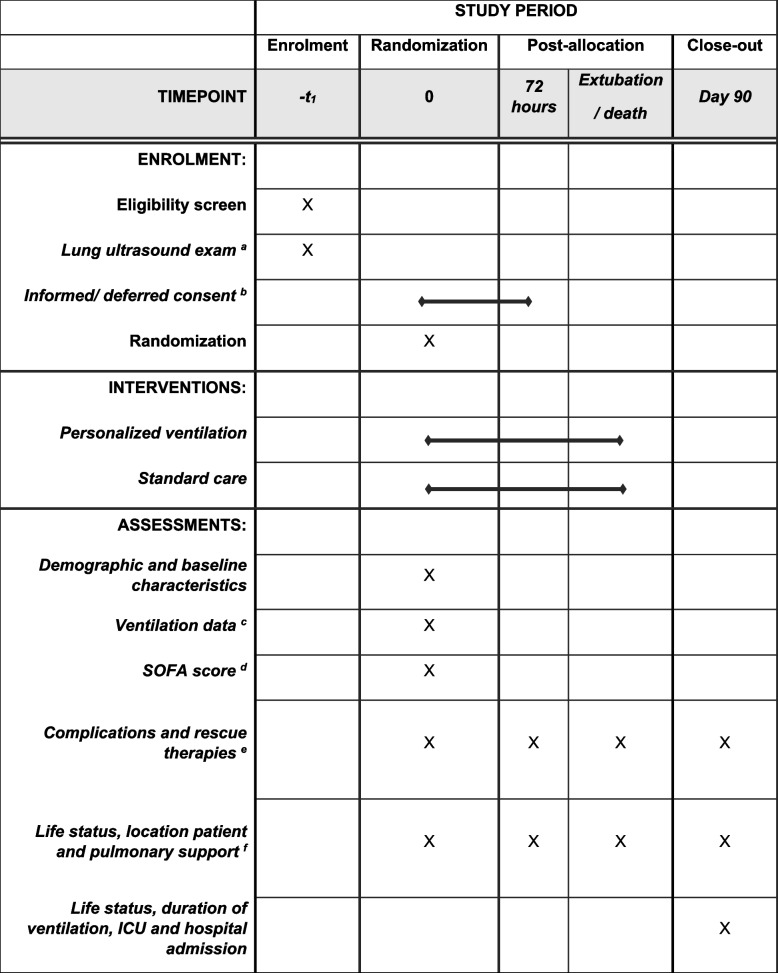
Timeline for a study participant from enrollment until last day of follow-up. ^a^If a patient has “focal” ARDS and is randomized to personalized ventilation, a LUS will be repeated every 72 h. ^b^Informed consent is obtained before randomization or deferred consent is obtained within 72 h after randomization depending on the local regulations. ^c^Until day 7 or until extubation. ^d^Until day 7 or ICU discharge. ^e^Until day 90 or ICU discharge. ^f^Until day 90 or hospital discharge. SOFA, Sequential Organ Failure Assessment; ICU, intensive care unit

The time points for enrolment, interventions, and follow-up can be found in Fig. 3.

### Sample size {14}

A sample of 538 patients (269 per group) is needed to detect an absolute between-group difference in 90-day mortality of 10% in favor of the intervention group, assuming a 27% mortality in the standard care, with a power of 80% at a two-tailed significance level of 0.047 [8]. In the sample size calculation, an interim analysis of the primary endpoint after the recruitment of 269 patients (using a *p*-value of 0.003) with alpha spending has been considered.

The first 80 included patients will be used for the pilot phase of the trial. At least 20 patients in each personalized group are necessary to assess clinical feasibility and protocol adherence. As the expected ratio between “focal” and “non-focal” and the ratio between the intervention and standard care is 1:1, we would need a sample size of 80 patients for this pilot study. We expect an interobserver agreement among experts of *κ*: 0.85 [16]. To be able to detect a clinically relevant decrease of *κ* towards 0.7 between experts and bedside clinicians, a total of 77 patients is needed for a power of 80% at a one-sided *α* level of 0.05. The primary endpoint will not be evaluated in the analysis of the pilot phase.

### Recruitment {15}

Given the above sample size of 538 patients and an expected inclusion rate of 1 patient per 2 months, the recruitment period is approximately 2 years after all 40 sites started enrolling patients [23]. To ensure the inclusion of patients from 40 centers, we will actively promote participation through conference announcements, via our website, and direct outreach to centers. Once a center begins enrolment, we will periodically request a consort figure to identify any missed patients and optimize the recruitment process accordingly.

## Assignment of interventions: allocation

### Sequence generation {16a}

Clinical research platform Castor Electronic Data Capture (EDC) (https://www.castoredc.com/) will be used to perform the randomization and is Good Clinical Practice (GCP) and Food and Drug Administration (FDA) compliant. Patients will be assigned to the personalized ventilation arm or to the control arm with a 1:1 ratio and only stratified by center.

### Concealment mechanism {16b}

Patients will be randomized in Castor EDC immediately after the LUS exam. Concealment will be ensured by the use of blocks with randomly permuted sizes.

### Implementation {16c}

The local principal investigator, who is trained by the steering committee, and the dedicated study time on the study site will enroll and randomize patients and make sure they will receive the ventilation strategy according to the randomization.

## Assignment of interventions: blinding

### Who will be blinded {17a}

The PEGASUS study is a non-blinded RCT as it is not feasible to blind the ventilation parameters to the treating team during ICU stay. The type of lung morphology of the patients in the standard of care group will be blinded to the treating team to prevent any bias in the standard care.

### Procedure for unblinding if needed {17b}

N/a. The PEGASUS study is a non-blinded RCT as it is not feasible to blind the ventilation parameters to the treating team during an ICU stay.

## Data collection and management

### Plans for assessment and collection of outcomes {18a}

All study data can be extracted from the paper or electronic patient record. Every center will have an initiation visit with a member of the steering committee before the inclusion of the first patient. In this visit, the data entry will be discussed to ensure the quality of the data entry. Furthermore, a SOP is provided containing detailed instructions. An extraction from the electronic case report form (eCRF) can be found in the supplement 3.

### Plans to promote participant retention and complete follow-up {18b}

All the data for the PEGASUS study can be extracted from the patient’s record. Therefore, we expect a very low percentage of patients with a lost to follow-up for the primary endpoint. To further minimize the lost to follow-up, we inform relatives during the deferred consent procedure about the follow-up procedure, researchers use a subject identification log with automatic follow-up reminders and the contact information of the research team is available on every consent form.

### Data management {19}

Data quality of the PEGASUS study will be guaranteed through calculations and range checks in our database. Furthermore, data cleaning will be performed after closing the database, which will be described in our statistical analysis plan. A data management plan can be requested from the steering committee. All data will be stored for the length of the study and for 15 years afterwards.

### Confidentiality {27}

Data of patients will be stored under a patient identification code that is not based on personal data. The codebook will be stored digitally and will be encrypted with a double password. The paper version will be stored behind a lock. All handling of personal data will comply with the General Data Protection Regulation (GDPR). Data can be shared between participating hospitals. Only coded information will be shared using data sharing systems developed for sharing medical data.

### Plans for collection, laboratory evaluation, and storage of biological specimens for genetic or molecular analysis in this trial/future use {33}

N/a. The PEGASUS study does not collect or analyze any biological specimens for genetic or molecular analysis in the current trial and for future use in ancillary studies.

## Statistical methods

### Statistical methods for primary and secondary outcomes {20a}

The statistical analysis will be based on the intention-to-treat principle, with patients analyzed according to their assigned treatment arms, except for cases lost to follow-up, or patients who are withdrawn due to lack of deferred informed consent. In addition, we will conduct per-protocol analyses, which only consider patients who completed the treatment according to the originally allocated protocol. Before the end of recruitment, a detailed statistical plan will be published.

When appropriate, statistical uncertainty will be expressed by the 95% confidence levels. *P*-value under 0.047 will be considered statistically significant for the primary endpoint and a *p*-value under 0.05 for secondary endpoints. Normality of data distribution will be assessed by visual inspection of histograms. For the experimental and control arms, continuous normally distributed variables will be expressed by their mean and standard deviation (SD) or, when not normally distributed, as medians and their interquartile ranges (IQR). Categorical variables will be expressed as frequencies and percentages. If less than 5% of data are missing or unavailable, no imputation data will be applied. All statistical analyses will be described in full detail in a statistical analysis plan, which will be published before the database is locked and analysis starts. Analysis will be performed with R software.

The goal of the primary analysis is to quantify the effect of LUS guided personalized mechanical ventilation in comparison with standard care on the 90-day mortality (with day of ARDS diagnosis as 0). The odds ratio for 90-day mortality is calculated using logistic regression analysis with mortality as dependent variable and randomization group as independent variable. Adjusted analysis will be performed according to European Medicines Agency (EMA) and FDA guidelines with the strongly prognostic variables age, clinical frailty, and PaO_2_/FiO_2_ at admission as covariables [24]. The stratification variable (center) will be included as a random effect.

Since ventilator-free days is a highly skewed variable with a peak in − 1 due to 28-day mortality, the mean ratio will be estimated using a generalized additive model for location scale and shape (GAMLSS) considering a zero-inflated and transformed beta distribution and using the delta method to estimate the confidence interval. A competing risk proportional hazard models will be used to evaluate the difference in time to extubation (accounting for mortality as a competing risk). Differences between groups in continuous variables will be analyzed with Student’s *t-*test or the Mann–Whitney *U*. Categorical variables will be compared with the chi-squared test or Fisher’s exact test, as appropriate. Mortality rates and length of ICU and hospital stay will be compared using Kaplan–Meier mortality curves.

To assess interobserver agreement, an expert panel will score all LUS exams to assess and evaluate the clinicians’ diagnostic accuracy of distinguishing “focal” ARDS from “non-focal” ARDS by using Fleiss’ *κ*. The expert panel will be blinded to the randomization group and clinical parameters of the patient while scoring the LUS exams. Ventilator settings, complications, and the use of adjunctive strategies such as prone position and recruitment maneuvers will be summarized per morphology and randomized group.

### Interim analyses {21b}

The study can be ended prematurely by the steering committee based on recommendations of the DSMB, for example as a result of low recruitment. There is a formal stopping rule after the first interim analysis. If the threshold of *P* = 0.003 is passed in favor of either of the treatment arms, the study is automatically ended. In case the study is ended prematurely, the sponsor will notify the accredited institutional review board (IRB) within 15 days, including the reasons for the premature termination. Within 1 year after the end of the study, the investigator/ sponsor will submit a final study report with the results of the study, including any publications/abstracts of the study, to the accredited IRB.

### Methods for additional analyses (e.g., subgroup analyses) {20b}

A predefined subgroup analysis stratified per morphology subphenotype (“focal” and “non-focal”) will be performed between the randomization arms for all primary and secondary outcomes.

### Methods in analysis to handle protocol non-adherence and any statistical methods to handle missing data {20c}

Protocol non-adherence will be monitored by the DSMB and published in a pilot study after recruiting the first 80 patients. When the protocol adherence is not sufficient, patient recruitment will be stopped. The percentage of missing data is expected to be low as almost all data needed for the study can be extracted from the patient record. If data is missing for the primary endpoint, we will perform a complete cases analysis. For other all data points, the primary method to handle missing data will be multiple imputation.

### Plans to give access to the full protocol, participant-level data, and statistical code {31c}

On reasonable request to a member of the steering committee the dataset, protocol and statistical code will be shared.

## Oversight and monitoring

### Composition of the coordinating center and trial steering committee {5d}

The steering committee of the PEGASUS study consists of six researchers, all of whom have their different field of specialty and carry their responsibility for this specific topic in the study (e.g., imaging, clinical trials, invasive ventilation, methodology, and statistics). The steering committee will meet regularly with the national coordinators to update them about the study. There will be one national coordinator for every country and he/she will be responsible for training the local investigators. The local investigator oversees the daily work of conducting the study in their hospital.

### Composition of the data monitoring committee, its role and reporting structure {21a}

A DSMB is installed to monitor safety and the overall conduct of the trial. The DSMB consists of four individuals with one as the chair. The DSMB will first meet after inclusion of the first 80 patients in the pilot phase, approximately 6 months after the first patient is enrolled. Subsequent to this meeting, the DSMB will meet virtually every 6 months. The DSMB will review the overall status of the study, the number of patients enrolled and adherence to the protocol (in total and per center). The DSMB will monitor safety of both ventilation strategies by monitoring the secondary endpoints of ventilation specific complications. The report and/or advice of the DSMB will be sent to the sponsor of the study and to the IRB of the Amsterdam UMC.

### Adverse event reporting and harms {22}

The risks of the PEGASUS study are considered minimal as the ventilation methods in the intervention group are already being applied in the standard care of ARDS patients. For this reason, we are not expecting serious adverse events (SAEs) related to the study. Therefore, we report secondary endpoints of this trial, which incorporate ventilation-specific complications, in a line listing two times per year to the IRB to monitor safety of both treatment strategies. The IRB will receive a line listing of the secondary endpoints incorporating ventilation-specific complications (incidence of pneumothorax and VAP) and ICU mortality. These endpoints will be specified per study arm in the line listing without disclosing the specific arms.

### Frequency and plans for auditing trial conduct {23}

On-site monitoring will be performed by an independent monitor or a researcher at all participating sites. The roll of the monitor is to verify the completeness and correctness of the research dossier and the presence of complete informed consent forms. The monitors will follow an approved monitoring plan. A monitor visit must be performed at least when the first 5 patients are included and at the end of the trial.

### Plans for communicating important protocol amendments to relevant parties (e.g., trial participants, ethical committees) {25}

Every substantial amendment must be reported and approved by the ethical board of the sponsor. Every participating center and other relevant parties will be notified of the approval of an amendment by e-mail and trial registries will be updated.

### Dissemination plans {31a}

The results of the study will find their way into (inter-) national scientific journals and guidelines. Every participating center is encouraged to submit substudies and to publish these after approval of the sponsor and publication of the main article. Participants can find the results of the study on https://clinicaltrials.gov/ under the name PEGASUS or with the identifier (NCT05492344).

## Discussion

Identifying ARDS subphenotypes based on “focal” or “non-focal” lung morphology has the potential to better tailor mechanical ventilation strategies for individual patients. The PEGASUS study is the first RCT that compares personalized ventilation guided by LUS with conventional ventilation in invasively ventilated patients with moderate and severe ARDS.

Several studies support the rationale behind the ventilation strategies for the “focal” and “non-focal” ARDS subphenotypes [7, 8, 25]. Patients with “non-focal” ARDS showed to respond better to recruitment maneuvers in comparison to patients with “focal” ARDS in an observational study. Notably, in the latter group, there was a significant higher risk for hyperinflation of the healthy lung tissue during these recruitment maneuvers [7]. In the LIVE study, aligning tidal volumes, PEEP, prone positioning, and recruitment to “focal” and “non-focal” subphenotypes demonstrated the potential of personalized ventilation to decrease mortality in ARDS patients with accurately classified morphology. The beneficial effect of personalized ventilation in the LIVE study was masked by the large proportion of patients with misclassified lung morphology, an issue that the PEGASUS study aims to address [8]. Findings from a recent retrospective study support the distinct tidal volume targets for the “focal” and “non-focal” subphenotype, as this study found an association between driving pressure and mortality in “non-focal” patients, but not in “focal” patients [25].

A major strength of the present study is the use of LUS to assess lung morphology, as it is a widely available, bedside, non-invasive technique that can accurately classify lung morphology compared to gold standard chest CT [16, 26]. A second advantage is that the ventilation strategies in both study arms are highly standardized and feasible. The personalized strategy is based on the LIVE study, while the conventional strategy is based on the latest ESICM guidelines for respiratory support in ARDS [27]. The ESICM guidelines do not provide specific recommendations for PEEP titration. We therefore adopted the low PEEP/high FiO_2_ table of the ALVEOLI study, which is consistent with the LIVE study, is easy to use, and overlaps with both personalized strategies [18]. Furthermore, we allow for rescue therapies in all study arms to not withhold potentially beneficial interventions in ARDS patients experiencing prolonged inadequate oxygenation within the assigned ventilation strategy. Third, we use an objective, patient-centered outcome as primary endpoint, namely all-cause mortality at day 90. The primary endpoint has been previously employed in the LIVE study, facilitating a robust calculation of the sample size for our study. Finally, the results of the current study will be generalizable due to the large number of participating ICUs both within and outside the European Union. These ICUs have varied experience in LUS and are situated in countries with diverse economic circumstances.

A potential limitation of the study is that clinical teams are not blinded for the intervention due to the nature of the study. However, treating physicians will remain blinded to the morphology subphenotype in patients randomized to the control group. A second limitation is the possibility that “focal” and “non-focal” patients do not benefit from the personalized ventilation strategy to the same extent. While the impact of personalized ventilation appears similar across morphology subphenotypes in the LIVE study, and while we will investigate potential differences in response through preplanned subanalyses, the study is not powered to detect the effect of personalized versus control in the two morphology subphenotype groups separately.

In conclusion, we test the hypothesis that personalized ventilation based on lung morphology guided by LUS can significantly reduce the mortality in mechanically ventilated patients with ARDS in comparison with conventional ventilation. If the anticipated mortality benefit is found, the findings of this study have the potential to change the ventilation strategy in ARDS patients from a one-size-fits-all to a tailored approach.

## Trial status

The PEGASUS study (protocol version 4, 02–11-2022) started recruiting patient on the 8th of august 2022 and is still recruiting. We expect to include the last patient in the trial on the first of January 2026*.*

### Supplementary Information

Supplementary Material 1.

Supplementary Material 2.

Supplementary Material 3.

## Data Availability

All study sites will have access to the database after the inclusion of the last patient. The details regarding confidentiality, data protection, intellectual property, and publication are documented in a clinical trial agreement that is signed before the inclusion of the first patient.

## Abbreviations

ARDS: Acute respiratory distress syndrome
LUS: Lung ultrasound
RCT: Randomized clinical trial
ICU: Intensive care unit
VFD: Ventilator-free days
VAP: Ventilator-associated pneumonia
Amsterdam UMC: Amsterdam University Medical Centres
MORU: Mahidol Oxford Tropical Medicine Research Unit
LEICA: Laboratory of Experimental Intensive Care and Anaesthesiology
PaO_2_: Partial pressure of oxygen in the arterial blood
FiO_2_: Fraction of inspired oxygen
PEEP: Positive end-expiratory pressure in the airway
CT: Computed tomography
CXT: Chest X-ray
ECMO: Extracorporeal membrane oxygenation
ESICM: European Society of Intensive Care Medicine
PBW: Predicted body weight
DSMB: Data and safety monitoring board
SBT: Spontaneous breathing trial
RASS: Richmond Agitation Sedation Scale
SOFA: Sequential Organ Failure Assessment
EDC: Electronic data capture
GCP: Good Clinical Practice
FDA: Food and Drug Administration
SOP: Standard operating procedure
eCRF: Electronic case report form
GDPR: General Data Protection Regulation
SD: Standard deviation
IQR: Interquartile ranges
EMA: European Medicines Agency
GAMLSS: Generalized additive model for location scale and shape
Ppeak: Peak airway pressure
Pmax: Maximal airway pressure
IRB: Institutional review board
SAEs: Serious adverse events
CPIS: Clinical pulmonary infection score
