# Supplementary material for: Personalized mechanical ventilation guided by ultrasound in patients with acute respiratory distress syndrome (PEGASUS): study protocol for an international randomized clinical trial
Source: Trials. 2024 May 7;25:308. doi: 10.1186/s13063-024-08140-7 (PMC11077821; doi:10.1186/s13063-024-08140-7)
Supplement: Supplementary file 1 — Supplementary Material 1. [file 13063_2024_8140_MOESM1_ESM.pdf]

Standard operating procedure: Lung Ultrasound for Classification of Lung Morphology

Audience: intensivist, physicians, nurses and researchers.

Standard operating procedure (SOP) applicable to: all principal investigators and his/her delegated investigators involved in the screening, randomization and inclusion of patients in the PEGASUS study.

Study:

The PEGASUS study is a randomized controlled trial, comparing personalized ventilation with standard ventilation in ARDS patients. The primary endpoint is all-cause mortality at day 90.

Contact person:

Jante Sinnige mobile: +31637405311

j.s.sinnige@amsterdamumc.nl

The E-learning for performing the lung ultrasound can be on our website [E-learning of PEGASUS study | ICU Research].

Start Lung ultrasound exam

1. Start the lung ultrasound (LUS) machine.
2. Choose the linear probe, use of other probes is allowed when use of the linear probe does not result in assessable LUS images.
3. Choose the lung setting on the ultrasound machine. Most ultrasound machines have software that will minimize artefacts. For the assessment of the lungs, we would like to preserve these artefacts.
4. Place the patient in a semi recumbent position (figure 1) and place the arms of the patient. If the patient was just placed from a prone to a supine position, please wait 30 minutes before the start of the LUS exam to mobilize fluid that has moved to the anterior lung fields due to the prone positioning towards the posterior lung fields. Position the patient's arms as shown in figure 2. This makes performing the ultrasound easier.

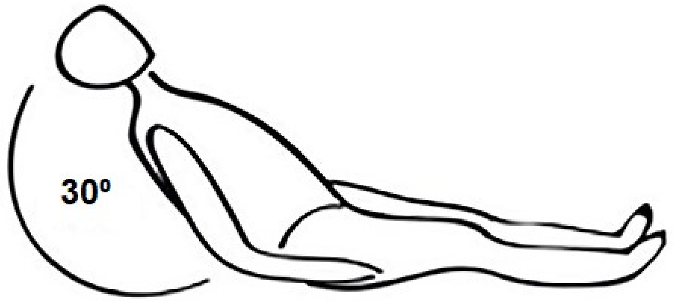

Figure 1. Semi recumbent position.

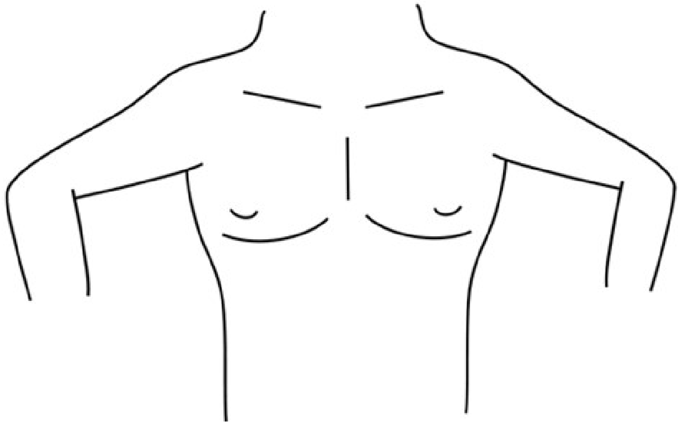

Figure 2. Placing of the arms of the patient.

5. After pre-oxygenation and increasing the FiO_2, set the PEEP to 5 cm H_2O. If too much desaturation is expected with a PEEP of 5 cm H_2O, the LUS exam can also be performed at a maximum PEEP level of 8 cm H_2O.

6. Start the LUS exam by placing the probe longitudinal on region of interest with the pointer facing towards the cranial side of the patient (figure 3).

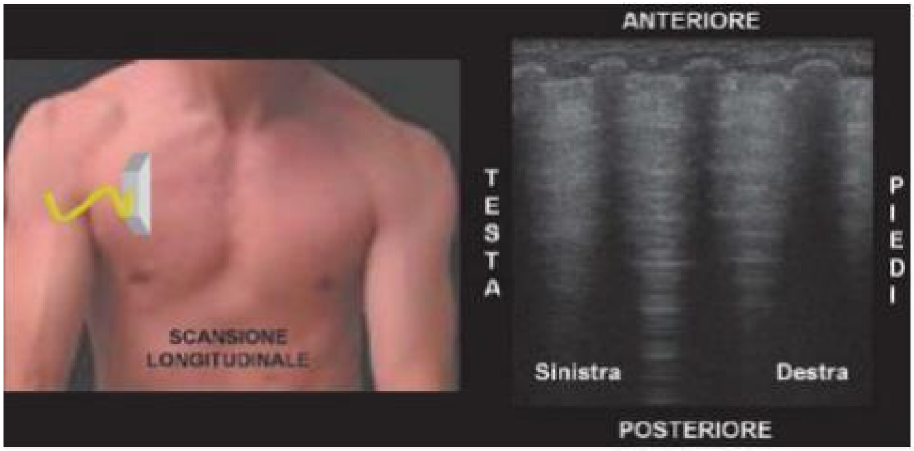

Figure 3 – Longitudinal position of the probe

7. Identify the pleural line between the ribs.

8. Set the depth at 10 cm, then adjust it in order to have the pleural line at 1/3 of the screen.

9. Focus should be on pleural line or on the point of interest if any alteration (i.e. consolidation).

10. Gain adjusted such that the pleural line is clearly visible but not overexposed.

11. Turn the probe with 90 degrees in which the pointer will face te right side of the patient (figure 4).

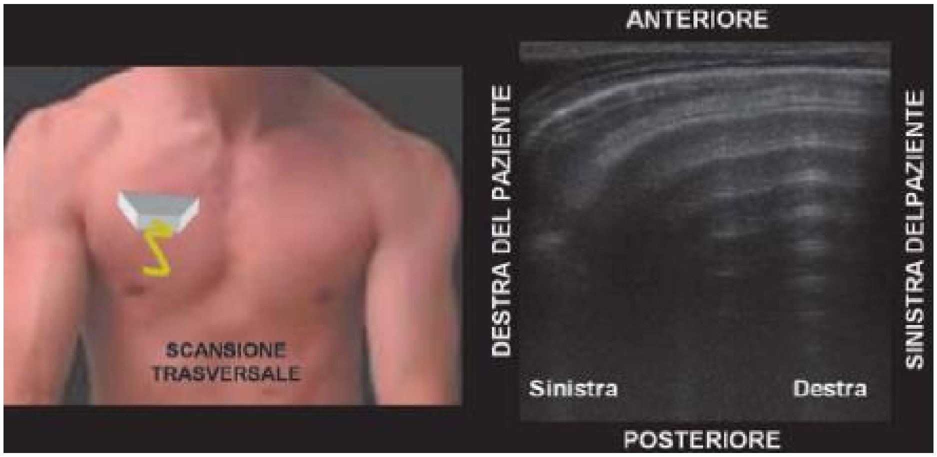

Figure 4 – Transversal position of the probe

12. Asses the lung aeration score of the region using the score below.

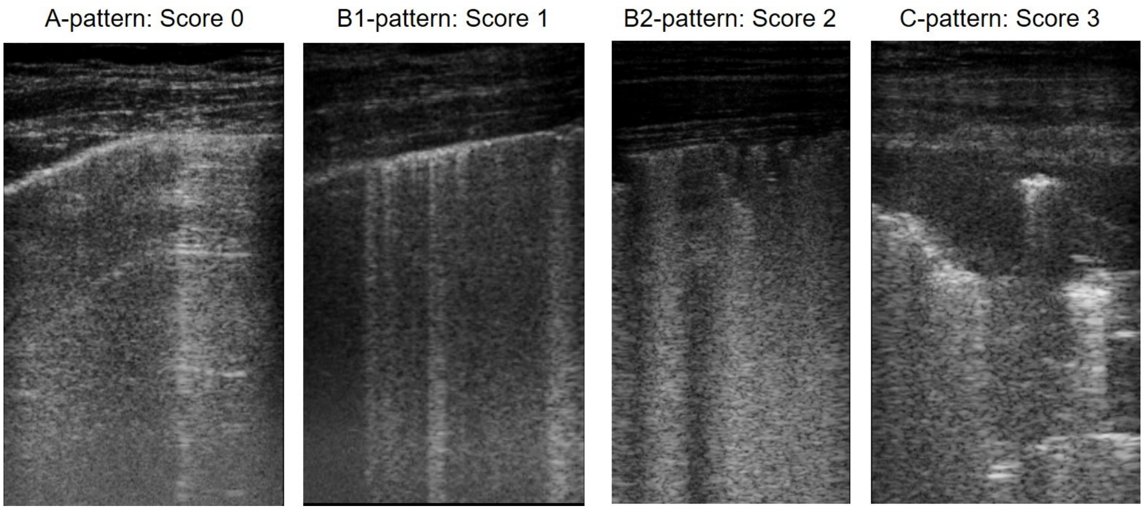

Figure 5. Score 0: "A-pattern" (i.e., repeating horizontal A-lines parallel to the pleural line, suggesting normal aeration). Score 1: a "B-pattern" (i.e., three or more vertical B-lines starting from the pleural line and reaching the bottom of the screen, suggesting partial loss of aeration) and B-lines are well-spaced and cover ≤ 50% of the pleural line, Score 2: if B-lines cover ≥ 50% of the pleural line. Score 3: a "C-pattern" when the consolidation is > 2cm (i.e., consolidation, suggesting near-complete to complete loss of aeration).

13. Save the image as a clip when the image is of sufficient quality to score lung aeration.

14. Repeat this for the twelve different regions of the lungs, six locations for each hemithorax, shown on the picture below (figure 6).

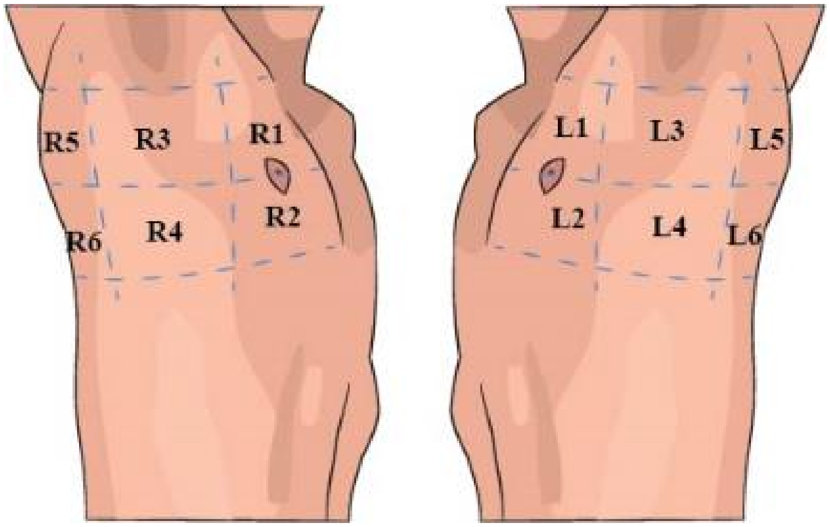

Figure 6 – Twelve regions to perform the LUS.

15. When all regions have been scored, the results can be entered into the Castor database. This will automatically assess the lung morphology phenotype by using the flow diagram below (figure 7).

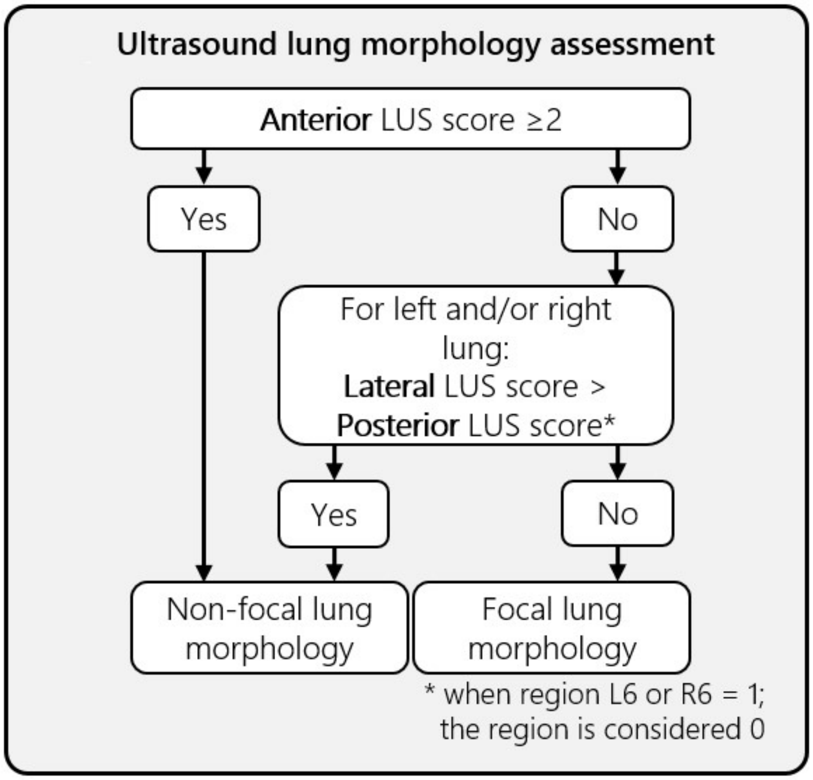

Figure 7 – Assessment of lung morphology.

16. If due to any reason a region could not have been scored, the lung morphology phenotype of the patient must be assessed by the investigator him-/herself. The missing score can be complemented by using the scores of the other fields in the same area (anterior 1&2, lateral 3&4 or posterior 5&6). The missing region needs to be complemented for the morphology classification, but in the Castor database the region should be registered as missing.

17. For example, if region R2 is missing and the scores of the other anterior regions are known (for example L1 = 1, L2 = 0, R1 = 2) take the sum of the scores and divide it by the number of regions. Use the result for the missing region. In this case; (1 + 0 + 2)/3 = 1 = R2.

18. Please send the images to j.s.sinnige@amsterdamumc.nl

19. Apply the correct ventilator strategy depending on the randomization and ARDS phenotype of the patient.
