# Supplementary material for: Personalized mechanical ventilation guided by ultrasound in patients with acute respiratory distress syndrome (PEGASUS): study protocol for an international randomized clinical trial
Source: Trials. 2024 May 7;25:308. doi: 10.1186/s13063-024-08140-7 (PMC11077821; doi:10.1186/s13063-024-08140-7)
Supplement: Supplementary file 2 — Supplementary Material 2. [file 13063_2024_8140_MOESM2_ESM.pdf]

Handbook for mechanical ventilation

Study: The PEGASUS study is a randomized controlled trial, comparing lung ultrasound guided personalized ventilation with standard ventilation in ARDS patients. The primary endpoint is mortality at day 90.

Contact person:

Lieuwe Bos	l.d.bos@amsterdamumc.nl
Jante Sinnige	mobile: +31637405311
	j.s.sinnige@amsterdamumc.nl

Table of Contents

Structure to mechanical ventilation within the study protocol 3
Inclusion and randomization 4
  Phase 1. Determine lung morphology 4
  Phase 2. Randomize 4
Control group 5
  Phase 3. Initial settings 5
  Phase 4. Worsening condition 6
  Phase 5. Improving condition 7
  Phase 6. Switch to assisted MV 8
Personalized ventilation, focal ARDS 9
  Phase 3. Initial settings 9
  Phase 4. Worsening condition 10
  Phase 5. Improving condition 11
  Phase 6. Switch to assisted MV 11
Personalized ventilation, non-focal ARDS 12
  Phase 3. Initial settings 12
  Phase 4. Worsening condition 13
  Phase 5. Improving condition 14
  Phase 6. Switch to spontaneous MV 15
Weaning in all groups 16
  Phase 7. Extubation 16
  Phase 7. Weaning via tracheostomy 16
Other suggestions for standard care 18
Appendix 1: Recruitment maneuvers 20
Appendix 2: Abbreviations 21

Structure to mechanical ventilation within the study protocol

Within the PEGASUS study we can recognize seven phases as shown in Figure 1. To provide clinicians and researchers with additional guidance, we provide more details on each of these steps in this study-specific handbook of mechanical ventilation. As patients can have two types of lung morphology (focal / non-focal) and are treated according to two arms (control vs. personalized ventilation), there are three different ventilation strategies (control, personalized focal, personalized non-focal). These three different ventilation strategies come with differences in challenges and therefore phase 3, 4, 5 and 6 of the flowchart in Figure 1 are outlined for each strategy separately.

Figure 1: The seven phases in the PEGASUS study

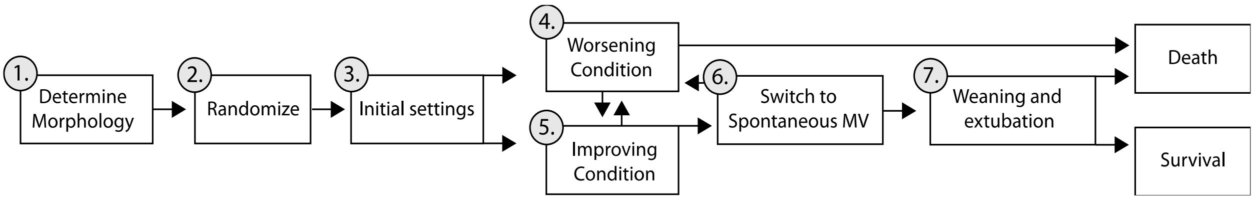

Inclusion and randomization

Phase 1. Determine lung morphology

Lung morphology is best determined at a PEEP of 5 cmH_2O. Therefore, the PEEP is decreased for the lung ultrasound exam in eligible patients.

- **!!!** If the PEEP is already above 5 cmH_2O and the patient becomes severely hypoxemic due to shunt when decreasing PEEP (*i.e.* $SpO_2 < 90\%$ despite $FiO_2 > 80\%$), perform the exam at the lowest PEEP possible. If the exam shows an anterior LUS score of 2 or more, this would be consistent with non-focal morphology. No further decrease in PEEP is necessary in this case as this will not influence the morphology because the lung will only become less aerated. If the LUS exam is consistent with focal morphology, try to further decrease PEEP and re-evaluate. Exclude the patient from the study if this is not possible and report a failure to screen.
- See *"SOP 2 PEGASUS LungUltrasound v1 – English"* for more details on the LUS exam and how to report this.

Phase 2. Randomize

See "*SOP 4 PEGASUS randomization v1 – English*"

On the following pages, we will discuss phase 3-6 for each intervention

Only read the pages related to the intervention the patient is receiving

The exclamation marks (!!!) indicate what strategies are different between study interventions

Control group

Phase 3. Initial settings

	Settings	Margins / specifics
**Mode of ventilation**	Pressure controlled or volume controlled	Automated modes are only allowed when FiO_2, minute volume and PEEP are set manually
**!!! Tidal volume**	6 mL/kg PBW	4 – 8 ml/kg PBW
**I:E ratio**	1:2	Avoid breath stacking and intrinsic PEEP, prolong expiratory time when observed
**!!! PEEP and FiO2**	PEEP/FiO_2 table (Table 1)	PEEP level +/- 3 cmH_2O OR FiO_2 +/- 10%<br>During interventions (e.g. bronchoscopy, placing patient in prone positioning) the FiO_2 can be raised for a period of time without raising the PEEP
**Plateau pressure**	<30 cmH_2O	Decrease Vt if above this limit and current Vt >6ml/kg PBW<br>Decrease PEEP if above this limit
**!!! Recruitment maneuver**	Only for rescue	See phase 4. Worsening condition
**!!! Prone positioning**	PaO_2/FiO_2 < 150 mmHg	When PaO_2/FiO_2 remains < 150 mmHg for more than 6 hours despite optimisation
**Oxygenation targets**	SpO_2 88 to 95%<br>PaO_2 7.3 to 10.7kPa	
**Decarboxylation and respiratory rate**	pH > 7.25	Preferably a respiratory rate under 35/min

Table 1: Strategy for initial setting and increase of FiO_2 and PEEP

FiO_2	0.3	0.4	0.4	0.5	0.5	0.6	0.7	0.7	0.7	0.8	0.9	0.9	0.9	1.0
**PEEP**	5	5	8	8	10	10	10	12	14	14	14	16	18	18-24

FiO_2 = Fraction of inspired oxygen, PEEP = positive end-expiratory pressure.

- !!! Patients assigned to the control group will be ventilated according to the current standard of care according to international guidelines. In these patients, the PEEP and FiO_2 level will be selected according to the low PEEP/ high FiO_2 ratio from the ALVEOLI study maintaining an end-inspiratory plateau pressure lower than 30 cmH_2O.
- Formula for calculating the tidal volume size with predicted body weight (PBW) are 50 + 0.91 x (centimeters of height – 152.4) for males and 45.5 + 0.91 x (centimeters of height – 152.4) for females.

Phase 4. Worsening condition

Pressure: Plateau pressure above 30cmH$_2$O

- If the tidal volume is above 6 ml/kg PBW, first decrease tidal volume to below 6 ml/kg PBW to maintain a plateau pressure of 30 cmH$_2$O (or less).
- The PEEP level should be decreased to maintain a plateau pressure of 30 cmH$_2$O (or less)

Oxygenation: Persistent PaO$_2$/FiO$_2$ ratio ≤ 150 mmHg after 6 hours of MV

- !!! Prone position is encouraged if PaO$_2$/FiO$_2$ ratio remains ≤ 150 mmHg for more than 6 hours after optimization of PEEP and FiO$_2$ according to Table 1 and is preferably applied for at least 16 hours a day.
- Any increase of PEEP and FiO$_2$ should be performed according to Table 1.
- For a tutorial on how to turn the patient into the prone position see: https://www.youtube.com/watch?v=E_6jT9R7WJs
- The effect of prone positioning should not be evaluated based on changes in gas exchange or respiratory dynamics as these are poor surrogates for treatment effect.
- We do not encourage routine use of neuromuscular blockade (NMB). If NMB is used, at the discretion of the treating physician, use bolus injections and re-evaluate.
- !!! Recruitment maneuvers are only advised as a last resource. When a recruitment maneuver is performed we advise to use the protocol described in Appendix 1.

Decarboxylation: *High PaCO$_2$ and low pH despite high respiratory rate*

- Aim for permissive hypercapnia (pH > 7.25)
- Consider measuring dead space ventilation.
- Reduce instrumental dead space in the ventilator circuit.
- !!! Consider optimizing PEEP to allow for better decarboxylation. This could mean that PEEP is decreased rather than increased.
- Consider air trapping and check intrinsic PEEP and airway resistance.
- Consider a bolus injection of neuromuscular blockade if the pH is still below 7.25

ECMO

- Patients can be considered for ECMO according to the local protocol. If a patient receives ECMO, the ventilator is set according to the local protocol for ventilation under ECMO. This means that PEEP is no longer titrated according to the study protocol.

Phase 5. Improving condition

Oxygenation: PaO_2/FiO_2 ratio > 150 mmHg in supine position

- **!!!** Stop prone positioning.

Oxygenation: SpO_2 > 95%

- **!!!** In this phase the low PEEP/high FiO_2 table (Table 1) is <u>not</u> used to set the ventilator. Do not decrease PEEP immediately but decrease FiO_2 first, to avoid alveolar collapse. We advise starting decreasing PEEP once the FiO_2 is decreased (for example below 40%). A suggested protocol for decreasing PEEP and FiO_2 can be found in Figure 2.

Oxygenation and respiratory mechanics

- Acceptance of assisted ventilation is tested three times a day in all patients who receive controlled ventilation and have improving condition.

Figure 2: <u>Protocol</u> for initial setting and increasing PEEP/FiO_2 and <u>advice</u> for decreasing PEEP/FiO_2

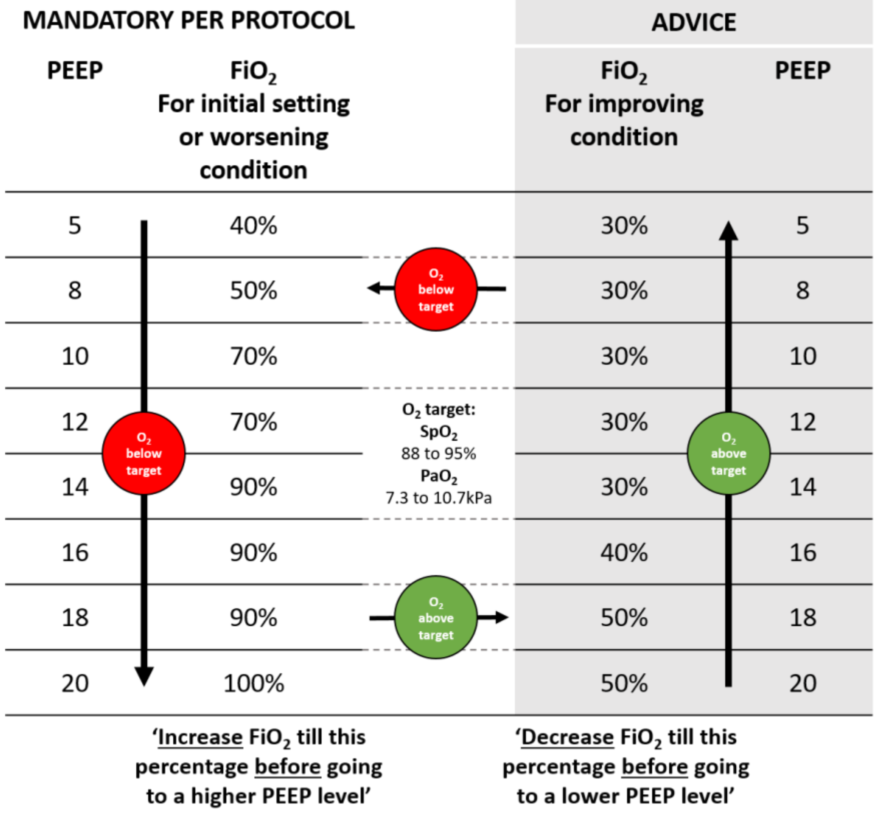

Phase 6. Switch to assisted MV

	Settings	Margins / specifics
**Mode of ventilation**	Pressure support	Automated modes are only allowed when FiO_2, minute volume and PEEP are set manually
**Tidal volume**	Dependent on the patients' effort	Aim for the lowest pressure support level.
**Rise time**	Highest possible	Adjust based on clinical insights
**Cycling off**	25%	Adjust based on clinical insights
**!!! PEEP**	First decrease FiO_2. Decrease PEEP once FiO_2 is decreased (for example below 40%)	
**Plateau pressure**	<30 cmH_2O	Decrease pressure support if more than 5 cmH_2O is given<br>Decrease PEEP if above this limit
**Recruitment maneuver**	Not applicable	
**!!! Prone positioning**	PaO_2/FiO_2 < 150 mmHg	When PaO_2/FiO_2 remains < 150 mmHg for more than 6 hours despite optimisation
**Oxygenation**	SpO_2 88 to 95%<br>PaO_2 7.3 to 10.7kPa	During interventions (e.g. bronchoscopy, placing patient in prone positioning) the FiO_2 can be raised above SpO_2 of 95%
**Decarboxylation and respiratory rate**	pH > 7.25	Preferably a respiratory rate under 35/min

Personalized ventilation, focal ARDS

Phase 3. Initial settings

	Settings	Margins / specifics
**Mode of ventilation**	Pressure controlled or volume controlled	Automated modes are only allowed when FiO_2, minute volume and PEEP are set manually
**!!! Tidal volume**	6 to 8 mL/kg PBW	
**I:E ratio**	1:2	Avoid breath stacking and intrinsic PEEP, prolong expiratory time when observed
**!!! PEEP**	≤ 9 cm H_2O	PEEP level as low as tolerated, up to 5 cmH_2O
**Plateau pressure**	<30 cmH_2O	Decrease tidal volume to below 6 ml/kg PBW if above this limit<br>Decrease PEEP if above this limit
**!!! Recruitment maneuver**	Only for rescue	See Phase 4. Worsening condition
**!!! Prone positioning**	In all patients	Daily prone positioning until clinical improvement with PaO_2/FiO_2 > 200 mmHg at a PEEP of 5 cmH_2O in supine position
**Oxygenation**	SpO_2 88 to 95%<br>PaO_2 7.3 to 10.7kPa	During interventions (e.g. bronchoscopy, placing patient in prone positioning) the FiO_2 can be raised above SpO_2 of 95%
**Decarboxylation and respiratory rate**	pH > 7.25	Preferably a respiratory rate under 35/min

- !!! Prone position is mandatory in all patients in focal personalized group at the day of inclusion and each subsequent day the PaO_2/FiO_2 ratio is ≤ 200 mmHg at a PEEP of 5 cmH_2O in supine position and is preferably applied for at least 16 hours a day.
- Formula for calculating the tidal volume size with predicted body weight (PBW) are 50 + 0.91 x (centimeters of height – 152.4) for males and 45.5 + 0.91 x (centimeters of height – 152.4) for females.

Phase 4. Worsening condition

Pressure: Plateau pressure above 30cmH$_2$O

- Decrease tidal volume to below 6 ml/kg PBW to maintain a plateau pressure of 30 cmH_2O (or less).
- The PEEP level should be decreased to maintain a plateau pressure of 30 cmH_2O (or less)

Oxygenation: Persistent PaO_2/FiO_2 ratio ≤ 100 mmHg and FiO_2 >80% after 6 hours of MV

- **!!!** If the FiO_2 is higher than 80% and the PaO_2/FiO_2 is below 100 mmHg in prone position for more than 6 hours the physician is allowed to set the PEEP above 9 cm H_2O.
- We do not encourage routine use of neuromuscular blockade (NMB). If NMB is used, at the discretion of the treating physician, use bolus injections and re-evaluate.
- Recruitment maneuvers are only advised as a last resource. When a recruitment is performed, we advise to use the protocol described in Appendix 1.

Decarboxylation: *High $PaCO_2$ and low pH despite high respiratory rate*

- Consider measuring dead space ventilation
- Reduce instrumental dead space in the ventilator circuit.
- Consider optimizing PEEP to allow for better decarboxylation.
- Consider air trapping and check intrinsic PEEP and airway resistance.
- Consider a bolus injection of neuromuscular blockade if the pH is still below 7.25

ECMO

- Patients can be considered for ECMO according to the local protocol. If a patient receives ECMO, the ventilator is set according to the local protocol for ventilation under ECMO. This means that PEEP is no longer titrated according to the study protocol.

Phase 5. Improving condition

Oxygenation: PaO_2/FiO_2 ratio > 200 mmHg in supine position with a PEEP at 5 cm H_2O

- **!!!** Stop prone positioning

Oxygenation: SpO_2 > 95%

- **!!!** Decrease FiO_2 to keep PaO_2 and/or SpO_2 in range.

Oxygenation and respiratory mechanics

- Acceptance of assisted ventilation is tested three times a day in all patients who receive controlled ventilation and have improving condition.

Phase 6. Switch to assisted MV

	Settings	Margins / specifics
**Mode of ventilation**	Pressure support	Automated modes are only allowed when FiO_2, minute volume and PEEP are set manually
**Tidal volume**	Dependent on the patients' effort	Aim for the lowest pressure support level
**Rise time**	Highest possible	Adjust based on clinical insights
**Cycling off**	25%	Adjust based on clinical insights
**!!! PEEP**	≤ 9 cm H_2O	PEEP level as low as tolerated, up to 5 cmH_2O
**Plateau pressure**	<30 cmH_2O	Decrease pressure support if more than 5 cmH_2O is given<br>Decrease PEEP if above this limit
**Recruitment maneuver**	Not applicable	
**!!! Prone positioning**	PaO_2/FiO_2 < 200 mmHg	Daily prone positioning until clinical improvement with PaO_2/FiO_2 > 200 mmHg at a PEEP of 5 cmH_2O in supine position
**Oxygenation**	SpO_2 88 to 95%<br>PaO_2 7.3 to 10.7kPa	During interventions (e.g. bronchoscopy, placing patient in prone positioning) the FiO_2 can be raised above SpO_2 of 95%
**Decarboxylation and respiratory rate**	pH > 7.25	Preferably a respiratory rate under 35/min

Personalized ventilation, non-focal ARDS

Phase 3. Initial settings

	Settings	Margins / specifics
**Mode of ventilation**	Pressure controlled or volume controlled	Automated modes are only allowed when FiO_2, minute volume and PEEP are set manually
**!!! Tidal volume**	4 to 6 mL/kg PBW	
**I:E ratio**	1:2	Avoid breath stacking and intrinsic PEEP, prolong expiratory time when observed
**!!! PEEP**	≥ 15 cm H_2O	
**Plateau pressure**	<30 cmH_2O	Decrease Vt if above this limit and currently tidal volumes >4ml/kg PBW<br>Decrease PEEP if above this limit
**!!! Recruitment maneuver**	Daily in all patients	Use RM protocol
**!!! Prone positioning**	Rescue	When PaO_2/FiO_2 < 150 mmHg with FiO_2 ≥ 80% despite optimisation of mechanical ventilation > 6 hours
**Oxygenation**	SpO_2 88 to 95%<br>PaO_2 7.3 to 10.7kPa	During interventions (e.g. bronchoscopy, placing patient in prone positioning) the FiO_2 can be raised above SpO_2 of 95%
**Decarboxylation and respiratory rate**	pH > 7.25	Preferably a respiratory rate under 35/min

- !!! A recruitment maneuver mandatory in all patients in non-focal personalized group at the day of inclusion and each subsequent day the PaO_2/FiO_2 ratio is ≤ 200 mmHg in supine position or is switched to an assisted mode of MV.
- !!! Recruitment protocol:

1. The RM is performed by a qualified person, i.e., an intensivist of an intensive care doctor with sufficient experience;
2. Set PEEP at a minimum of 15 cm H_2O; if PEEP was not yet at 15 cm H_2O, it is increased in steps of 1 to 2 cm H_2O, wherein each steps last at least 10 seconds to see if the blood pressure remains acceptable. hemodynamic instability occurs; if PEEP is already > 15 cm H_2O, it is left unchanged;
3. Perform an inspiratory hold of 10 seconds by pressing the inspiratory hold button for 10 seconds; closely monitor the blood pressure, as if it drops the

rescue maneuver is stopped to take measure to ensure hemodynamic stability (e.g., by raising the dose of vasoactive medication);

4. In successive steps, set the upper airway pressure 15 cm H_2O above PEEP, followed by an inspiratory hold of 10 seconds by pressing the inspiratory hold button for 10 seconds; closely monitor the blood pressure, as if it drops the rescue maneuver is stopped to take measure to ensure hemodynamic stability (e.g., by raising the dose of vasoactive medication);
5. This maneuver is repeated 3 times
6. At PEEP of 15 cm H_2O, the upper airway pressure is set in such a way that the tidal volumes again correspond to the ventilation settings of the randomization arm.

- Formula for calculating the tidal volume size with predicted body weight (PBW) are 50 + 0.91 x (centimeters of height – 152.4) for males and 45.5 + 0.91 x (centimeters of height – 152.4) for females.

Phase 4. Worsening condition

Pressure: Plateau pressure above 30cmH_2O

- If the tidal volume is above 4 ml/kg PBW, first decrease tidal volume to 4 ml/kg PBW to maintain a plateau pressure of 30 cmH_2O (or less).
- The PEEP level should be decreased to maintain a plateau pressure of 30 cmH_2O (or less)

Oxygenation: Persistent PaO_2/FiO_2 ratio ≤ 150 mmHg with FiO_2 ≥ 80% after 6 hours of MV

- **!!!** Prone position is encouraged if PaO_2/FiO_2 ratio remains ≤ 150 mmHg with FiO_2 ≥ 80% for more than 6 hours after optimization of PEEP and is preferably applied for at least 16 hours a day.
- For a tutorial on how to turn the patient into the prone position see: https://www.youtube.com/watch?v=E_6jT9R7WJs
- The effect of prone positioning should not be evaluated based on changes in gas exchange or respiratory dynamics as these are poor surrogates for treatment effect.

- We do not encourage routine use of neuromuscular blockade (NSB). If NMB is used, at the discretion of the treating physician, use bolus injections and re-evaluate.

Decarboxylation: *High $PaCO_2$ and low pH despite high respiratory rate*

- Consider measuring dead space ventilation
- Reduce instrumental dead space in the ventilator circuit.
- Consider optimizing PEEP to allow for better decarboxylation. This could mean that PEEP is decreased rather than increased.
- Consider air trapping and check intrinsic PEEP and airway resistance.
- Consider a bolus injection of neuromuscular blockade if the pH is still below 7.25

ECMO

- Patients can be considered for ECMO according to the local protocol. In the event that a patient receives ECMO, the ventilator is set according to the local protocol for ventilation under ECMO. This means that PEEP is no longer titrated according to the study protocol.

Phase 5. Improving condition

Oxygenation: PaO_2/FiO_2 ratio > 200 mmHg in supine position

- **!!!** Stop recruitments

Oxygenation: PaO_2/FiO_2 ratio > 200 mmHg and FiO_2 below 40% in supine position

- **!!!** PEEP can be decreased (<15 cm H_2O).

Oxygenation: SpO_2 > 95%

- **!!!** Decrease FiO_2 to keep PaO_2 and/or SpO_2 in range.

Oxygenation and respiratory mechanics

- Acceptance of assisted ventilation is tested three times a day in all patients who receive controlled ventilation and have improving condition.

Phase 6. Switch to spontaneous MV

	Settings	Margins / specifics
**Mode of ventilation**	Pressure support	Automated modes are only allowed when FiO_2, minute volume and PEEP are set manually
**Tidal volume**	Dependent on the patients' effort	Aim for the lowest pressure support level
**Rise time**	Highest possible	Adjust based on clinical insights
**Cycling off**	25%	Adjust based on clinical insights
**!!! PEEP**	First decrease FiO_2, then further decrease PEEP after FiO_2 is 40% or less	PEEP level as low as tolerated, up to 5 cmH2O
**Plateau pressure**	<30 cmH_2O	Decrease pressure support if more than 5 cmH_2O is given<br>Decrease PEEP if above this limit
**Recruitment maneuver**	Not applicable	
**!!! Prone positioning**	PaO_2/FiO_2 < 150 mmHg	When PaO_2/FiO_2 < 150 mmHg with FiO_2 ≥ 80% despite optimisation of mechanical ventilation > 6 hours
**Oxygenation**	SpO_2 88 to 95%<br>PaO_2 7.3 to 10.7kPa	During interventions (e.g. bronchoscopy, placing patient in prone positioning) the FiO_2 can be raised above SpO_2 of 95%
**Decarboxylation and respiratory rate**	pH > 7.25	Preferably a respiratory rate under 35/min

Weaning in all groups

Phase 7. Extubation

- If the patient is on assisted ventilation, the groups converge to a low PEEP, low FiO_2 strategy. If extubation is expected within 48 hours, the study specific ventilation strategies are released.
- The attending physician decides when to extubate a patient, based on general extubation criteria (i.e. responsive and cooperative, adequate cough reflex, adequate oxygenation with $FiO_2 \leq 0.4$, hemodynamically stable, no uncontrolled arrhythmia and a rectal temperature > 36 Celsius and after successfully passing a spontaneous breathing trial (SBT) with a T–piece or ventilation with minimal support (pressure support level less than 10 cm H_2O, we suggest to use 5 cm H_2O).
- In case SBTs are used, an SBT is judged as successful when the following criteria are met for at least 30 minutes, the attending physician takes the final decision for extubation
  - Respiratory rate < 35/min
  - Peripheral oxygen saturation > 90%
  - Increase < 20% of Heart rate and blood pressure
  - No signs of anxiety and diaphoresis
- In case a patient needs to be re–intubated and ventilated, the ventilator settings are set back to levels appropriate for the intervention arm.

Phase 7. Weaning via tracheostomy

- Early tracheostomy has no advantage over late tracheotomy. Therefore, tracheostomy is only to be performed on strict indications and preferably not earlier than 10 days after intubation. Strict indications for tracheostomy:
  - Expected duration of ventilation > 14 days
  - Glasgow Coma Score < 7 and/or inadequate swallow or cough reflex with retention of sputum
  - Severe ICU–acquired weakness

- Repeated respiratory failure after extubation
- Pre–existent diminished pulmonary reserves
- Failure to intubate
- Prolonged or unsuccessful weaning

- Weaning with a tracheostomy follows recommendations as described under 'weaning'.
- The following suggested scheme can be used for unassisted ventilation with a tracheostomy, but should be individualized in every patient:

1. Unassisted ventilation for 30 minutes, three times per day
2. Unassisted ventilation for 1 hour, three times per day
3. Unassisted ventilation for 2 hours, three times per day
4. Unassisted ventilation for 4 hours, three times per day
5. Unassisted ventilation for 6 hours, two times per day
6. Unassisted ventilation for 18 hours
7. Unassisted ventilation for 24 hours

Other suggestions for standard care

Sedation protocol

- Sedation follows the local guidelines for sedation in each participating unit. In general, the use of analgo–sedation is favored over hypno–sedation, use of bolus is favored over continuous infusion of sedating agents, and sedation scores are used.
- Nurses determine the level of sedation at least 3 times per day. The adequacy of sedation in each patient is evaluated using a Richmond Agitation Sedation Scale (RASS). A RASS score of –2 to 0 is seen as adequate sedation.
- The goals of sedation are to reduce agitation, stress and fear; to reduce oxygen consumption (heart rate, blood pressure and minute volume are measured continuously); and to reduce physical resistance to– and fear of daily care and medical examination.
- The use of neuromuscular blockage is not recommended.
- Patient comfort is the primary goal. Level of pain is determined using scales such as Numeric Rating Scale (NRS), Visual Analogue Scale (VAS), Critical Care Pain Observation Tool (CCPOT) or Behavioral Pain Scale (BPS).

Ventilator associated pneumonia prevention

- If patients are expected to need ventilation for longer than 48 hours and/or are expected to stay in de ICU for longer than 72 hours, preventive measurements must be taken to prevent a ventilator associated pneumonia according to the local guidelines.

Fluid regimens

- A restrictive fluid strategy is recommended. A fluid balance targeted at normovolemia and a diuresis of ≥ 0.5 ml/kg/hour should be maintained with diuretics or by crystalloid infusions, that are preferred over colloid infusions.

Thrombosis prophylaxis

- Thrombosis prophylaxis is indicated for all patients who are not treated with anticoagulants, e.g. for therapeutic reasons or systemic prophylaxis because of an implanted device or extracorporeal circulation like for renal replacement therapy. Thrombosis prophylaxis will be given according to local guidelines.

Nutrition

- Enteral nutrition with a feeding gastric tube is preferred over intravenous feeding. If stomach retention occurs, a duodenal tube can be used if administration of prokinetic drugs is not sufficient, according to local guidelines. When optimal protein intake cannot be reached within 4 days, additional parenteral nutrition can be started.

Appendix 1: Recruitment maneuvers

Recruitment protocol:

1. The RM is performed by a qualified person, i.e., an intensivist of an intensive care doctor with sufficient experience;
2. Set PEEP at a minimum of 15 cm H_2O; if PEEP was not yet at 15 cm H_2O, it is increased in steps of 1 to 2 cm H_2O, wherein each steps last at least 10 seconds to see if the blood pressure remains acceptable. hemodynamic instability occurs; if PEEP is already > 15 cm H_2O, it is left unchanged;
3. Perform an inspiratory hold of 10 seconds by pressing the inspiratory hold button for 10 seconds; closely monitor the blood pressure, as if it drops the rescue maneuver is stopped to take measure to ensure hemodynamic stability (e.g., by raising the dose of vasoactive medication);
4. In successive steps, set the upper airway pressure 15 cm H_2O above PEEP, followed by an inspiratory hold of 10 seconds by pressing the inspiratory hold button for 10 seconds; closely monitor the blood pressure, as if it drops the rescue maneuver is stopped to take measure to ensure hemodynamic stability (e.g., by raising the dose of vasoactive medication);
5. This maneuver is repeated 3 times
6. At PEEP of 15 cm H_2O, the upper airway pressure is set in such a way that the tidal volumes again correspond to the ventilation settings of the randomization arm.

Appendix 2: Abbreviations

ARDS: Acute respiratory distress syndrome

ECMO: Extracorporeal membrane oxygenation

FiO_2: Fraction of inspired oxygen

I: E ratio: Inspiration to expiration ratio

ICU: Intensive care unit

MV: Mechanical ventilation

$PaCO_2$: Partial pressure of carbon dioxide

PaO_2: Partial pressure of oxygen

PBW: Predicted body weight

PEEP: Positive end-expiratory pressure

RASS: Richmond agitation sedation scale

RM: Recruitment maneuver

SBT: Spontaneous breathing trial

SpO_2: Oxygen saturation
