# Supplementary material for: Personalized mechanical ventilation guided by ultrasound in patients with acute respiratory distress syndrome (PEGASUS): study protocol for an international randomized clinical trial
Source: Trials. 2024 May 7;25:308. doi: 10.1186/s13063-024-08140-7 (PMC11077821; doi:10.1186/s13063-024-08140-7)
Supplement: Supplementary file 3 — Supplementary Material 3. [file 13063_2024_8140_MOESM3_ESM.pdf]

PEGASUS - version 196.11

Printed on 02-04-2024 17:51:11 by Jante Sinnige

1. Inclusion - Eligibility

Number	Question	Answers
	**INCLUSION CRITERIA**	
	Admitted to a participating ICU	
	Invasively ventilated	
	Fulfill the Berlin criteria for moderate or severe ARDS < 12 hours	
1.1	Does the patient meet all of the inclusion criteria?<br>*Exclude patient if field's value is equal to No with message: 'Patient cannot be included in the study. STOP CRF'*<br>*Field type*: Radiobutton<br>*Variable name*: incl<br>*Field required*: Required<br>*Option group name*: Yes/No	○ Yes<br>○ No
	**EXCLUSION CRITERIA**	
	Age under 18	
	Participation in another interventional study with conflicting endpoints	
	Conditions in which LUS is not feasible (e.g. subcutaneous emphysema, wounds)	
	Mechanical ventilation for longer than 7 consecutive days in the past 30 days	
	History of ARDS in the previous month	
	Body-mass index higher than 40 kg/m2	
	Intracranial hypertension	
	Broncho-pleural fistula	
	Chronic respiratory disease requiring long-term oxygen therapy or respiratory support	
	Pulmonary fibrosis with a vital capacity <50% (severe or very severe)	
	Patients who are moribound or facing end of life	
	Receiving or planned to receive ECMO	
	Patients who receive invasive ventilation in home setting due to a neurological disease	
	Previously randomized in this study	

	No informed consent	
1.2	Are there any exclusion criteria?<br>*Exclude patient if field's value is equal to Yes with message: 'Patient cannot be included in the study. STOP CRF'*<br>*Field type*: Radiobutton<br>*Variable name*: excl<br>*Field required*: Required<br>*Option group name*: Yes/No	◯ Yes<br>◯ No
	**ELIGIBILITY**	
1.3	Is the patient eligible for PEGASUS<br>*Notice shown if field's value is equal to Yes: 'Patient can be included in the study. Proceed to randomization.'*<br>*Field type*: Calculation<br>*Variable name*: Eligibility<br>*Field required*: Not required	*Template:* '##allowempty##'; if ({incl} == 1 && {excl} == 0) { 'Yes' } else { 'No' }

2. Inclusion - Randomization

Number	Question	Answers
2.1	Name of the person who performed the randomization (NOT patients name)<br>*Field type*: Textfield<br>*Variable name*: Name_rando<br>*Field required*: Required	
2.2	Age patient<br>*Exclude patient if field's value is smaller than 18 with message: 'CHECK EXCLUSION CRITERIA!'*<br>*Field type*: Numeric field<br>*Variable name*: Age_rando<br>*Field required*: Required<br>*Field min*: 0<br>*Field max*: 99<br>*Measurement Unit*: years	years
2.3	Gender patient<br>*Field type*: Radiobutton<br>*Variable name*: Gender_rando<br>*Field required*: Required<br>*Option group name*: Male_female	◯ Male<br>◯ Female
	**TO RANDOMIZE THE PATIENT, CLICK ON "NOT RANDOMIZED" ON THE FAR LEFT. THEN ON THE RANDOMIZE BUTTON**	
2.4	Randomization allocation<br>*Field type*: Randomization field<br>*Variable name*: randalloc<br>*Field required*: Not required	

2.5	Confirm randomization<br>*Field type*: Radiobutton<br>*Variable name*: Conf_rando<br>*Field required*: Required<br>*Option group name*: Randomization	◯ Personalized ventilation<br>◯ Standard Care

3. Inclusion - Informed consent

Number	Question	Answers
	**IF informed consent has NOT been obtained but the 72 hours after randomization has NOT been passed, please CONTINUE the CRF**	
3.1	Signed informed consent within 72 hours?<br>*Exclude patient if field's value is equal to No informed consent with message: 'Patient cannot be included in the study. STOP CRF'*<br>*Notice shown if field's value is equal to Patient died before consent could have been aquired: 'PATIENT REMAINS IN THE STUDY: CONTINUE CRF'*<br>*Field type*: Radiobutton<br>*Variable name*: inf_const<br>*Field required*: Required<br>*Option group name*: Consent	◯ No informed consent<br>◯ Informed consent from patient/ legal representatieve<br>◯ Patient died before consent could have been aquired
3.1.1	***If 'Signed informed consent within 72 hours?' is equal to 'Informed consent from patient/ legal representatieve' answer this question:***<br>Informed consent from<br>*Field type*: Checkbox<br>*Variable name*: IC_rep_or_pat<br>*Field required*: Required<br>*Option group name*: Legal or patient	☐ Legal representatieve<br>☐ Patient
3.1.1.1	***If 'Informed consent from' is equal to 'Legal representatieve' answer this question:***<br>Date of signed informed consent from legal representative<br>*Field type*: Date<br>*Variable name*: IC_rep_date<br>*Field required*: Required	[ ] [ ] [ ] *(dd-mm-yyyy)*
3.1.1.2	***If 'Informed consent from' is equal to 'Legal representatieve' answer this question:***<br>Time of signed informed consent from legal representative<br>*Field type*: Time<br>*Variable name*: IC_rep_time<br>*Field required*: Required	[ ] : [ ] *(hh:mm)*

3.1.1.3	***If 'Informed consent from' is equal to 'Patient' answer this question:***<br>Date of signed informed consent from patient<br>*Field type*: Date<br>*Variable name*: IC_pat_date<br>*Field required*: Required	[ ] [ ] [ ] *(dd-mm-yyyy)*
3.1.1.4	***If 'Informed consent from' is equal to 'Patient' answer this question:***<br>Time of signed informed consent from patient<br>*Field type*: Time<br>*Variable name*: IC_pat_time<br>*Field required*: Required	[ ]:[ ] *(hh:mm)*

4. Inclusion - Baseline

Number	Question	Answers
4.1	Height<br>*Warning shown if field's value is larger than or equal to 200: 'High value! Please control data input or make a comment.'*<br>*Warning shown if field's value is smaller than or equal to 130: 'Low value! Please control data input or make a comment.'*<br>Enter values that apply at the time of randomization or the first value before randomization.<br><br>*Field type*: Numeric field<br>*Variable name*: Height<br>*Field required*: Required<br>*Field min*: 120<br>*Field max*: 210<br>*Measurement Unit*: cm	[ ] cm
4.2	Weight<br>*Warning shown if field's value is larger than or equal to 140: 'High value! Please control data input or make a comment.'*<br>*Warning shown if field's value is smaller than or equal to 45: 'Low value! Please control data input or make a comment.'*<br>Enter values that apply at the time of randomization or the first value before randomization.<br><br>*Field type*: Numeric field<br>*Variable name*: Weight<br>*Field required*: Required<br>*Field min*: 35<br>*Field max*: 180<br>*Measurement Unit*: kg	[ ] kg

4.3	Days of ventilation before randomization<br>*Exclude patient if field's value is larger than 7 with message: 'CONTROL EXCLUSION CRITERIA!'*<br>*Warning shown if field's value is equal to 0: 'Input not possible, see (i) for more information.'*<br>Enter the value that applies at the time of randomization. Count every day that the patient is on the ventilator as a whole day. This number is always higher dan 0. Example: When a patiënt is intubated at 23:30 and included the next day on 9:00, you have to fill in 2 days.<br><br>*Field type*: Numeric field<br>*Variable name*: Day_ven<br>*Field required*: Required<br>*Field min*: 1<br>*Field max*: 14<br>*Measurement Unit*: days	[ ] days
4.4	Hours between ARDS diagnosis and randomization<br>*Exclude patient if field's value is larger than 12 with message: 'CONTROL INCLUSION CRITERIA!'*<br>Count the hours from ARDS diagnosis until randomization and round up to whole numbers. This value cannot be more than 12 hours (see exclusion criteria).<br><br>*Field type*: Numeric field<br>*Variable name*: Hours_ARDS<br>*Field required*: Required<br>*Field min*: 0<br>*Field max*: 24<br>*Measurement Unit*: Hours	[ ] Hours
4.5	Cause of ICU admission<br>Multiple reasons for the cause of ICU admission can be filled in.<br><br>*Field type*: Checkbox<br>*Variable name*: Cause_ICU_admiss<br>*Field required*: Required<br>*Option group name*: Cause ICU admission	☐ Sepsis<br>☐ Hemorrhagic shock<br>☐ Coma<br>☐ Acute respiratory failure<br>☐ Acute metabolic disorders<br>☐ Elective surgery<br>☐ Urgent surgery

4.6	Cause of ARDS<br>Multiple reasons for the cause of ARDS can be filled in. If there are multiple causes for the development of ARDS, please fill in at question 14.7.1 which cause probably had the most influence on the development of ARDS.<br><br>*Field type*: Checkbox<br>*Variable name*: Cause_ARDS<br>*Field required*: Required<br>*Option group name*: Cause ARDS	☐ Pneumonia<br>☐ Non-pulmonary sepsis<br>☐ Aspiration of gastric contens<br>☐ Major trauma<br>☐ Pulmonary contusion<br>☐ Pancreatitis<br>☐ Inhalation injury<br>☐ Severe burns<br>☐ Non-cardiogenic shock<br>☐ Drug overdose<br>☐ TRALI<br>☐ Pulmonary vasculitis<br>☐ Drowning
4.7.1	***If 'Calculation cause ARDS' is greater than '1' answer this question:***<br>Which cause had the greatest share in the development of ARDS<br>If there are multiple causes for the development of ARDS, which cause probably had the most influence on the development of ARDS<br><br>*Field type*: Radiobutton<br>*Variable name*: Spec_cause_ARDS<br>*Field required*: Required<br>*Option group name*: Cause ARDS	○ Pneumonia<br>○ Non-pulmonary sepsis<br>○ Aspiration of gastric contens<br>○ Major trauma<br>○ Pulmonary contusion<br>○ Pancreatitis<br>○ Inhalation injury<br>○ Severe burns<br>○ Non-cardiogenic shock<br>○ Drug overdose<br>○ TRALI<br>○ Pulmonary vasculitis<br>○ Drowning
4.7	Calculation cause ARDS<br>*Field type*: Calculation<br>*Variable name*: cal_cause_ards<br>*Field required*: Not required	*Template:* '##allowempty##'; if("{Cause_ARDS}" == "'NA'") { '0'; } else { var splitted = "{Cause_ARDS}".split(";"); splitted.length; }
4.8	Daily cumulative fluid balance since ICU admission<br>Cumulative fluid balance on the day of randomisation (ml/24h) since ICU admission. If the patient is admitted to the hospital on the day of randomization, take the cumulative fluid balance of the hours that the patient was in the hospital on this day.<br><br>*Field type*: Radiobutton<br>*Variable name*: Cum_fluid<br>*Field required*: Required<br>*Option group name*: Negative/positve	○ Negative<br>○ Positive

4.9	Daily cumulative fluid balance<br>*Warning shown if field's value is larger than or equal to 4000: 'High value! Please control data input or make a comment.'*<br>*Field type*: Numeric field<br>*Variable name*: Cum_fluid_ml<br>*Field required*: Required<br>*Field min*: 0<br>*Field max*: 30000<br>*Measurement Unit*: ml/24h	[ ] ml/24h
4.10	Clinical Frailty Scale<br>You can find extra information in the figure below.<br>*Field type*: Radiobutton<br>*Variable name*: Frailty_0<br>*Field required*: Required<br>*Option group name*: Fraily score	○ Very fit<br>○ Well<br>○ Managing Well<br>○ Vulnerable<br>○ Midly Frail<br>○ Moderately Frail<br>○ Severely Frail<br>○ Very Severely Frail<br>○ Terminally ill
4.11	Clinical Frailty Scale<br>*Field type*: Image<br>*Variable name*: image_frailty<br>*Field required*: Not required	
4.12	Apache II score<br>*Field type*: Numeric field<br>*Variable name*: Apache_II<br>*Field required*: Not required<br>*Field min*: 0<br>*Field max*: 71	[ ]
4.13	Apache IV score<br>*Field type*: Numeric field<br>*Variable name*: Apache_IV<br>*Field required*: Not required<br>*Field min*: 0<br>*Field max*: 286	[ ]
4.14	SAPS II score<br>*Field type*: Numeric field<br>*Variable name*: SAPS_II<br>*Field required*: Not required<br>*Field min*: 0<br>*Field max*: 163	[ ]
4.15	SAPS III score<br>*Field type*: Numeric field<br>*Variable name*: SAPS_III<br>*Field required*: Not required<br>*Field min*: 0<br>*Field max*: 217	[ ]

SOFA SCORE

4.16	PaO2/FiO2 (mmHg (kPa))<br>*Exclude patient if field's value is equal to ≥ 400 (53.3) with message: 'CONTROL INCLUSION CRITERIA!'*<br>*Exclude patient if field's value is equal to < 400 (53.3) with message: 'CONTROL INCLUSION CRITERIA!'*<br>*Exclude patient if field's value is equal to < 300 (40) with message: 'CONTROL INCLUSION CRITERIA!'*<br>Worst value of this day. If there is no value for this day please enter missing data.<br><br>*Field type*: Radiobutton<br>*Variable name*: SOFA_Resp_0<br>*Field required*: Required<br>*Option group name*: Respiration	○ ≥ 400 (53.3)<br>○ < 400 (53.3)<br>○ < 300 (40)<br>○ < 200 (26.7) with respiratory support<br>○ < 100 (13.3) with respiratory support
4.17	Platelets (x10^3/µL)<br>Worst value of this day. If there is no value for this day please enter missing data.<br><br>*Field type*: Radiobutton<br>*Variable name*: SOFA_Coag_0<br>*Field required*: Required<br>*Option group name*: Coagulation	○ ≥ 150<br>○ < 150<br>○ < 100<br>○ < 50<br>○ < 20
4.18	Bilirubin (µmol/L(mg/dL))<br>Worst value of this day. If there is no value for this day please enter missing data.<br><br>*Field type*: Radiobutton<br>*Variable name*: SOFA_Live_0<br>*Field required*: Required<br>*Option group name*: Liver	○ < 20 (< 1.2)<br>○ 20-32 (1.2-1.9)<br>○ 33-101 (2.0-5.9)<br>○ 102-204 (6.0-11.9)<br>○ > 204 (> 12.0)
4.19	Cardiovascular<br>Worst value of this day. If there is no value for this day please enter missing data.<br><br>*Field type*: Radiobutton<br>*Variable name*: SOFA_Card_0<br>*Field required*: Required<br>*Option group name*: Cardiovascular	○ MAP ≥ 70 mm/Hg without dobutamine, epinephrine or norepinephrine<br>○ MAP < 70 mm/Hg without dobutamine, epinephrine or norepinephrin<br>○ Dopamine < 5 µg/kg/min or dobutamine (any dose)<br>○ Dopamine > 5 µg/kg/min OR epinephrine ≤ 0.1 µg/kg/min OR norepinephrine ≤ 0.1 µg/kg/min<br>○ Dopamine > 15 µg/kg/min OR epinephrine > 0.1 µg/kg/min OR norepinephrine > 0.1 µg/kg/min
4.20	Glasgow coma scale<br>Only fill in the score when the patient is not sedated. Choose the worst value of this day. If there is no value for this day or the patient is sedated, please enter missing data.<br><br>*Field type*: Radiobutton<br>*Variable name*: SOFA_Nerv_0<br>*Field required*: Required<br>*Option group name*: Nervous	○ 15<br>○ 13-14<br>○ 10-12<br>○ 6-9<br>○ < 6

4.21	Creatinine (μmol/L) [or urine output (L/24h)]<br>Worst value of this day. If there is no value for this day please enter missing data.<br>*Field type*: Radiobutton<br>*Variable name*: SOFA_Kidn_0<br>*Field required*: Required<br>*Option group name*: Kidneys	◯ < 110 (< 1.2)<br>◯ 110-170 (1.2-1.9)<br>◯ 171-299 (2.0-3.4)<br>◯ 300-440 (3.5-4.9) [or < 500]<br>◯ > 440 (> 5.0) [or < 200]
4.22	SOFA score<br>*Field type*: Calculation<br>*Variable name*: SOFA_scor_0<br>*Field required*: Not required	*Template:* {SOFA_Resp_0}+{SOFA_Coag_0}+{SOFA_Live_0}+{SOFA_Card_0}+{SOFA_Nerv_0}+{SOFA_Kidn_0}
4.23	BMI<br>*Field type*: Calculation<br>*Variable name*: bmi<br>*Field required*: Not required	*Template:* bmi={Weight}/(({Height}/100)^2)
4.24	***If 'Gender patient' is equal to 'Female' answer this question:***<br>PBW female<br>*Field type*: Calculation<br>*Variable name*: PBW_female<br>*Field required*: Not required	*Template:* pdw=45.5+(0.91*{Height}-152.4)
4.25	***If 'Gender patient' is equal to 'Male' answer this question:***<br>PBW Male<br>*Field type*: Calculation<br>*Variable name*: PBW_Male<br>*Field required*: Not required	*Template:* pdw=50+(0.91*{Height}-152.4)

5. Inclusion - Comorbidities

Number	Question	Answers
5.1	Age (years)<br>*Field type*: Radiobutton<br>*Variable name*: cci_age<br>*Field required*: Required<br>*Option group name*: cci_age	◯ <50<br>◯ 50-59<br>◯ 60-69<br>◯ 70-79<br>◯ > 80
5.2	Diabetes Mellitus<br>End organ damage of any form (e.g. nephropathy, retinopathy, neuropathy)<br>*Field type*: Radiobutton<br>*Variable name*: cci_dm<br>*Field required*: Required<br>*Option group name*: cci_dm	◯ No Diabetes Mellitus<br>◯ Uncomplicated Diabetes Mellitus<br>◯ Diabetes Mellitus with end-organ damage

5.3	Liver disease<br>Severe = cirrhosis and portal hypertension with variceal bleeding history. Moderate = cirrhosis and portal hypertension but no variceal bleeding history. Mild = chronic hepatitis (or cirrhosis without portal hypertension).<br><br>*Field type*: Radiobutton<br>*Variable name*: cci_liver<br>*Field required*: Required<br>*Option group name*: liverdisease	◯ No<br>◯ Mild<br>◯ Moderate to severe
5.4	Active malignancy<br>If a patient is cured from a malignancy, enter NO.<br><br>*Field type*: Radiobutton<br>*Variable name*: cci_malig<br>*Field required*: Required<br>*Option group name*: malignancy	◯ None<br>◯ Any Leukemia, lymphoma or localized solid tumor<br>◯ Metastatic solid tumor
5.5	AIDS<br>Number of CD4 cells < 200 cells/mm3 /mm3 OR HIV positive with one or more opportunistic infections regardless of the CD4 count.<br><br>*Field type*: Radiobutton<br>*Variable name*: cci_aids<br>*Field required*: Required<br>*Option group name*: Yes/No Aids	◯ Yes<br>◯ No
5.6	Moderate to severe CKD<br>Severe = on dialysis, status post kidney transplant, uremia<br>Moderate = creatinine >3 mg/dL (0.27 mmol/L)<br><br>*Field type*: Radiobutton<br>*Variable name*: cci_ckd<br>*Field required*: Required<br>*Option group name*: Yes/No ckd	◯ Yes<br>◯ No
5.7	Congestive heart failure<br>Exertional or paroxysmal nocturnal dyspnea and has responded to digitalis, diuretics, or afterload reducing agents.<br><br>*Field type*: Radiobutton<br>*Variable name*: cci_chf<br>*Field required*: Required<br>*Option group name*: Yes/No	◯ Yes<br>◯ No
5.8	Myocardial infarction<br>History of definite or probable MI (EKG changes and/or enzyme changes)<br><br>*Field type*: Radiobutton<br>*Variable name*: cci_mi<br>*Field required*: Required<br>*Option group name*: Yes/No	◯ Yes<br>◯ No

5.9	COPD<br>Any form of COPD<br><br>*Field type*: Radiobutton<br>*Variable name*: cci_copd<br>*Field required*: Required<br>*Option group name*: Yes/No	◯ Yes<br>◯ No
5.10	Peripheral vascular disease<br>Intermittent claudication or past bypass for chronic arterial insufficiency, history of gangrene or acute arterial insufficiency, or untreated thoracic or abdominal aneurysm (≥6 cm)<br><br>*Field type*: Radiobutton<br>*Variable name*: cci_pvd<br>*Field required*: Required<br>*Option group name*: Yes/No	◯ Yes<br>◯ No
5.11	CVA or TIA<br>History of a cerebrovascular accident with minor or no residua and transient ischemic attacks.<br><br>*Field type*: Radiobutton<br>*Variable name*: cci_cva<br>*Field required*: Required<br>*Option group name*: Yes/No	◯ Yes<br>◯ No
5.12	Dementia<br>Chronic cognitive deficit.<br><br>*Field type*: Radiobutton<br>*Variable name*: cci_dement<br>*Field required*: Required<br>*Option group name*: Yes/No	◯ Yes<br>◯ No
5.13	Hemiplegia<br>Complete hemiplegia of the left or right body.<br><br>*Field type*: Radiobutton<br>*Variable name*: cci_hemiplegia<br>*Field required*: Required<br>*Option group name*: Yes/no hemiplegia	◯ Yes<br>◯ No
5.14	Connective tissue disease<br>Any connective tissue disease (e.g. Rheumatoid arthritis, Scleroderma, Granulomatosis with polyangiitis (GPA), Churg-Strauss syndrome, Lupus, Microscopic polyangiitis, Polymyositis/dermatomyositis, Marfan syndrome)<br><br>*Field type*: Radiobutton<br>*Variable name*: cci_ctd<br>*Field required*: Required<br>*Option group name*: Yes/No	◯ Yes<br>◯ No

5.15	Peptic ulcer disease<br>Any history of treatment for ulcer disease or history of ulcer bleeding.<br><br>*Field type*: Radiobutton<br>*Variable name*: cci_pud<br>*Field required*: Required<br>*Option group name*: Yes/No	◯ Yes<br>◯ No
5.16	Use of systemic steroids before randomization<br>Use of any systemic steroids before ARDS diagnosis (oral or i.v.)<br><br>*Field type*: Radiobutton<br>*Variable name*: Steroids<br>*Field required*: Required<br>*Option group name*: Yes/No	◯ Yes<br>◯ No
5.17	Charlson Comorbidity Index<br>*Field type*: Calculation<br>*Variable name*: cci_value<br>*Field required*: Not required	*Template:* {cci_age} + {cci_dm} + {cci_liver} + {cci_malig} + {cci_aids} + {cci_ckd} + {cci_chf} + {cci_mi} + {cci_copd} + {cci_pvd} + {cci_cva} + {cci_dement} + {cci_hemiplegia} + {cci_ctd} + {cci_pud}
5.17.1	***If 'Charlson Comorbidity Index' is not equal to '0' answer this question:***<br>10-year survival rate<br>*Field type*: Calculation<br>*Variable name*: cci_survival<br>*Field required*: Not required	*Template:* 100*(Math.pow(0.983, (Math.exp({cci_value}*0.9))));

6. Inclusion - Data during the lung ultrasound exam

Number	Question	Answers
6.1	Time of start LUS<br>*Field type*: Time<br>*Variable name*: time_LUS<br>*Field required*: Required	[ ]:[ ] *(hh:mm)*
	**SEDATION DATA**	
6.2	RASS score<br>+4 Combative +3 Very agitated +2 Agitated +1 Restless 0 Alert and calm -1 Drowsy -2 Light sedation -3 Moderate sedation -4 Deep sedation -5 Unarousable<br><br>*Field type*: Radiobutton<br>*Variable name*: Rass<br>*Field required*: Required<br>*Option group name*: RASS Score	◯ ≥ 0<br>◯ - 1<br>◯ - 2<br>◯ - 3<br>◯ - 4<br>◯ - 5
	**VENTILATION DATA**	

6.3	Mode of ventilation<br>*Field type*: Radiobutton<br>*Variable name*: Mode_ven<br>*Field required*: Required<br>*Option group name*: Mode ventilation	◯ Controlled<br>◯ Spontaneous<br>◯ Adaptive
6.3.1	***If 'Mode of ventilation' is equal to 'Adaptive' answer this question:***<br>Triggered or timed adaptive ventilation<br>*Field type*: Radiobutton<br>*Variable name*: Trig_timed<br>*Field required*: Required<br>*Option group name*: Triggered/Timed	◯ Triggered<br>◯ Timed
6.4	Tidal volume<br>*Warning shown if field's value is smaller than 100: 'Low value! Please control data input or make a comment.'*<br>*Warning shown if field's value is larger than 900: 'High value! Please control data input or make a comment.'*<br>*Field type*: Numeric field<br>*Variable name*: Tidal<br>*Field required*: Required<br>*Field min*: 50<br>*Field max*: 1200<br>*Measurement Unit*: ml	[ ] ml
6.5	Total respiratory rate<br>*Warning shown if field's value is smaller than 10: 'Low value! Please control data input or make a comment.'*<br>*Warning shown if field's value is larger than 35: 'High value! Please control data input or make a comment.'*<br>*Field type*: Numeric field<br>*Variable name*: TRR<br>*Field required*: Required<br>*Field min*: 4<br>*Field max*: 50<br>*Measurement Unit*: breaths per minut	[ ] breaths per minut
6.6	PEEP<br>*Warning shown if field's value is smaller than 5: 'Low value! Please control data input or make a comment.'*<br>*Warning shown if field's value is larger than 19: 'High value! Please control data input or make a comment.'*<br>*Field type*: Numeric field<br>*Variable name*: Peep<br>*Field required*: Required<br>*Field min*: 0<br>*Field max*: 25<br>*Measurement Unit*: cmH2O	[ ] cmH2O

6.3.2	***If 'Mode of ventilation' is not equal to 'Spontaneous' answer this question:***<br>Pplateau<br>*Warning shown if field's value is smaller than or equal to 7: 'Low value! Please control data input or make a comment.'*<br>*Warning shown if field's value is larger than or equal to 30: 'High value! Please control data input or make a comment.'*<br>*Field type*: Numeric field<br>*Variable name*: Pplat<br>*Field required*: Required<br>*Field min*: 5<br>*Field max*: 40<br>*Measurement Unit*: cmH2O	[ ] cmH2O
6.3.3	***If 'Mode of ventilation' is not equal to 'Spontaneous' answer this question:***<br>Pmax/ Ppeak<br>*Warning shown if field's value is smaller than 10: 'Low value! Please control data input or make a comment.'*<br>*Warning shown if field's value is larger than 29: 'High value! Please control data input or make a comment.'*<br>*Field type*: Numeric field<br>*Variable name*: Pmax<br>*Field required*: Required<br>*Field min*: 5<br>*Field max*: 50<br>*Measurement Unit*: cmH2O	[ ] cmH2O
6.3.4	***If 'Mode of ventilation' is equal to 'Spontaneous' answer this question:***<br>Pressure support<br>*Warning shown if field's value is smaller than 5: 'Low value! Please control data input or make a comment.'*<br>*Warning shown if field's value is larger than 19: 'High value! Please control data input or make a comment.'*<br>*Field type*: Numeric field<br>*Variable name*: Psup<br>*Field required*: Required<br>*Field min*: 0<br>*Field max*: 30<br>*Measurement Unit*: cmH2O	[ ] cmH2O
6.7	Unit used for EtCO2, PaO2 and PaCO2<br>*Field type*: Radiobutton<br>*Variable name*: mmhg_kpa<br>*Field required*: Required<br>*Option group name*: kPa/mmHg	○ kPa<br>○ mmHg

6.7.1	***If 'Unit used for EtCO2, PaO2 and PaCO2' is equal to 'kPa' answer this question:***<br>EtCO2<br>*Warning shown if field's value is smaller than 2: 'Low value! Please control data input or make a comment.'*<br>*Warning shown if field's value is larger than 8: 'High value! Please control data input or make a comment.'*<br>*Field type*: Numeric field<br>*Variable name*: EtCO2_kpa<br>*Field required*: Required<br>*Field min*: 1<br>*Field max*: 20<br>*Measurement Unit*: kPa	kPa
6.7.2	***If 'Unit used for EtCO2, PaO2 and PaCO2' is equal to 'mmHg' answer this question:***<br>EtCO2<br>*Warning shown if field's value is smaller than 15: 'Low value! Please control data input or make a comment.'*<br>*Warning shown if field's value is larger than 59: 'High value! Please control data input or make a comment.'*<br>*Field type*: Numeric field<br>*Variable name*: EtCO2_mmhg<br>*Field required*: Required<br>*Field min*: 7<br>*Field max*: 140<br>*Measurement Unit*: mmHg	mmHg
6.8	FiO2 (closest to AGB, in decimal, e.g. 0.35)<br>*Field type*: Numeric field<br>*Variable name*: FiO2<br>*Field required*: Required<br>*Field min*: 0.209999999999999999922284388<br>*Field max*: 1	
6.9	SpO2 (closest to AGB)<br>*Notice shown if field's value is smaller than 80: 'Low value! Please control data input or make a comment.'*<br>*Field type*: Numeric field<br>*Variable name*: SpO2<br>*Field required*: Required<br>*Field min*: 60<br>*Field max*: 100<br>*Measurement Unit*: %	%
	**ARTERIAL BLOOD GAS, closest to LUS exam but before randomization**	
6.10	Date and time of the ABG<br>Take the blood gas closest to the ventilation data but for randomization<br>*Field type*: Date & Time<br>*Variable name*: Date_time_ABG<br>*Field required*: Required	*(dd-mm-yyyy)*<br>: *(hh:mm)*

6.11	Arterial pH<br>*Warning shown if field's value is smaller than 7.0: 'Low value! Please control data input or make a comment.'*<br>*Warning shown if field's value is larger than 7.7: 'High value! Please control data input or make a comment.'*<br>*Field type*: Numeric field<br>*Variable name*: ArtpH<br>*Field required*: Required<br>*Field min*: 6.5<br>*Field max*: 8<br>*Measurement Unit*: pH	[ ] pH
6.12	Arterial bicarbonate<br>*Warning shown if field's value is smaller than 10: 'Low value! Please control data input or make a comment.'*<br>*Warning shown if field's value is larger than 39: 'High value! Please control data input or make a comment.'*<br>*Field type*: Numeric field<br>*Variable name*: Bic<br>*Field required*: Required<br>*Field min*: 2<br>*Field max*: 70<br>*Measurement Unit*: mmol/l	[ ] mmol/l
6.13	Arterial lactate<br>*Warning shown if field's value is larger than 9.9: 'High value! Please control data input or make a comment.'*<br>*Field type*: Numeric field<br>*Variable name*: Lac<br>*Field required*: Required<br>*Field min*: 0<br>*Field max*: 25<br>*Measurement Unit*: mmol/L	[ ] mmol/L
6.14	Arterial saturation<br>*Notice shown if field's value is smaller than 80: 'Low value! Please control data input or make a comment.'*<br>*Field type*: Numeric field<br>*Variable name*: Sat<br>*Field required*: Required<br>*Field min*: 60<br>*Field max*: 100<br>*Measurement Unit*: %	[ ] %

6.7.3 ***If 'Unit used for EtCO2, PaO2 and PaCO2' is equal to 'kPa' answer this question:***
Arterial PaO2
Warning shown if field's value is smaller than 7: 'Low value! Please control data input or make a comment.'
Warning shown if field's value is larger than 18: 'High value! Please control data input or make a comment.'
Field type: Numeric field
Variable name: PaO2_kpa
Field required: Required
Field min: 4
Field max: 60
Measurement Unit: kPa

[ ] kPa

6.7.4 ***If 'Unit used for EtCO2, PaO2 and PaCO2' is equal to 'mmHg' answer this question:***
Arterial PaO2
Warning shown if field's value is smaller than 50: 'Low value! Please control data input or make a comment.'
Warning shown if field's value is larger than 150: 'High value! Please control data input or make a comment.'
Field type: Numeric field
Variable name: PaO2_mmhg
Field required: Required
Field min: 28
Field max: 500
Measurement Unit: mmHg

[ ] mmHg

6.7.5 ***If 'Unit used for EtCO2, PaO2 and PaCO2' is equal to 'kPa' answer this question:***
Arterial PaCO2
Warning shown if field's value is smaller than 2: 'Low value! Please control data input or make a comment.'
Warning shown if field's value is larger than 8: 'High value! Please control data input or make a comment.'
Field type: Numeric field
Variable name: PaCO2_kpa
Field required: Required
Field min: 1
Field max: 20
Measurement Unit: kPa

[ ] kPa

6.7.6 ***If 'Unit used for EtCO2, PaO2 and PaCO2' is equal to 'mmHg' answer this question:***
Arterial PaCO2
Warning shown if field's value is smaller than 15: 'Low value! Please control data input or make a comment.'
Warning shown if field's value is larger than 59: 'High value! Please control data input or make a comment.'
Field type: Numeric field
Variable name: PaCO2_mmhg
Field required: Required
Field min: 7
Field max: 140
Measurement Unit: mmHg

[ ] mmHg

7. Inclusion - LungUltrasound exam

Number	Question	Answers
7.1	LUS exam performed by<br>*Field type*: Textfield<br>*Variable name*: LUS_performed<br>*Field required*: Required	
	RIGHT LUNG	
7.2	Anterior region 1<br>*Field type*: Radiobutton<br>*Variable name*: RA1<br>*Field required*: Required<br>*Option group name*: LUS exam score	◯ A-pattern<br>◯ B-pattern <50%<br>◯ B-pattern >50%<br>◯ Consolidation
7.3	Anterior region 2<br>*Field type*: Radiobutton<br>*Variable name*: RA2<br>*Field required*: Required<br>*Option group name*: LUS exam score	◯ A-pattern<br>◯ B-pattern <50%<br>◯ B-pattern >50%<br>◯ Consolidation
7.4	Lateral region 3<br>*Field type*: Radiobutton<br>*Variable name*: RL3<br>*Field required*: Required<br>*Option group name*: LUS exam score	◯ A-pattern<br>◯ B-pattern <50%<br>◯ B-pattern >50%<br>◯ Consolidation
7.5	Lateral region 4<br>*Field type*: Radiobutton<br>*Variable name*: RL4<br>*Field required*: Required<br>*Option group name*: LUS exam score	◯ A-pattern<br>◯ B-pattern <50%<br>◯ B-pattern >50%<br>◯ Consolidation
7.6	Posterior region 5<br>*Field type*: Radiobutton<br>*Variable name*: RP5<br>*Field required*: Required<br>*Option group name*: LUS exam score	◯ A-pattern<br>◯ B-pattern <50%<br>◯ B-pattern >50%<br>◯ Consolidation
7.7	Posterior region 6<br>*Field type*: Radiobutton<br>*Variable name*: RP6<br>*Field required*: Required<br>*Option group name*: LUS exam posterior	◯ A-pattern or B-pattern < 50%<br>◯ B-pattern > 50%<br>◯ Consolidation
	LEFT LUNG	

7.8	Anterior region 1<br>*Field type*: Radiobutton<br>*Variable name*: LA1<br>*Field required*: Required<br>*Option group name*: LUS exam score	◯ A-pattern<br>◯ B-pattern <50%<br>◯ B-pattern >50%<br>◯ Consolidation
7.9	Anterior region 2<br>*Field type*: Radiobutton<br>*Variable name*: LA2<br>*Field required*: Required<br>*Option group name*: LUS exam score	◯ A-pattern<br>◯ B-pattern <50%<br>◯ B-pattern >50%<br>◯ Consolidation
7.10	Lateral region 3<br>*Field type*: Radiobutton<br>*Variable name*: LL3<br>*Field required*: Required<br>*Option group name*: LUS exam score	◯ A-pattern<br>◯ B-pattern <50%<br>◯ B-pattern >50%<br>◯ Consolidation
7.11	Lateral region 4<br>*Field type*: Radiobutton<br>*Variable name*: LL4<br>*Field required*: Required<br>*Option group name*: LUS exam score	◯ A-pattern<br>◯ B-pattern <50%<br>◯ B-pattern >50%<br>◯ Consolidation
7.12	Posterior region 5<br>*Field type*: Radiobutton<br>*Variable name*: LP5<br>*Field required*: Required<br>*Option group name*: LUS exam score	◯ A-pattern<br>◯ B-pattern <50%<br>◯ B-pattern >50%<br>◯ Consolidation
7.13	Posterior region 6<br>*Field type*: Radiobutton<br>*Variable name*: LP6<br>*Field required*: Required<br>*Option group name*: LUS exam posterior	◯ A-pattern or B-pattern < 50%<br>◯ B-pattern > 50%<br>◯ Consolidation
	**If there are LUS exam regions missing, use the flowchart below to define the ARDS fenotype**	
7.14	Flowchart Ultrasound<br>Missing LUS images are complemented by the mean LUS aeration score of the other available LUS images in the concerning region (anterior, lateral, or posterior region).<br>*Field type*: Image<br>*Variable name*: Flow_US<br>*Field required*: Not required	
7.15	Result LUS exam<br>*Field type*: Calculation<br>*Variable name*: Result_LUS<br>*Field required*: Not required	*Template:* if ({cal_anterior} >= 2) { 'Non-Focal' } else if ({cal_lateral} > {cal_posterior}){ 'Non-Focal' } else { 'Focal' }

7.16	Confirm result LUS exam<br>*Field type*: Radiobutton<br>*Variable name*: Confirm_LUS<br>*Field required*: Required<br>*Option group name*: LUS exam	◯ Focal<br>◯ Non-focal
7.17	Calculation anterior<br>*Field type*: Calculation<br>*Variable name*: cal_anterior<br>*Field required*: Not required	*Template:* {RA1} + {RA2} + {LA1} + {LA2}
7.18	Calculation lateral<br>*Field type*: Calculation<br>*Variable name*: cal_lateral<br>*Field required*: Not required	*Template:* {RL3}+{RL4}+{LL3}+{LL4}
7.19	Calculation posterior<br>*Field type*: Calculation<br>*Variable name*: cal_posterior<br>*Field required*: Not required	*Template:* {RP5}+{RP6}+{LP5}+{LP6}
	CT-THORAX	
7.20	Is there a CT scan of the thorax?<br>The CT-thorax must be performed before randomization but no longer than 24 hours before randomization.<br><br>*Field type*: Radiobutton<br>*Variable name*: CT_availble<br>*Field required*: Required<br>*Option group name*: Yes/No	◯ Yes<br>◯ No
7.20.1	***If 'Is there a CT scan of the thorax?' is equal to 'Yes' answer this question:***<br>Result CT-Thorax<br>Focal morphology is defined as isolated consolidations with an infero-dorsal dominance. Non-focal morphology is defined as presence of diffuse or patchy opacifications, with or without dorsal consolidations.<br><br>*Field type*: Radiobutton<br>*Variable name*: CT_result<br>*Field required*: Required<br>*Option group name*: LUS exam	◯ Focal<br>◯ Non-focal
	**The result from the CT-Thorax is different than the result from the LUS. Please control the input and if it is correct, please contact the coordinating team.**	

7.20.2	***If 'Is there a CT scan of the thorax?' is equal to 'Yes' answer this question:***<br>What was the PEEP level during the CT scan?<br>If the patient was not intubated, please fill in a zero (0).<br><br>*Field type*: Numeric field<br>*Variable name*: PEEP_CT<br>*Field required*: Required<br>*Field min*: 0<br>*Field max*: 20	
7.21	Control phenotype<br>*Field type*: Calculation<br>*Variable name*: Control_phenotype<br>*Field required*: Not required	*Template:* {Confirm_LUS} - {CT_result}

8. Inclusion - Ventilation data after randomization

Number	Question	Answers
	**VENTILATION DATA, two hours after randomization**	
8.1	RASS score<br>+4 Combative +3 Very agitated +2 Agitated +1 Restless 0 Alert and calm -1 Drowsy -2 Light sedation -3 Moderate sedation -4 Deep sedation -5 Unarousable<br><br>*Field type*: Radiobutton<br>*Variable name*: Rass_8<br>*Field required*: Required<br>*Option group name*: RASS Score	○ ≥ 0<br>○ - 1<br>○ - 2<br>○ - 3<br>○ - 4<br>○ - 5
8.2	Mode of ventilation<br>*Field type*: Radiobutton<br>*Variable name*: Mode_ven_8<br>*Field required*: Required<br>*Option group name*: Mode ventilation	○ Controlled<br>○ Spontaneous<br>○ Adaptive
8.2.1	***If 'Mode of ventilation' is equal to 'Adaptive' answer this question:***<br>Triggered or timed adaptive ventilation<br>*Field type*: Radiobutton<br>*Variable name*: Trig_timed_8<br>*Field required*: Required<br>*Option group name*: Triggered/Timed	○ Triggered<br>○ Timed

8.3	Tidal volume<br>*Warning shown if field's value is smaller than 100: 'Low value! Please control data input or make a comment.'*<br>*Warning shown if field's value is larger than 900: 'High value! Please control data input or make a comment.'*<br>*Field type*: Numeric field<br>*Variable name*: Tidal_8<br>*Field required*: Required<br>*Field min*: 50<br>*Field max*: 1200<br>*Measurement Unit*: ml	ml
8.4	Total respiratory rate<br>*Warning shown if field's value is smaller than 10: 'Low value! Please control data input or make a comment.'*<br>*Warning shown if field's value is larger than 35: 'High value! Please control data input or make a comment.'*<br>*Field type*: Numeric field<br>*Variable name*: TRR_8<br>*Field required*: Required<br>*Field min*: 4<br>*Field max*: 50<br>*Measurement Unit*: breaths per minut	breaths per minut
8.5	PEEP<br>*Warning shown if field's value is smaller than 5: 'Low value! Please control data input or make a comment.'*<br>*Warning shown if field's value is larger than 19: 'High value! Please control data input or make a comment.'*<br>*Field type*: Numeric field<br>*Variable name*: Peep_8<br>*Field required*: Required<br>*Field min*: 0<br>*Field max*: 25<br>*Measurement Unit*: cmH2O	cmH2O
8.2.2	***If 'Mode of ventilation' is not equal to 'Spontaneous' answer this question:***<br>Pplateau<br>*Warning shown if field's value is smaller than or equal to 7: 'Low value! Please control data input or make a comment.'*<br>*Warning shown if field's value is larger than or equal to 30: 'High value! Please control data input or make a comment.'*<br>*Field type*: Numeric field<br>*Variable name*: Pplat_8<br>*Field required*: Required<br>*Field min*: 5<br>*Field max*: 40<br>*Measurement Unit*: cmH2O	cmH2O

8.2.3	***If 'Mode of ventilation' is not equal to 'Spontaneous' answer this question:***<br>Pmax/ Ppeak<br>*Warning shown if field's value is smaller than 10: 'Low value! Please control data input or make a comment.'*<br>*Warning shown if field's value is larger than 29: 'High value! Please control data input or make a comment.'*<br>*Field type*: Numeric field<br>*Variable name*: Pmax_8<br>*Field required*: Required<br>*Field min*: 5<br>*Field max*: 50<br>*Measurement Unit*: cmH2O	[ ] cmH2O
8.2.4	***If 'Mode of ventilation' is equal to 'Spontaneous' answer this question:***<br>Pressure support<br>*Warning shown if field's value is smaller than 5: 'Low value! Please control data input or make a comment.'*<br>*Warning shown if field's value is larger than 19: 'High value! Please control data input or make a comment.'*<br>*Field type*: Numeric field<br>*Variable name*: Psup_8<br>*Field required*: Required<br>*Field min*: 0<br>*Field max*: 30<br>*Measurement Unit*: cmH2O	[ ] cmH2O
8.6	Unit used for EtCO2, PaO2 and PaCO2<br>*Field type*: Radiobutton<br>*Variable name*: mmhg_kpa_8<br>*Field required*: Required<br>*Option group name*: kPa/mmHg	○ kPa<br>○ mmHg
8.6.1	***If 'Unit used for EtCO2, PaO2 and PaCO2' is equal to 'kPa' answer this question:***<br>EtCO2<br>*Warning shown if field's value is smaller than 2: 'Low value! Please control data input or make a comment.'*<br>*Warning shown if field's value is larger than 8: 'High value! Please control data input or make a comment.'*<br>*Field type*: Numeric field<br>*Variable name*: EtCO2_kpa_8<br>*Field required*: Required<br>*Field min*: 1<br>*Field max*: 20<br>*Measurement Unit*: kPa	[ ] kPa

8.6.2 ***If 'Unit used for EtCO2, PaO2 and PaCO2' is equal to 'mmHg' answer this question:***
EtCO2
Warning shown if field's value is smaller than 15: 'Low value! Please control data input or make a comment.'
Warning shown if field's value is larger than 59: 'High value! Please control data input or make a comment.'
Field type: Numeric field
Variable name: EtCO2_mmhg_8
Field required: Required
Field min: 7
Field max: 140
Measurement Unit: mmHg

[ ] mmHg

8.7 FiO2 (closest to AGB, in decimal, e.g. 0.35)
Field type: Numeric field
Variable name: FiO2_8
Field required: Required
Field min: 0.2099999999999999922284388
Field max: 1

[ ]

8.8 SpO2 (closest to AGB)
Notice shown if field's value is smaller than 80: 'Low value! Please control data input or make a comment.'
Field type: Numeric field
Variable name: SpO2_8
Field required: Required
Field min: 60
Field max: 100
Measurement Unit: %

[ ] %

ARTERIAL BLOOD GAS, closest to ventilation data but after randomization

8.9 Date and time of the ABG
Take the blood gas closest to the ventilation data but after randomization

Field type: Date & Time
Variable name: Date_time_ABG_1
Field required: Required

[ ] [ ] [ ] *(dd-mm-yyyy)*
[ ] : [ ] *(hh:mm)*

8.10 Arterial pH
Warning shown if field's value is smaller than 7.0: 'Low value! Please control data input or make a comment.'
Warning shown if field's value is larger than 7.7: 'High value! Please control data input or make a comment.'
Field type: Numeric field
Variable name: ArtpH_8
Field required: Required
Field min: 6.5
Field max: 8
Measurement Unit: pH

[ ] pH

8.11	Arterial bicarbonate<br>*Warning shown if field's value is smaller than 10: 'Low value! Please control data input or make a comment.'*<br>*Warning shown if field's value is larger than 39: 'High value! Please control data input or make a comment.'*<br>*Field type*: Numeric field<br>*Variable name*: Bic_8<br>*Field required*: Required<br>*Field min*: 2<br>*Field max*: 70<br>*Measurement Unit*: mmol/l	mmol/l
8.12	Arterial lactate<br>*Warning shown if field's value is larger than 9.9: 'High value! Please control data input or make a comment.'*<br>*Field type*: Numeric field<br>*Variable name*: Lac_8<br>*Field required*: Required<br>*Field min*: 0<br>*Field max*: 25<br>*Measurement Unit*: mmol/L	mmol/L
8.13	Arterial saturation<br>*Notice shown if field's value is smaller than 80: 'Low value! Please control data input or make a comment.'*<br>*Field type*: Numeric field<br>*Variable name*: Sat_8<br>*Field required*: Required<br>*Field min*: 60<br>*Field max*: 100<br>*Measurement Unit*: %	%
8.6.3	***If 'Unit used for EtCO2, PaO2 and PaCO2' is equal to 'kPa' answer this question:***<br>Arterial PaO2<br>*Warning shown if field's value is smaller than 7: 'Low value! Please control data input or make a comment.'*<br>*Warning shown if field's value is larger than 18: 'High value! Please control data input or make a comment.'*<br>*Field type*: Numeric field<br>*Variable name*: PaO2_kpa_8<br>*Field required*: Required<br>*Field min*: 4<br>*Field max*: 60<br>*Measurement Unit*: kPa	kPa

8.6.4	***If 'Unit used for EtCO2, PaO2 and PaCO2' is equal to 'mmHg' answer this question:***<br>Arterial PaO2<br>*Warning shown if field's value is smaller than 50: 'Low value! Please control data input or make a comment.'*<br>*Warning shown if field's value is larger than 150: 'High value! Please control data input or make a comment.'*<br>*Field type*: Numeric field<br>*Variable name*: PaO2_mmhg_8<br>*Field required*: Required<br>*Field min*: 28<br>*Field max*: 500<br>*Measurement Unit*: mmHg	mmHg
8.6.5	***If 'Unit used for EtCO2, PaO2 and PaCO2' is equal to 'kPa' answer this question:***<br>Arterial PaCO2<br>*Warning shown if field's value is smaller than 2: 'Low value! Please control data input or make a comment.'*<br>*Warning shown if field's value is larger than 8: 'High value! Please control data input or make a comment.'*<br>*Field type*: Numeric field<br>*Variable name*: PaCO2_kpa_8<br>*Field required*: Required<br>*Field min*: 1<br>*Field max*: 20<br>*Measurement Unit*: kPa	kPa
8.6.6	***If 'Unit used for EtCO2, PaO2 and PaCO2' is equal to 'mmHg' answer this question:***<br>Arterial PaCO2<br>*Warning shown if field's value is smaller than 15: 'Low value! Please control data input or make a comment.'*<br>*Warning shown if field's value is larger than 59: 'High value! Please control data input or make a comment.'*<br>*Field type*: Numeric field<br>*Variable name*: PaCO2_mmhg_8<br>*Field required*: Required<br>*Field min*: 7<br>*Field max*: 140<br>*Measurement Unit*: mmHg	mmHg
	**INTERVENTION DATA, on the day of inclusion after randomization**	
8.14	Recruitment maneuvre<br>*Field type*: Radiobutton<br>*Variable name*: Recruit_8<br>*Field required*: Required<br>*Option group name*: Yes/No	○ Yes<br>○ No

8.14.1	***If 'Recruitment maneuvre' is equal to 'Yes' answer this question:***<br>Amount of recruitment maneuvres<br>*Warning shown if field's value is larger than 9: 'High value! Please control data input or make a comment'*<br>*Field type*: Numeric field<br>*Variable name*: Recruit_times_8<br>*Field required*: Required<br>*Field min*: 0<br>*Field max*: 20<br>*Measurement Unit*: times	[ ] times
8.15	Prone positioning<br>*Field type*: Radiobutton<br>*Variable name*: Prone_8<br>*Field required*: Required<br>*Option group name*: Yes/No	○ Yes<br>○ No
8.15.1	***If 'Prone positioning' is equal to 'Yes' answer this question:***<br>Duration of prone posistioning<br>*Field type*: Numeric field<br>*Variable name*: Prone_hours_8<br>*Field required*: Required<br>*Field min*: 0<br>*Field max*: 24<br>*Measurement Unit*: hours	[ ] hours

9. Daily data - Day 1

Number	Question	Answers
9.1	Date of study day<br>*Field type*: Calculation<br>*Variable name*: date_1<br>*Field required*: Not required	*Template:* var randomization= moment('{castorRandomizedDateAndTime}', 'DD-MM-YYYY'); var newDate = randomization.add(1, 'days'); newDate.format('DD-MM-YYYY');
9.2	Is the patient hospitalized during any time of the day?<br>*Notice shown if field's value is equal to No: 'DAILY DATA COMPLETE, GO TO FOLLOW UP.'*<br>*Field type*: Radiobutton<br>*Variable name*: Hospital_patient_1<br>*Field required*: Required<br>*Option group name*: Yes/No	○ Yes<br>○ No
9.2.1	***If 'Is the patient hospitalized during any time of the day?' is equal to 'Yes' answer this question:***<br>Location of the patient<br>At 6:00 AM<br>*Field type*: Radiobutton<br>*Variable name*: Loc_ICUhosp_1<br>*Field required*: Required<br>*Option group name*: ICU/hospital	○ Intensive Care Unit<br>○ Hospital ward

9.2.1.1	***If 'Location of the patient' is equal to 'Hospital ward' answer this question:***<br>Type of respiratory support<br>At 6:00 AM<br><br>*Field type*: Radiobutton<br>*Variable name*: Resp_hospsupp_1<br>*Field required*: Required<br>*Option group name*: resp_supp_hosp	◯ No oxygen therapy<br>◯ Oxygen by mask or nasal prongs<br>◯ High Flow Nasal Oxygen (HFNO) therapy<br>◯ Non-invasive ventilation (NIV)
9.2.1.2	***If 'Location of the patient' is equal to 'Intensive Care Unit' answer this question:***<br>Type of respiratory support<br>At 6:00 AM<br><br>*Field type*: Radiobutton<br>*Variable name*: Resp_supp_1<br>*Field required*: Required<br>*Option group name*: Respiratory support	◯ No oxygen therapy<br>◯ Oxygen by maks or nasal prongs<br>◯ High Flow Nasal Oxygen (HFNO) therapy<br>◯ Non-invasive ventilation (NIV)<br>◯ Mechanical ventilation
9.2.1.3	***If 'Location of the patient' is equal to 'Intensive Care Unit' answer this question:***<br>Daily cumulative fluid balance<br>*Field type*: Radiobutton<br>*Variable name*: Cum_fluid_1<br>*Field required*: Required<br>*Option group name*: Negative/positve	◯ Negative<br>◯ Positive
9.2.1.4	***If 'Location of the patient' is equal to 'Intensive Care Unit' answer this question:***<br>Daily cumulative fluid balance<br>*Warning shown if field's value is larger than or equal to 4000: 'High value! Please control data input or make a comment.'*<br>From 0:00 until 0:00<br><br>*Field type*: Numeric field<br>*Variable name*: Cum_fluid_ml_1<br>*Field required*: Required<br>*Field min*: 0<br>*Field max*: 30000<br>*Measurement Unit*: ml	[ ] ml
	**SOFA SCORE**	
9.2.1.6	***If 'Location of the patient' is equal to 'Intensive Care Unit' answer this question:***<br>PaO2/FiO2 (mmHg (kPa))<br>Worst value of this day. If there is no value for this day please enter missing data.<br><br>*Field type*: Radiobutton<br>*Variable name*: SOFA_Resp_1<br>*Field required*: Required<br>*Option group name*: Respiration	◯ ≥ 400 (53.3)<br>◯ < 400 (53.3)<br>◯ < 300 (40)<br>◯ < 200 (26.7) with respiratory support<br>◯ < 100 (13.3) with respiratory support

9.2.1.7	***If 'Location of the patient' is equal to 'Intensive Care Unit' answer this question:***<br>Platelets (x10^3/µL)<br>Worst value of this day. If there is no value for this day please enter missing data.<br><br>*Field type*: Radiobutton<br>*Variable name*: SOFA_Coag_1<br>*Field required*: Required<br>*Option group name*: Coagulation	◯ ≥ 150<br>◯ < 150<br>◯ < 100<br>◯ < 50<br>◯ < 20
9.2.1.8	***If 'Location of the patient' is equal to 'Intensive Care Unit' answer this question:***<br>Bilirubin (µmol/L(mg/dL))<br>Worst value of this day. If there is no value for this day please enter missing data.<br><br>*Field type*: Radiobutton<br>*Variable name*: SOFA_Live_1<br>*Field required*: Required<br>*Option group name*: Liver	◯ < 20 (< 1.2)<br>◯ 20-32 (1.2-1.9)<br>◯ 33-101 (2.0-5.9)<br>◯ 102-204 (6.0-11.9)<br>◯ > 204 (> 12.0)
9.2.1.9	***If 'Location of the patient' is equal to 'Intensive Care Unit' answer this question:***<br>Cardiovascular<br>Worst value of this day. If there is no value for this day please enter missing data.<br><br>*Field type*: Radiobutton<br>*Variable name*: SOFA_Card_1<br>*Field required*: Required<br>*Option group name*: Cardiovascular	◯ MAP ≥ 70 mm/Hg without dobutamine, epinephrine or norepinephrine<br>◯ MAP < 70 mm/Hg without dobutamine, epinephrine or norepinephrin<br>◯ Dopamine < 5 µg/kg/min or dobutamine (any dose)<br>◯ Dopamine > 5 µg/kg/min OR epinephrine ≤ 0.1 µg/kg/min OR norepinephrine ≤ 0.1 µg/kg/min<br>◯ Dopamine > 15 µg/kg/min OR epinephrine > 0.1 µg/kg/min OR norepinephrine > 0.1 µg/kg/min
9.2.1.10	***If 'Location of the patient' is equal to 'Intensive Care Unit' answer this question:***<br>Glasgow coma scale<br>Only fill in the score when the patient is not sedated. Choose the worst value of this day. If there is no value for this day or the patient is sedated, please enter missing data.<br><br>*Field type*: Radiobutton<br>*Variable name*: SOFA_Nerv_1<br>*Field required*: Required<br>*Option group name*: Nervous	◯ 15<br>◯ 13-14<br>◯ 10-12<br>◯ 6-9<br>◯ < 6
9.2.1.11	***If 'Location of the patient' is equal to 'Intensive Care Unit' answer this question:***<br>Creatinine [or urine output]<br>Worst value of this day. If there is no value for this day please enter missing data.<br><br>*Field type*: Radiobutton<br>*Variable name*: SOFA_Kidn_1<br>*Field required*: Required<br>*Option group name*: Kidneys	◯ < 110 (< 1.2)<br>◯ 110-170 (1.2-1.9)<br>◯ 171-299 (2.0-3.4)<br>◯ 300-440 (3.5-4.9) [or < 500]<br>◯ > 440 (> 5.0) [or < 200]

9.2.1.12	***If 'Location of the patient' is equal to 'Intensive Care Unit' answer this question:***<br>SOFA score<br>*Field type*: Calculation<br>*Variable name*: SOFA_scor_1<br>*Field required*: Not required	*Template:* {SOFA_Resp_1}+{SOFA_Coag_1}+{SOFA_Live_1}+{SOFA_Card_1}+{SOFA_Nerv_1}+{SOFA_Kidn_1}
	**EVENTS**	
9.2.1.14	***If 'Location of the patient' is equal to 'Intensive Care Unit' answer this question:***<br>Did an event occur?<br>• Renal Replacement Therapy during any time of the day.<br>• Placement of a tracheostomy during any time of the day<br>• Use of inhaled vasodilators during any time of the day<br>• Use of airway pressure release ventlation during any time of the day<br>• Use of ECMO during any time of the day<br><br>*Field type*: Checkbox<br>*Variable name*: Event_1<br>*Field required*: Required<br>*Option group name*: Events	☐ None<br>☐ Use of renal replacement therapy<br>☐ Placement of a tracheostomy<br>☐ Use of inhaled vasodilators<br>☐ Use of airway pressure release ventilation<br>☐ Use of ECMO<br>☐ Continuous infusion of neuromuscular blocking agents
9.2.1.15	***If 'Location of the patient' is equal to 'Intensive Care Unit' answer this question:***<br>Calculation for incorrect answer event<br>*Field type*: Calculation<br>*Variable name*: Event_calc_incorrect_1<br>*Field required*: Not required	*Template:* '##allowempty##' var splitted = "{Event_1}".split(';'); if (splitted.indexOf("0") > -1 && (splitted.indexOf("1") > -1 || splitted.indexOf("2") > -1 || splitted.indexOf("3") > -1 || splitted.indexOf("4") > -1 || splitted.indexOf("5") > -1 || splitted.indexOf("6") > -1)) { '1'; } else { '0'; };
	Combination **NOT** possible! **NONE** cannot be chosen when other options are selected!	
	**COMPLICATIONS**	
9.2.1.17	***If 'Location of the patient' is equal to 'Intensive Care Unit' answer this question:***<br>Did a complication occur?<br>Only tick the checkbox at the day of diagnosis.<br><br>• Pneumothorax: Air in the pleural cavity developed after randomization confirmed by a radiologist on a CT-thorax or chest radiograph for which a drain has been placed.<br>• Ventilator Associated Pneumonia: Clinical Pulmonary Infection Score (CPIS) > 5 with an infiltration on CXR and developed after intubation.<br><br>*Field type*: Checkbox<br>*Variable name*: Compl_1<br>*Field required*: Required<br>*Option group name*: Complications	☐ None<br>☐ Diagnosis of pneumothorax<br>☐ Diagnosis of Ventilator Associated Pneumonia

9.2.1.18	***If 'Location of the patient' is equal to 'Intensive Care Unit' answer this question:***<br>Calculation for incorrect answer complications<br>*Field type*: Calculation<br>*Variable name*: Compl_calc_incorrect_1<br>*Field required*: Not required	*Template:* '##allowempty##' var splitted = "{Compl_1}".split(';'); if (splitted.indexOf("0") > -1 && (splitted.indexOf("1") > -1 || splitted.indexOf("2") > -1 || splitted.indexOf("3") > -1 || splitted.indexOf("4") > -1 || splitted.indexOf("5") > -1 || splitted.indexOf("6") > -1)) { '1'; } else { '0'; };
	Combination **NOT** possible! **NONE** cannot be chosen when other options are selected!	
	**VENTILATION DATA AT 6:00 AM**	
9.2.1.2.2	***If 'Type of respiratory support' is equal to 'Mechanical ventilation' answer this question:***<br>RASS score<br>+4 Combative +3 Very agitated +2 Agitated +1 Restless 0 Alert and calm -1 Drowsy -2 Light sedation -3 Moderate sedation -4 Deep sedation -5 Unarousable<br>*Field type*: Radiobutton<br>*Variable name*: Rass_1<br>*Field required*: Required<br>*Option group name*: RASS Score	◯ ≥ 0<br>◯ - 1<br>◯ - 2<br>◯ - 3<br>◯ - 4<br>◯ - 5
9.2.1.2.3	***If 'Type of respiratory support' is equal to 'Mechanical ventilation' answer this question:***<br>Mode of ventilation<br>*Field type*: Radiobutton<br>*Variable name*: Mode_ven_1<br>*Field required*: Required<br>*Option group name*: Mode ventilation	◯ Controlled<br>◯ Spontaneous<br>◯ Adaptive
9.2.1.2.3.1	***If 'Mode of ventilation' is equal to 'Adaptive' answer this question:***<br>Triggered or timed adaptive ventilation<br>*Field type*: Radiobutton<br>*Variable name*: Trig_timed_1<br>*Field required*: Required<br>*Option group name*: Triggered/Timed	◯ Triggered<br>◯ Timed
9.2.1.2.4	***If 'Type of respiratory support' is equal to 'Mechanical ventilation' answer this question:***<br>Tidal volume<br>*Warning shown if field's value is smaller than 100: 'Low value! Please control data input or make a comment.'*<br>*Warning shown if field's value is larger than 900: 'High value! Please control data input or make a comment.'*<br>*Field type*: Numeric field<br>*Variable name*: Tidal_1<br>*Field required*: Required<br>*Field min*: 50<br>*Field max*: 1200<br>*Measurement Unit*: ml	[ ] ml

9.2.1.2.5	***If 'Type of respiratory support' is equal to 'Mechanical ventilation' answer this question:***<br>Total respiratory rate<br>*Warning shown if field's value is smaller than 10: 'Low value! Please control data input or make a comment.'*<br>*Warning shown if field's value is larger than 35: 'High value! Please control data input or make a comment.'*<br>*Field type*: Numeric field<br>*Variable name*: TRR_1<br>*Field required*: Required<br>*Field min*: 4<br>*Field max*: 50<br>*Measurement Unit*: breaths per minut	breaths per minut
9.2.1.2.6	***If 'Type of respiratory support' is equal to 'Mechanical ventilation' answer this question:***<br>PEEP<br>*Warning shown if field's value is smaller than 5: 'Low value! Please control data input or make a comment.'*<br>*Warning shown if field's value is larger than 19: 'High value! Please control data input or make a comment.'*<br>*Field type*: Numeric field<br>*Variable name*: Peep_1<br>*Field required*: Required<br>*Field min*: 0<br>*Field max*: 25<br>*Measurement Unit*: cmH2O	cmH2O
9.2.1.2.3.2	***If 'Mode of ventilation' is not equal to 'Spontaneous' answer this question:***<br>Pplateau<br>*Warning shown if field's value is smaller than or equal to 7: 'Low value! Please control data input or make a comment.'*<br>*Warning shown if field's value is larger than or equal to 30: 'High value! Please control data input or make a comment.'*<br>*Field type*: Numeric field<br>*Variable name*: Pplat_1<br>*Field required*: Required<br>*Field min*: 5<br>*Field max*: 40<br>*Measurement Unit*: cmH2O	cmH2O
9.2.1.2.3.3	***If 'Mode of ventilation' is not equal to 'Spontaneous' answer this question:***<br>Pmax/ Ppeak<br>*Warning shown if field's value is smaller than 10: 'Low value! Please control data input or make a comment.'*<br>*Warning shown if field's value is larger than 29: 'High value! Please control data input or make a comment.'*<br>*Field type*: Numeric field<br>*Variable name*: Pmax_1<br>*Field required*: Required<br>*Field min*: 5<br>*Field max*: 50<br>*Measurement Unit*: cmH2O	cmH2O

9.2.1.2.3.4	***If 'Mode of ventilation' is equal to 'Spontaneous' answer this question:***<br>Pressure support<br>*Warning shown if field's value is smaller than 5: 'Low value! Please control data input or make a comment.'*<br>*Warning shown if field's value is larger than 19: 'High value! Please control data input or make a comment.'*<br>*Field type*: Numeric field<br>*Variable name*: Psup_1<br>*Field required*: Required<br>*Field min*: 0<br>*Field max*: 30<br>*Measurement Unit*: cmH2O	[ ] cmH2O
9.2.1.2.7	***If 'Type of respiratory support' is equal to 'Mechanical ventilation' answer this question:***<br>Unit used for EtCO2, PaO2 and PaCO2<br>*Field type*: Radiobutton<br>*Variable name*: mmhg_kpa_1<br>*Field required*: Required<br>*Option group name*: kPa/mmHg	○ kPa<br>○ mmHg
9.2.1.2.7.1	***If 'Unit used for EtCO2, PaO2 and PaCO2' is equal to 'kPa' answer this question:***<br>EtCO2<br>*Warning shown if field's value is smaller than 2: 'Low value! Please control data input or make a comment.'*<br>*Warning shown if field's value is larger than 8: 'High value! Please control data input or make a comment.'*<br>*Field type*: Numeric field<br>*Variable name*: EtCO2_kpa_1<br>*Field required*: Required<br>*Field min*: 1<br>*Field max*: 20<br>*Measurement Unit*: kPa	[ ] kPa
9.2.1.2.7.2	***If 'Unit used for EtCO2, PaO2 and PaCO2' is equal to 'mmHg' answer this question:***<br>EtCO2<br>*Warning shown if field's value is smaller than 15: 'Low value! Please control data input or make a comment.'*<br>*Warning shown if field's value is larger than 59: 'High value! Please control data input or make a comment.'*<br>*Field type*: Numeric field<br>*Variable name*: EtCO2_mmhg_1<br>*Field required*: Required<br>*Field min*: 7<br>*Field max*: 140<br>*Measurement Unit*: mmHg	[ ] mmHg

9.2.1.2.8	***If 'Type of respiratory support' is equal to 'Mechanical ventilation' answer this question:***<br>FiO2 (closest to AGB, in decimal, e.g. 0.35)<br>*Field type*: Numeric field<br>*Variable name*: FiO2_1<br>*Field required*: Required<br>*Field min*: 0.2099999999999999922284388<br>*Field max*: 1	
9.2.1.2.9	***If 'Type of respiratory support' is equal to 'Mechanical ventilation' answer this question:***<br>SpO2 (closest to AGB)<br>*Notice shown if field's value is smaller than 80: 'Low value! Please control data input or make a comment.'*<br>*Field type*: Numeric field<br>*Variable name*: SpO2_1<br>*Field required*: Required<br>*Field min*: 60<br>*Field max*: 100<br>*Measurement Unit*: %	%
9.2.1.2.10	***If 'Type of respiratory support' is equal to 'Mechanical ventilation' answer this question:***<br>Recruitment maneuvre on this day<br>*Field type*: Radiobutton<br>*Variable name*: Recruit_1<br>*Field required*: Required<br>*Option group name*: Yes/No	○ Yes<br>○ No
9.2.1.2.10.1	***If 'Recruitment maneuvre on this day' is equal to 'Yes' answer this question:***<br>Amount of recruitment maneuvres<br>*Warning shown if field's value is larger than 9: 'High value! Please control data input or make a comment'*<br>Times on this day from 00:00 until 23:59.<br>*Field type*: Numeric field<br>*Variable name*: Recruit_times_1<br>*Field required*: Required<br>*Field min*: 0<br>*Field max*: 20<br>*Measurement Unit*: times	times
9.2.1.2.11	***If 'Type of respiratory support' is equal to 'Mechanical ventilation' answer this question:***<br>Prone positioning on this day<br>*Field type*: Radiobutton<br>*Variable name*: Prone_1<br>*Field required*: Required<br>*Option group name*: Yes/No	○ Yes<br>○ No

9.2.1.2.11.1	***If 'Prone positioning on this day' is equal to 'Yes' answer this question:***<br>Duration of prone posistioning<br>Hours on this day from 00:00 until 23:59.<br><br>*Field type*: Numeric field<br>*Variable name*: Prone_hours_1<br>*Field required*: Required<br>*Field min*: 0<br>*Field max*: 24<br>*Measurement Unit*: hours	[ ] hours
9.2.1.2.12	***If 'Type of respiratory support' is equal to 'Mechanical ventilation' answer this question:***<br>Do you expect extubation within 48 hours based on the respiration parameters?<br>If you expect extubation within 48 hours based on the respiration parameters, the ventilation strategy can be abandoned. Please look in our mechanical ventilation handbook for guidelines. When the patient's condition worsens, restart the ventilation strategy according to the protocol.<br><br>*Field type*: Radiobutton<br>*Variable name*: ext_1<br>*Field required*: Required<br>*Option group name*: Yes/No	○ Yes<br>○ No
	**ARTERIAL BLOOD GAS AT 6:00**	
9.2.1.2.14	***If 'Type of respiratory support' is equal to 'Mechanical ventilation' answer this question:***<br>Arterial pH<br>*Warning shown if field's value is smaller than 7.0: 'Low value! Please control data input or make a comment.'*<br>*Warning shown if field's value is larger than 7.7: 'High value! Please control data input or make a comment.'*<br>*Field type*: Numeric field<br>*Variable name*: ArtpH_1<br>*Field required*: Required<br>*Field min*: 6.5<br>*Field max*: 8<br>*Measurement Unit*: pH	[ ] pH
9.2.1.2.15	***If 'Type of respiratory support' is equal to 'Mechanical ventilation' answer this question:***<br>Arterial bicarbonate<br>*Warning shown if field's value is smaller than 10: 'Low value! Please control data input or make a comment.'*<br>*Warning shown if field's value is larger than 39: 'High value! Please control data input or make a comment.'*<br>*Field type*: Numeric field<br>*Variable name*: Bic_1<br>*Field required*: Required<br>*Field min*: 2<br>*Field max*: 70<br>*Measurement Unit*: mmol/l	[ ] mmol/l

9.2.1.2.16	***If 'Type of respiratory support' is equal to 'Mechanical ventilation' answer this question:***<br>Arterial lactate<br>*Warning shown if field's value is larger than 9.9: 'High value! Please control data input or make a comment.'*<br>*Field type*: Numeric field<br>*Variable name*: Lac_1<br>*Field required*: Required<br>*Field min*: 0<br>*Field max*: 25<br>*Measurement Unit*: mmol/L	mmol/L
9.2.1.2.17	***If 'Type of respiratory support' is equal to 'Mechanical ventilation' answer this question:***<br>Arterial saturation<br>*Notice shown if field's value is smaller than 80: 'Low value! Please control data input or make a comment.'*<br>*Field type*: Numeric field<br>*Variable name*: Sat_1<br>*Field required*: Required<br>*Field min*: 60<br>*Field max*: 100<br>*Measurement Unit*: %	%
9.2.1.2.7.3	***If 'Unit used for EtCO2, PaO2 and PaCO2' is equal to 'kPa' answer this question:***<br>Arterial PaO2<br>*Warning shown if field's value is smaller than 7: 'Low value! Please control data input or make a comment.'*<br>*Warning shown if field's value is larger than 18: 'High value! Please control data input or make a comment.'*<br>*Field type*: Numeric field<br>*Variable name*: PaO2_kpa_1<br>*Field required*: Required<br>*Field min*: 4<br>*Field max*: 60<br>*Measurement Unit*: kPa	kPa
9.2.1.2.7.4	***If 'Unit used for EtCO2, PaO2 and PaCO2' is equal to 'mmHg' answer this question:***<br>Arterial PaO2<br>*Warning shown if field's value is smaller than 50: 'Low value! Please control data input or make a comment.'*<br>*Warning shown if field's value is larger than 150: 'High value! Please control data input or make a comment.'*<br>*Field type*: Numeric field<br>*Variable name*: PaO2_mmhg_1<br>*Field required*: Required<br>*Field min*: 28<br>*Field max*: 500<br>*Measurement Unit*: mmHg	mmHg

9.2.1.2.7.5	***If 'Unit used for EtCO2, PaO2 and PaCO2' is equal to 'kPa' answer this question:***<br>Arterial PaCO2<br>*Warning shown if field's value is smaller than 2: 'Low value! Please control data input or make a comment.'*<br>*Warning shown if field's value is larger than 8: 'High value! Please control data input or make a comment.'*<br>*Field type*: Numeric field<br>*Variable name*: PaCO2_kpa_1<br>*Field required*: Required<br>*Field min*: 1<br>*Field max*: 20<br>*Measurement Unit*: kPa	kPa
9.2.1.2.7.6	***If 'Unit used for EtCO2, PaO2 and PaCO2' is equal to 'mmHg' answer this question:***<br>Arterial PaCO2<br>*Warning shown if field's value is smaller than 15: 'Low value! Please control data input or make a comment.'*<br>*Warning shown if field's value is larger than 59: 'High value! Please control data input or make a comment.'*<br>*Field type*: Numeric field<br>*Variable name*: PaCO2_mmhg_1<br>*Field required*: Required<br>*Field min*: 7<br>*Field max*: 140<br>*Measurement Unit*: mmHg	mmHg

10. Daily data - Day 2

Number	Question	Answers
10.1	Date of study day<br>*Field type*: Calculation<br>*Variable name*: date_2<br>*Field required*: Not required	*Template:* var randomization= moment('{castorRandomizedDateAndTime}', 'DD-MM-YYYY'); var newDate = randomization.add(2, 'days'); newDate.format('DD-MM-YYYY');
10.2	***If 'Is the patient hospitalized during any time of the day?' is equal to 'Yes' answer this question:***<br>Is the patient hospitalized during any time of the day?<br>*Notice shown if field's value is equal to No: 'DAILY DATA COMPLETE, GO TO FOLLOW UP.'*<br>*Field type*: Radiobutton<br>*Variable name*: Hospital_patient_2<br>*Field required*: Required<br>*Option group name*: Yes/No	○ Yes<br>○ No

10.2.1	***If 'Is the patient hospitalized during any time of the day?' is equal to 'Yes' answer this question:***<br>Location of the patient<br>At 6:00 AM<br><br>*Field type*: Radiobutton<br>*Variable name*: Loc_ICUhosp_2<br>*Field required*: Required<br>*Option group name*: ICU/hospital	◯ Intensive Care Unit<br>◯ Hospital ward
10.2.1.1	***If 'Location of the patient' is equal to 'Hospital ward' answer this question:***<br>Type of respiratory support<br>At 6:00 AM<br><br>*Field type*: Radiobutton<br>*Variable name*: Resp_hospsupp_2<br>*Field required*: Required<br>*Option group name*: resp_supp_hosp	◯ No oxygen therapy<br>◯ Oxygen by mask or nasal prongs<br>◯ High Flow Nasal Oxygen (HFNO) therapy<br>◯ Non-invasive ventilation (NIV)
10.2.1.2	***If 'Location of the patient' is equal to 'Intensive Care Unit' answer this question:***<br>Type of respiratory support<br>At 6:00 AM<br><br>*Field type*: Radiobutton<br>*Variable name*: Resp_supp_2<br>*Field required*: Required<br>*Option group name*: Respiratory support	◯ No oxygen therapy<br>◯ Oxygen by maks or nasal prongs<br>◯ High Flow Nasal Oxygen (HFNO) therapy<br>◯ Non-invasive ventilation (NIV)<br>◯ Mechanical ventilation
10.2.1.3	***If 'Location of the patient' is equal to 'Intensive Care Unit' answer this question:***<br>Daily cumulative fluid balance<br>*Field type*: Radiobutton<br>*Variable name*: Cum_fluid_2<br>*Field required*: Required<br>*Option group name*: Negative/positve	◯ Negative<br>◯ Positive
10.2.1.4	***If 'Location of the patient' is equal to 'Intensive Care Unit' answer this question:***<br>Daily cumulative fluid balance<br>*Warning shown if field's value is larger than or equal to 4000: 'High value! Please control data input or make a comment.'*<br>From 0:00 until 0:00<br><br>*Field type*: Numeric field<br>*Variable name*: Cum_fluid_ml_2<br>*Field required*: Required<br>*Field min*: 0<br>*Field max*: 30000<br>*Measurement Unit*: ml	[ ] ml
	**SOFA SCORE**	

10.2.1.6	***If 'Location of the patient' is equal to 'Intensive Care Unit' answer this question:***<br>PaO2/FiO2 (mmHg (kPa))<br>Worst value of this day. If there is no value for this day please enter missing data.<br><br>*Field type*: Radiobutton<br>*Variable name*: SOFA_Resp_2<br>*Field required*: Required<br>*Option group name*: Respiration	◯ ≥ 400 (53.3)<br>◯ < 400 (53.3)<br>◯ < 300 (40)<br>◯ < 200 (26.7) with respiratory support<br>◯ < 100 (13.3) with respiratory support
10.2.1.7	***If 'Location of the patient' is equal to 'Intensive Care Unit' answer this question:***<br>Platelets (x10^3/µL)<br>Worst value of this day. If there is no value for this day please enter missing data.<br><br>*Field type*: Radiobutton<br>*Variable name*: SOFA_Coag_2<br>*Field required*: Required<br>*Option group name*: Coagulation	◯ ≥ 150<br>◯ < 150<br>◯ < 100<br>◯ < 50<br>◯ < 20
10.2.1.8	***If 'Location of the patient' is equal to 'Intensive Care Unit' answer this question:***<br>Bilirubin (µmol/L(mg/dL))<br>Worst value of this day. If there is no value for this day please enter missing data.<br><br>*Field type*: Radiobutton<br>*Variable name*: SOFA_Live_2<br>*Field required*: Required<br>*Option group name*: Liver	◯ < 20 (< 1.2)<br>◯ 20-32 (1.2-1.9)<br>◯ 33-101 (2.0-5.9)<br>◯ 102-204 (6.0-11.9)<br>◯ > 204 (> 12.0)
10.2.1.9	***If 'Location of the patient' is equal to 'Intensive Care Unit' answer this question:***<br>Cardiovascular<br>Worst value of this day. If there is no value for this day please enter missing data.<br><br>*Field type*: Radiobutton<br>*Variable name*: SOFA_Card_2<br>*Field required*: Required<br>*Option group name*: Cardiovascular	◯ MAP ≥ 70 mm/Hg without dobutamine, epinephrine or norepinephrine<br>◯ MAP < 70 mm/Hg without dobutamine, epinephrine or norepinephrin<br>◯ Dopamine < 5 µg/kg/min or dobutamine (any dose)<br>◯ Dopamine > 5 µg/kg/min OR epinephrine ≤ 0.1 µg/kg/min OR norepinephrine ≤ 0.1 µg/kg/min<br>◯ Dopamine > 15 µg/kg/min OR epinephrine > 0.1 µg/kg/min OR norepinephrine > 0.1 µg/kg/min
10.2.1.10	***If 'Location of the patient' is equal to 'Intensive Care Unit' answer this question:***<br>Glasgow coma scale<br>Only fill in the score when the patient is not sedated. Choose the worst value of this day. If there is no value for this day or the patient is sedated, please enter missing data.<br><br>*Field type*: Radiobutton<br>*Variable name*: SOFA_Nerv_2<br>*Field required*: Required<br>*Option group name*: Nervous	◯ 15<br>◯ 13-14<br>◯ 10-12<br>◯ 6-9<br>◯ < 6

10.2.1.11

If 'Location of the patient' is equal to 'Intensive Care Unit' answer this question:
Creatinine [or urine output]
Worst value of this day. If there is no value for this day please enter missing data.

Field type: Radiobutton
Variable name: SOFA_Kidn_2
Field required: Required
Option group name: Kidneys

- ◯ < 110 (< 1.2)
- ◯ 110-170 (1.2-1.9)
- ◯ 171-299 (2.0-3.4)
- ◯ 300-440 (3.5-4.9) [or < 500]
- ◯ > 440 (> 5.0) [or < 200]

10.2.1.12

If 'Location of the patient' is equal to 'Intensive Care Unit' answer this question:
SOFA score
Field type: Calculation
Variable name: SOFA_scor_2
Field required: Not required

Template: {SOFA_Resp_2}+{SOFA_Coag_2}+{SOFA_Live_2}+{SOFA_Card_2}+{SOFA_Nerv_2}+{SOFA_Kidn_2}

EVENTS

10.2.1.14

If 'Location of the patient' is equal to 'Intensive Care Unit' answer this question:
Did an event occur?

- Renal Replacement Therapy during any time of the day.
- Placement of a tracheostomy during any time of the day
- Use of inhaled vasodilators during any time of the day
- Use of airway pressure release ventlation during any time of the day
- Use of ECMO during any time of the day

Field type: Checkbox
Variable name: Event_2
Field required: Required
Option group name: Events

- ☐ None
- ☐ Use of renal replacement therapy
- ☐ Placement of a tracheostomy
- ☐ Use of inhaled vasodilators
- ☐ Use of airway pressure release ventilation
- ☐ Use of ECMO
- ☐ Continuous infusion of neuromuscular blocking agents

10.2.1.15

If 'Location of the patient' is equal to 'Intensive Care Unit' answer this question:
Calculation for incorrect answer event
Field type: Calculation
Variable name: Event_calc_incorrect_2
Field required: Not required

Template: '##allowempty##' var splitted = "{Event_2}".split(';'); if (splitted.indexOf("0") > -1 && (splitted.indexOf("1") > -1 || splitted.indexOf("2") > -1 || splitted.indexOf("3") > -1 || splitted.indexOf("4") > -1 || splitted.indexOf("5") > -1 || splitted.indexOf("6") > -1)) { '1'; } else { '0'; };

Combination **NOT** possible! **NONE** cannot be chosen when other options are selected!

COMPLICATIONS

10.2.1.17	***If 'Location of the patient' is equal to 'Intensive Care Unit' answer this question:***<br>Did a complication occur?<br>Only tick the checkbox at the day of diagnosis.<br>• Pneumothorax: Air in the pleural cavity developed after randomization confirmed by a radiologist on a CT-thorax or chest radiograph for which a drain has been placed.<br>• Ventilator Associated Pneumonia: Clinical Pulmonary Infection Score (CPIS) > 5 with an infiltration on CXR and developed after intubation.<br>*Field type*: Checkbox<br>*Variable name*: Compl_2<br>*Field required*: Required<br>*Option group name*: Complications	☐ None<br>☐ Diagnosis of pneumothorax<br>☐ Diagnosis of Ventilator Associated Pneumonia
10.2.1.18	***If 'Location of the patient' is equal to 'Intensive Care Unit' answer this question:***<br>Calculation for incorrect answer complications<br>*Field type*: Calculation<br>*Variable name*: Compl_calc_incorrect_2<br>*Field required*: Not required	*Template:* '##allowempty##' var splitted = "{Compl_2}".split(';'); if (splitted.indexOf("0") > -1 && (splitted.indexOf("1") > -1 || splitted.indexOf("2") > -1 || splitted.indexOf("3") > -1 || splitted.indexOf("4") > -1 || splitted.indexOf("5") > -1 || splitted.indexOf("6") > -1)) { '1'; } else { '0'; };
	Combination **NOT** possible! **NONE** cannot be chosen when other options are selected!	
	**VENTILATION DATA AT 6:00 AM**	
10.2.1.2.2	***If 'Type of respiratory support' is equal to 'Mechanical ventilation' answer this question:***<br>RASS score<br>+4 Combative +3 Very agitated +2 Agitated +1 Restless 0 Alert and calm -1 Drowsy -2 Light sedation -3 Moderate sedation -4 Deep sedation -5 Unarousable<br>*Field type*: Radiobutton<br>*Variable name*: Rass_2<br>*Field required*: Required<br>*Option group name*: RASS Score	○ ≥ 0<br>○ - 1<br>○ - 2<br>○ - 3<br>○ - 4<br>○ - 5
10.2.1.2.3	***If 'Type of respiratory support' is equal to 'Mechanical ventilation' answer this question:***<br>Mode of ventilation<br>*Field type*: Radiobutton<br>*Variable name*: Mode_ven_2<br>*Field required*: Required<br>*Option group name*: Mode ventilation	○ Controlled<br>○ Spontaneous<br>○ Adaptive
10.2.1.2.3.1	***If 'Mode of ventilation' is equal to 'Adaptive' answer this question:***<br>Triggered or timed adaptive ventilation<br>*Field type*: Radiobutton<br>*Variable name*: Trig_timed_2<br>*Field required*: Required<br>*Option group name*: Triggered/Timed	○ Triggered<br>○ Timed

10.2.1.2.4	***If 'Type of respiratory support' is equal to 'Mechanical ventilation' answer this question:***<br>Tidal volume<br>*Warning shown if field's value is smaller than 100: 'Low value! Please control data input or make a comment.'*<br>*Warning shown if field's value is larger than 900: 'High value! Please control data input or make a comment.'*<br>*Field type*: Numeric field<br>*Variable name*: Tidal_2<br>*Field required*: Required<br>*Field min*: 50<br>*Field max*: 1200<br>*Measurement Unit*: ml	ml
10.2.1.2.5	***If 'Type of respiratory support' is equal to 'Mechanical ventilation' answer this question:***<br>Total respiratory rate<br>*Warning shown if field's value is smaller than 10: 'Low value! Please control data input or make a comment.'*<br>*Warning shown if field's value is larger than 35: 'High value! Please control data input or make a comment.'*<br>*Field type*: Numeric field<br>*Variable name*: TRR_2<br>*Field required*: Required<br>*Field min*: 4<br>*Field max*: 50<br>*Measurement Unit*: breaths per minut	breaths per minut
10.2.1.2.6	***If 'Type of respiratory support' is equal to 'Mechanical ventilation' answer this question:***<br>PEEP<br>*Warning shown if field's value is smaller than 5: 'Low value! Please control data input or make a comment.'*<br>*Warning shown if field's value is larger than 19: 'High value! Please control data input or make a comment.'*<br>*Field type*: Numeric field<br>*Variable name*: Peep_2<br>*Field required*: Required<br>*Field min*: 0<br>*Field max*: 25<br>*Measurement Unit*: cmH2O	cmH2O
10.2.1.2.3.2	***If 'Mode of ventilation' is not equal to 'Spontaneous' answer this question:***<br>Pplateau<br>*Warning shown if field's value is smaller than or equal to 7: 'Low value! Please control data input or make a comment.'*<br>*Warning shown if field's value is larger than or equal to 30: 'High value! Please control data input or make a comment.'*<br>*Field type*: Numeric field<br>*Variable name*: Pplat_2<br>*Field required*: Required<br>*Field min*: 5<br>*Field max*: 40<br>*Measurement Unit*: cmH2O	cmH2O

10.2.1.2.3.3	***If 'Mode of ventilation' is not equal to 'Spontaneous' answer this question:***<br>Pmax/ Ppeak<br>*Warning shown if field's value is smaller than 10: 'Low value! Please control data input or make a comment.'*<br>*Warning shown if field's value is larger than 29: 'High value! Please control data input or make a comment.'*<br>*Field type*: Numeric field<br>*Variable name*: Pmax_2<br>*Field required*: Required<br>*Field min*: 5<br>*Field max*: 50<br>*Measurement Unit*: cmH2O	[ ] cmH2O
10.2.1.2.3.4	***If 'Mode of ventilation' is equal to 'Spontaneous' answer this question:***<br>Pressure support<br>*Warning shown if field's value is smaller than 5: 'Low value! Please control data input or make a comment.'*<br>*Warning shown if field's value is larger than 19: 'High value! Please control data input or make a comment.'*<br>*Field type*: Numeric field<br>*Variable name*: Psup_2<br>*Field required*: Required<br>*Field min*: 0<br>*Field max*: 30<br>*Measurement Unit*: cmH2O	[ ] cmH2O
10.2.1.2.7	***If 'Type of respiratory support' is equal to 'Mechanical ventilation' answer this question:***<br>Unit used for EtCO2, PaO2 and PaCO2<br>*Field type*: Radiobutton<br>*Variable name*: mmhg_kpa_2<br>*Field required*: Required<br>*Option group name*: kPa/mmHg	○ kPa<br>○ mmHg
10.2.1.2.7.1	***If 'Unit used for EtCO2, PaO2 and PaCO2' is equal to 'kPa' answer this question:***<br>EtCO2<br>*Warning shown if field's value is smaller than 2: 'Low value! Please control data input or make a comment.'*<br>*Warning shown if field's value is larger than 8: 'High value! Please control data input or make a comment.'*<br>*Field type*: Numeric field<br>*Variable name*: EtCO2_kpa_2<br>*Field required*: Required<br>*Field min*: 1<br>*Field max*: 20<br>*Measurement Unit*: kPa	[ ] kPa

10.2.1.2.7.2	***If 'Unit used for EtCO2, PaO2 and PaCO2' is equal to 'mmHg' answer this question:***<br>EtCO2<br>*Warning shown if field's value is smaller than 15: 'Low value! Please control data input or make a comment.'*<br>*Warning shown if field's value is larger than 59: 'High value! Please control data input or make a comment.'*<br>*Field type*: Numeric field<br>*Variable name*: EtCO2_mmhg_2<br>*Field required*: Required<br>*Field min*: 7<br>*Field max*: 140<br>*Measurement Unit*: mmHg	[ ] mmHg
10.2.1.2.8	***If 'Type of respiratory support' is equal to 'Mechanical ventilation' answer this question:***<br>FiO2 (closest to AGB, in decimal, e.g. 0.35)<br>*Field type*: Numeric field<br>*Variable name*: FiO2_2<br>*Field required*: Required<br>*Field min*: 0.2099999999999999922284388<br>*Field max*: 1	[ ]
10.2.1.2.9	***If 'Type of respiratory support' is equal to 'Mechanical ventilation' answer this question:***<br>SpO2 (closest to AGB)<br>*Notice shown if field's value is smaller than 80: 'Low value! Please control data input or make a comment.'*<br>*Field type*: Numeric field<br>*Variable name*: SpO2_2<br>*Field required*: Required<br>*Field min*: 60<br>*Field max*: 100<br>*Measurement Unit*: %	[ ] %
10.2.1.2.10	***If 'Type of respiratory support' is equal to 'Mechanical ventilation' answer this question:***<br>Recruitment maneuvre on this day<br>*Field type*: Radiobutton<br>*Variable name*: Recruit_2<br>*Field required*: Required<br>*Option group name*: Yes/No	○ Yes<br>○ No
10.2.1.2.10.1	***If 'Recruitment maneuvre on this day' is equal to 'Yes' answer this question:***<br>Amount of recruitment maneuvres<br>*Warning shown if field's value is larger than 9: 'High value! Please control data input or make a comment'*<br>Times on this day from 00:00 until 23:59.<br>*Field type*: Numeric field<br>*Variable name*: Recruit_times_2<br>*Field required*: Required<br>*Field min*: 0<br>*Field max*: 20<br>*Measurement Unit*: times	[ ] times

10.2.1.2.11	***If 'Type of respiratory support' is equal to 'Mechanical ventilation' answer this question:***<br>Prone positioning on this day<br>*Field type*: Radiobutton<br>*Variable name*: Prone_2<br>*Field required*: Required<br>*Option group name*: Yes/No	◯ Yes<br>◯ No
10.2.1.2.11.1	***If 'Prone positioning on this day' is equal to 'Yes' answer this question:***<br>Duration of prone posistioning<br>Hours on this day from 00:00 until 23:59.<br><br>*Field type*: Numeric field<br>*Variable name*: Prone_hours_2<br>*Field required*: Required<br>*Field min*: 0<br>*Field max*: 24<br>*Measurement Unit*: hours	[ ] hours
10.2.1.2.12	***If 'Type of respiratory support' is equal to 'Mechanical ventilation' answer this question:***<br>Do you expect extubation within 48 hours based on the respiration parameters?<br>If you expect extubation within 48 hours based on the respiration parameters, the ventilation strategy can be abandoned. Please look in our mechanical ventilation handbook for guidelines. When the patient's condition worsens, restart the ventilation strategy according to the protocol.<br><br>*Field type*: Radiobutton<br>*Variable name*: ext_2<br>*Field required*: Required<br>*Option group name*: Yes/No	◯ Yes<br>◯ No
	**ARTERIAL BLOOD GAS AT 6:00**	
10.2.1.2.14	***If 'Type of respiratory support' is equal to 'Mechanical ventilation' answer this question:***<br>Arterial pH<br>*Warning shown if field's value is smaller than 7.0: 'Low value! Please control data input or make a comment.'*<br>*Warning shown if field's value is larger than 7.7: 'High value! Please control data input or make a comment.'*<br>*Field type*: Numeric field<br>*Variable name*: ArtpH_2<br>*Field required*: Required<br>*Field min*: 6.5<br>*Field max*: 8<br>*Measurement Unit*: pH	[ ] pH

10.2.1.2.15	***If 'Type of respiratory support' is equal to 'Mechanical ventilation' answer this question:***<br>Arterial bicarbonate<br>*Warning shown if field's value is smaller than 10: 'Low value! Please control data input or make a comment.'*<br>*Warning shown if field's value is larger than 39: 'High value! Please control data input or make a comment.'*<br>*Field type*: Numeric field<br>*Variable name*: Bic_2<br>*Field required*: Required<br>*Field min*: 2<br>*Field max*: 70<br>*Measurement Unit*: mmol/l	mmol/l
10.2.1.2.16	***If 'Type of respiratory support' is equal to 'Mechanical ventilation' answer this question:***<br>Arterial lactate<br>*Warning shown if field's value is larger than 9.9: 'High value! Please control data input or make a comment.'*<br>*Field type*: Numeric field<br>*Variable name*: Lac_2<br>*Field required*: Required<br>*Field min*: 0<br>*Field max*: 25<br>*Measurement Unit*: mmol/L	mmol/L
10.2.1.2.17	***If 'Type of respiratory support' is equal to 'Mechanical ventilation' answer this question:***<br>Arterial saturation<br>*Notice shown if field's value is smaller than 80: 'Low value! Please control data input or make a comment.'*<br>*Field type*: Numeric field<br>*Variable name*: Sat_2<br>*Field required*: Required<br>*Field min*: 60<br>*Field max*: 100<br>*Measurement Unit*: %	%
10.2.1.2.7.3	***If 'Unit used for EtCO2, PaO2 and PaCO2' is equal to 'kPa' answer this question:***<br>Arterial PaO2<br>*Warning shown if field's value is smaller than 7: 'Low value! Please control data input or make a comment.'*<br>*Warning shown if field's value is larger than 18: 'High value! Please control data input or make a comment.'*<br>*Field type*: Numeric field<br>*Variable name*: PaO2_kpa_2<br>*Field required*: Required<br>*Field min*: 4<br>*Field max*: 60<br>*Measurement Unit*: kPa	kPa

10.2.1.2.7.4	***If 'Unit used for EtCO2, PaO2 and PaCO2' is equal to 'mmHg' answer this question:***<br>Arterial PaO2<br>*Warning shown if field's value is smaller than 50: 'Low value! Please control data input or make a comment.'*<br>*Warning shown if field's value is larger than 150: 'High value! Please control data input or make a comment.'*<br>*Field type*: Numeric field<br>*Variable name*: PaO2_mmhg_2<br>*Field required*: Required<br>*Field min*: 28<br>*Field max*: 500<br>*Measurement Unit*: mmHg	[ ] mmHg
10.2.1.2.7.5	***If 'Unit used for EtCO2, PaO2 and PaCO2' is equal to 'kPa' answer this question:***<br>Arterial PaCO2<br>*Warning shown if field's value is smaller than 2: 'Low value! Please control data input or make a comment.'*<br>*Warning shown if field's value is larger than 8: 'High value! Please control data input or make a comment.'*<br>*Field type*: Numeric field<br>*Variable name*: PaCO2_kpa_2<br>*Field required*: Required<br>*Field min*: 1<br>*Field max*: 20<br>*Measurement Unit*: kPa	[ ] kPa
10.2.1.2.7.6	***If 'Unit used for EtCO2, PaO2 and PaCO2' is equal to 'mmHg' answer this question:***<br>Arterial PaCO2<br>*Warning shown if field's value is smaller than 15: 'Low value! Please control data input or make a comment.'*<br>*Warning shown if field's value is larger than 59: 'High value! Please control data input or make a comment.'*<br>*Field type*: Numeric field<br>*Variable name*: PaCO2_mmhg_2<br>*Field required*: Required<br>*Field min*: 7<br>*Field max*: 140<br>*Measurement Unit*: mmHg	[ ] mmHg

11. Daily data - Day 3

Number	Question	Answers
11.1	Date of study day<br>*Field type*: Calculation<br>*Variable name*: date_3<br>*Field required*: Not required	*Template:* var randomization= moment('{castorRandomizedDateAndTime}', 'DD-MM-YYYY'); var newDate = randomization.add(3, 'days'); newDate.format('DD-MM-YYYY');

11.2	***If 'Is the patient hospitalized during any time of the day?' is equal to 'Yes' answer this question:***<br>Is the patient hospitalized during any time of the day?<br>*Notice shown if field's value is equal to No: 'DAILY DATA COMPLETE, GO TO FOLLOW UP.'*<br>*Field type*: Radiobutton<br>*Variable name*: Hospital_patient_3<br>*Field required*: Required<br>*Option group name*: Yes/No	◯ Yes<br>◯ No
11.2.1	***If 'Is the patient hospitalized during any time of the day?' is equal to 'Yes' answer this question:***<br>Location of the patient<br>At 6:00 AM<br><br>*Field type*: Radiobutton<br>*Variable name*: Loc_ICUhosp_3<br>*Field required*: Required<br>*Option group name*: ICU/hospital	◯ Intensive Care Unit<br>◯ Hospital ward
11.2.1.1	***If 'Location of the patient' is equal to 'Hospital ward' answer this question:***<br>Type of respiratory support<br>At 6:00 AM<br><br>*Field type*: Radiobutton<br>*Variable name*: Resp_hospsupp_3<br>*Field required*: Required<br>*Option group name*: resp_supp_hosp	◯ No oxygen therapy<br>◯ Oxygen by mask or nasal prongs<br>◯ High Flow Nasal Oxygen (HFNO) therapy<br>◯ Non-invasive ventilation (NIV)
11.2.1.2	***If 'Location of the patient' is equal to 'Intensive Care Unit' answer this question:***<br>Type of respiratory support<br>At 6:00 AM<br><br>*Field type*: Radiobutton<br>*Variable name*: Resp_supp_3<br>*Field required*: Required<br>*Option group name*: Respiratory support	◯ No oxygen therapy<br>◯ Oxygen by maks or nasal prongs<br>◯ High Flow Nasal Oxygen (HFNO) therapy<br>◯ Non-invasive ventilation (NIV)<br>◯ Mechanical ventilation
11.2.1.3	***If 'Location of the patient' is equal to 'Intensive Care Unit' answer this question:***<br>Daily cumulative fluid balance<br>*Field type*: Radiobutton<br>*Variable name*: Cum_fluid_3<br>*Field required*: Required<br>*Option group name*: Negative/positve	◯ Negative<br>◯ Positive

11.2.1.4	***If 'Location of the patient' is equal to 'Intensive Care Unit' answer this question:***<br>Daily cumulative fluid balance<br>*Warning shown if field's value is larger than or equal to 4000: 'High value! Please control data input or make a comment.'*<br>From 0:00 until 0:00<br><br>*Field type*: Numeric field<br>*Variable name*: Cum_fluid_ml_3<br>*Field required*: Required<br>*Field min*: 0<br>*Field max*: 30000<br>*Measurement Unit*: ml	[ ] ml
11.3	Calculation FU LUS<br>*Field type*: Calculation<br>*Variable name*: Cal_FU_LUS<br>*Field required*: Not required	*Template:* if ({Confirm_LUS} == 0 && {Conf_rando} == 0 && {Resp_supp_3} == 4) { '0' ; } else { '1' ; }
	**Perform a Follow up LUS exam**	
11.3.2	***If 'Calculation FU LUS' is equal to '0' answer this question:***<br>LUS exam performed by<br>*Field type*: Textfield<br>*Variable name*: LUS_performed_1<br>*Field required*: Required	[ ]
11.3.3	***If 'Calculation FU LUS' is equal to '0' answer this question:***<br>PEEP during LUS exam<br>*Warning shown if field's value is larger than 8: 'LUS exam should be performed with a PEEP level of 5 cmH2O (or max 8 cmH2O with hypoxia)'*<br>*Warning shown if field's value is smaller than 5: 'LUS exam should be performed with a PEEP level of 5 cmH2O (or max 8 cmH2O with hypoxia)'*<br>*Field type*: Numeric field<br>*Variable name*: PEEP_LUS_1<br>*Field required*: Required<br>*Field min*: 3<br>*Field max*: 15<br>*Measurement Unit*: cmH2O	[ ] cmH2O
	RIGHT LUNG	
11.3.5	***If 'Calculation FU LUS' is equal to '0' answer this question:***<br>Anterior region 1<br>*Field type*: Radiobutton<br>*Variable name*: RA1_1<br>*Field required*: Required<br>*Option group name*: LUS exam score	◯ A-pattern<br>◯ B-pattern <50%<br>◯ B-pattern >50%<br>◯ Consolidation

11.3.6 ***If 'Calculation FU LUS' is equal to '0' answer this question:***
Anterior region 2
Field type: Radiobutton
Variable name: RA2_1
Field required: Required
Option group name: LUS exam score

- ◯ A-pattern
- ◯ B-pattern <50%
- ◯ B-pattern >50%
- ◯ Consolidation

11.3.7 ***If 'Calculation FU LUS' is equal to '0' answer this question:***
Lateral region 3
Field type: Radiobutton
Variable name: RL3_1
Field required: Required
Option group name: LUS exam score

- ◯ A-pattern
- ◯ B-pattern <50%
- ◯ B-pattern >50%
- ◯ Consolidation

11.3.8 ***If 'Calculation FU LUS' is equal to '0' answer this question:***
Lateral region 4
Field type: Radiobutton
Variable name: RL4_1
Field required: Required
Option group name: LUS exam score

- ◯ A-pattern
- ◯ B-pattern <50%
- ◯ B-pattern >50%
- ◯ Consolidation

11.3.9 ***If 'Calculation FU LUS' is equal to '0' answer this question:***
Posterior region 5
Field type: Radiobutton
Variable name: RP5_1
Field required: Required
Option group name: LUS exam score

- ◯ A-pattern
- ◯ B-pattern <50%
- ◯ B-pattern >50%
- ◯ Consolidation

11.3.10 ***If 'Calculation FU LUS' is equal to '0' answer this question:***
Posterior region 6
Field type: Radiobutton
Variable name: RP6_1
Field required: Required
Option group name: LUS exam posterior

- ◯ A-pattern or B-pattern < 50%
- ◯ B-pattern > 50%
- ◯ Consolidation

LEFT LUNG

11.3.12 ***If 'Calculation FU LUS' is equal to '0' answer this question:***
Anterior region 1
Field type: Radiobutton
Variable name: LA1_1
Field required: Required
Option group name: LUS exam score

- ◯ A-pattern
- ◯ B-pattern <50%
- ◯ B-pattern >50%
- ◯ Consolidation

11.3.13	***If 'Calculation FU LUS' is equal to '0' answer this question:***<br>Anterior region 2<br>*Field type*: Radiobutton<br>*Variable name*: LA2_1<br>*Field required*: Required<br>*Option group name*: LUS exam score	◯ A-pattern<br>◯ B-pattern <50%<br>◯ B-pattern >50%<br>◯ Consolidation
11.3.14	***If 'Calculation FU LUS' is equal to '0' answer this question:***<br>Lateral region 3<br>*Field type*: Radiobutton<br>*Variable name*: LL3_1<br>*Field required*: Required<br>*Option group name*: LUS exam score	◯ A-pattern<br>◯ B-pattern <50%<br>◯ B-pattern >50%<br>◯ Consolidation
11.3.15	***If 'Calculation FU LUS' is equal to '0' answer this question:***<br>Lateral region 4<br>*Field type*: Radiobutton<br>*Variable name*: LL4_1<br>*Field required*: Required<br>*Option group name*: LUS exam score	◯ A-pattern<br>◯ B-pattern <50%<br>◯ B-pattern >50%<br>◯ Consolidation
11.3.16	***If 'Calculation FU LUS' is equal to '0' answer this question:***<br>Posterior region 5<br>*Field type*: Radiobutton<br>*Variable name*: LP5_1<br>*Field required*: Required<br>*Option group name*: LUS exam score	◯ A-pattern<br>◯ B-pattern <50%<br>◯ B-pattern >50%<br>◯ Consolidation
11.3.17	***If 'Calculation FU LUS' is equal to '0' answer this question:***<br>Posterior region 6<br>*Field type*: Radiobutton<br>*Variable name*: LP6_1<br>*Field required*: Required<br>*Option group name*: LUS exam posterior	◯ A-pattern or B-pattern < 50%<br>◯ B-pattern > 50%<br>◯ Consolidation
	**If there are LUS exam regions missing, use the flowchart below to define the ARDS fenotype**	
11.3.19	***If 'Calculation FU LUS' is equal to '0' answer this question:***<br>Flowchart Ultrasound<br>Missing LUS images are complemented by the mean LUS aeration score of the other available LUS images in the concerning region (anterior, lateral, or posterior region).<br><br>*Field type*: Image<br>*Variable name*: Flow_US_1<br>*Field required*: Not required	

11.3.20	***If 'Calculation FU LUS' is equal to '0' answer this question:***<br>Result LUS exam<br>*Field type*: Calculation<br>*Variable name*: Result_LUS_1<br>*Field required*: Not required	*Template:* if ({cal_anterior_1} >= 2) { 'Non-Focal' } else if ({cal_lateral_1} > {cal_posterior_1}){ 'Non-Focal' } else { 'Focal' }
11.3.21	***If 'Calculation FU LUS' is equal to '0' answer this question:***<br>Confirm result LUS exam<br>*Field type*: Radiobutton<br>*Variable name*: Confirm_LUS_1<br>*Field required*: Required<br>*Option group name*: LUS exam	◯ Focal<br>◯ Non-focal
11.3.22	***If 'Calculation FU LUS' is equal to '0' answer this question:***<br>Calculation anterior<br>*Field type*: Calculation<br>*Variable name*: cal_anterior_1<br>*Field required*: Not required	*Template:* {RA1_1} + {RA2_1} + {LA1_1} + {LA2_1}
11.3.23	***If 'Calculation FU LUS' is equal to '0' answer this question:***<br>Calculation lateral<br>*Field type*: Calculation<br>*Variable name*: cal_lateral_1<br>*Field required*: Not required	*Template:* {RL3_1}+{RL4_1}+{LL3_1}+{LL4_1}
11.3.24	***If 'Calculation FU LUS' is equal to '0' answer this question:***<br>Calculation posterior<br>*Field type*: Calculation<br>*Variable name*: cal_posterior_1<br>*Field required*: Not required	*Template:* {RP5_1}+{RP6_1}+{LP5_1}+{LP6_1}
	**SOFA SCORE**	
11.2.1.6	***If 'Location of the patient' is equal to 'Intensive Care Unit' answer this question:***<br>PaO2/FiO2 (mmHg (kPa))<br>Worst value of this day. If there is no value for this day please enter missing data.<br>*Field type*: Radiobutton<br>*Variable name*: SOFA_Resp_3<br>*Field required*: Required<br>*Option group name*: Respiration	◯ ≥ 400 (53.3)<br>◯ < 400 (53.3)<br>◯ < 300 (40)<br>◯ < 200 (26.7) with respiratory support<br>◯ < 100 (13.3) with respiratory support

11.2.1.7	***If 'Location of the patient' is equal to 'Intensive Care Unit' answer this question:***<br>Platelets (x10^3/µL)<br>Worst value of this day. If there is no value for this day please enter missing data.<br><br>*Field type*: Radiobutton<br>*Variable name*: SOFA_Coag_3<br>*Field required*: Required<br>*Option group name*: Coagulation	◯ ≥ 150<br>◯ < 150<br>◯ < 100<br>◯ < 50<br>◯ < 20
11.2.1.8	***If 'Location of the patient' is equal to 'Intensive Care Unit' answer this question:***<br>Bilirubin (µmol/L(mg/dL))<br>Worst value of this day. If there is no value for this day please enter missing data.<br><br>*Field type*: Radiobutton<br>*Variable name*: SOFA_Live_3<br>*Field required*: Required<br>*Option group name*: Liver	◯ < 20 (< 1.2)<br>◯ 20-32 (1.2-1.9)<br>◯ 33-101 (2.0-5.9)<br>◯ 102-204 (6.0-11.9)<br>◯ > 204 (> 12.0)
11.2.1.9	***If 'Location of the patient' is equal to 'Intensive Care Unit' answer this question:***<br>Cardiovascular<br>Worst value of this day. If there is no value for this day please enter missing data.<br><br>*Field type*: Radiobutton<br>*Variable name*: SOFA_Card_3<br>*Field required*: Required<br>*Option group name*: Cardiovascular	◯ MAP ≥ 70 mm/Hg without dobutamine, epinephrine or norepinephrine<br>◯ MAP < 70 mm/Hg without dobutamine, epinephrine or norepinephrin<br>◯ Dopamine < 5 µg/kg/min or dobutamine (any dose)<br>◯ Dopamine > 5 µg/kg/min OR epinephrine ≤ 0.1 µg/kg/min OR norepinephrine ≤ 0.1 µg/kg/min<br>◯ Dopamine > 15 µg/kg/min OR epinephrine > 0.1 µg/kg/min OR norepinephrine > 0.1 µg/kg/min
11.2.1.10	***If 'Location of the patient' is equal to 'Intensive Care Unit' answer this question:***<br>Glasgow coma scale<br>Only fill in the score when the patient is not sedated. Choose the worst value of this day. If there is no value for this day or the patient is sedated, please enter missing data.<br><br>*Field type*: Radiobutton<br>*Variable name*: SOFA_Nerv_3<br>*Field required*: Required<br>*Option group name*: Nervous	◯ 15<br>◯ 13-14<br>◯ 10-12<br>◯ 6-9<br>◯ < 6
11.2.1.11	***If 'Location of the patient' is equal to 'Intensive Care Unit' answer this question:***<br>Creatinine [or urine output]<br>Worst value of this day. If there is no value for this day please enter missing data.<br><br>*Field type*: Radiobutton<br>*Variable name*: SOFA_Kidn_3<br>*Field required*: Required<br>*Option group name*: Kidneys	◯ < 110 (< 1.2)<br>◯ 110-170 (1.2-1.9)<br>◯ 171-299 (2.0-3.4)<br>◯ 300-440 (3.5-4.9) [or < 500]<br>◯ > 440 (> 5.0) [or < 200]

11.2.1.12	***If 'Location of the patient' is equal to 'Intensive Care Unit' answer this question:***<br>SOFA score<br>*Field type*: Calculation<br>*Variable name*: SOFA_scor_3<br>*Field required*: Not required	*Template:* {SOFA_Resp_3}+{SOFA_Coag_3}+{SOFA_Live_3}+{SOFA_Card_3}+{SOFA_Nerv_3}+{SOFA_Kidn_3}
	**EVENTS**	
11.2.1.14	***If 'Location of the patient' is equal to 'Intensive Care Unit' answer this question:***<br>Did an event occur?<br>• Renal Replacement Therapy during any time of the day.<br>• Placement of a tracheostomy during any time of the day<br>• Use of inhaled vasodilators during any time of the day<br>• Use of airway pressure release ventlation during any time of the day<br>• Use of ECMO during any time of the day<br>*Field type*: Checkbox<br>*Variable name*: Event_3<br>*Field required*: Required<br>*Option group name*: Events	☐ None<br>☐ Use of renal replacement therapy<br>☐ Placement of a tracheostomy<br>☐ Use of inhaled vasodilators<br>☐ Use of airway pressure release ventilation<br>☐ Use of ECMO<br>☐ Continuous infusion of neuromuscular blocking agents
11.2.1.15	***If 'Location of the patient' is equal to 'Intensive Care Unit' answer this question:***<br>Calculation for incorrect answer event<br>*Field type*: Calculation<br>*Variable name*: Event_calc_incorrect_3<br>*Field required*: Not required	*Template:* '##allowempty##' var splitted = "{Event_3}".split(';'); if (splitted.indexOf("0") > -1 && (splitted.indexOf("1") > -1 || splitted.indexOf("2") > -1 || splitted.indexOf("3") > -1 || splitted.indexOf("4") > -1 || splitted.indexOf("5") > -1 || splitted.indexOf("6") > -1)) { '1'; } else { '0'; };
	Combination **NOT** possible! **NONE** cannot be chosen when other options are selected!	
	**COMPLICATIONS**	
11.2.1.17	***If 'Location of the patient' is equal to 'Intensive Care Unit' answer this question:***<br>Did a complication occur?<br>Only tick the checkbox at the day of diagnosis.<br>• Pneumothorax: Air in the pleural cavity developed after randomization confirmed by a radiologist on a CT-thorax or chest radiograph for which a drain has been placed.<br>• Ventilator Associated Pneumonia: Clinical Pulmonary Infection Score (CPIS) > 5 with an infiltration on CXR and developed after intubation.<br>*Field type*: Checkbox<br>*Variable name*: Compl_3<br>*Field required*: Required<br>*Option group name*: Complications	☐ None<br>☐ Diagnosis of pneumothorax<br>☐ Diagnosis of Ventilator Associated Pneumonia

11.2.1.18	***If 'Location of the patient' is equal to 'Intensive Care Unit' answer this question:***<br>Calculation for incorrect answer complications<br>*Field type*: Calculation<br>*Variable name*: Compl_calc_incorrect_3<br>*Field required*: Not required	*Template:* '##allowempty##' var splitted = "{Compl_3}".split(';'); if (splitted.indexOf("0") > -1 && (splitted.indexOf("1") > -1 || splitted.indexOf("2") > -1 || splitted.indexOf("3") > -1 || splitted.indexOf("4") > -1 || splitted.indexOf("5") > -1 || splitted.indexOf("6") > -1)) { '1'; } else { '0'; };
	Combination **NOT** possible! **NONE** cannot be chosen when other options are selected!	
	**VENTILATION DATA AT 6:00 AM**	
11.2.1.2.2	***If 'Type of respiratory support' is equal to 'Mechanical ventilation' answer this question:***<br>RASS score<br>+4 Combative +3 Very agitated +2 Agitated +1 Restless 0 Alert and calm -1 Drowsy -2 Light sedation -3 Moderate sedation -4 Deep sedation -5 Unarousable<br>*Field type*: Radiobutton<br>*Variable name*: Rass_3<br>*Field required*: Required<br>*Option group name*: RASS Score	◯ ≥ 0<br>◯ - 1<br>◯ - 2<br>◯ - 3<br>◯ - 4<br>◯ - 5
11.2.1.2.3	***If 'Type of respiratory support' is equal to 'Mechanical ventilation' answer this question:***<br>Mode of ventilation<br>*Field type*: Radiobutton<br>*Variable name*: Mode_ven_3<br>*Field required*: Required<br>*Option group name*: Mode ventilation	◯ Controlled<br>◯ Spontaneous<br>◯ Adaptive
11.2.1.2.3.1	***If 'Mode of ventilation' is equal to 'Adaptive' answer this question:***<br>Triggered or timed adaptive ventilation<br>*Field type*: Radiobutton<br>*Variable name*: Trig_timed_3<br>*Field required*: Required<br>*Option group name*: Triggered/Timed	◯ Triggered<br>◯ Timed
11.2.1.2.4	***If 'Type of respiratory support' is equal to 'Mechanical ventilation' answer this question:***<br>Tidal volume<br>*Warning shown if field's value is smaller than 100: 'Low value! Please control data input or make a comment.'*<br>*Warning shown if field's value is larger than 900: 'High value! Please control data input or make a comment.'*<br>*Field type*: Numeric field<br>*Variable name*: Tidal_3<br>*Field required*: Required<br>*Field min*: 50<br>*Field max*: 1200<br>*Measurement Unit*: ml	[ ] ml

11.2.1.2.5	***If 'Type of respiratory support' is equal to 'Mechanical ventilation' answer this question:***<br>Total respiratory rate<br>*Warning shown if field's value is smaller than 10: 'Low value! Please control data input or make a comment.'*<br>*Warning shown if field's value is larger than 35: 'High value! Please control data input or make a comment.'*<br>*Field type*: Numeric field<br>*Variable name*: TRR_3<br>*Field required*: Required<br>*Field min*: 4<br>*Field max*: 50<br>*Measurement Unit*: breaths per minut	breaths per minut
11.2.1.2.6	***If 'Type of respiratory support' is equal to 'Mechanical ventilation' answer this question:***<br>PEEP<br>*Warning shown if field's value is smaller than 5: 'Low value! Please control data input or make a comment.'*<br>*Warning shown if field's value is larger than 19: 'High value! Please control data input or make a comment.'*<br>*Field type*: Numeric field<br>*Variable name*: Peep_3<br>*Field required*: Required<br>*Field min*: 0<br>*Field max*: 25<br>*Measurement Unit*: cmH2O	cmH2O
11.2.1.2.3.2	***If 'Mode of ventilation' is not equal to 'Spontaneous' answer this question:***<br>Pplateau<br>*Warning shown if field's value is smaller than or equal to 7: 'Low value! Please control data input or make a comment.'*<br>*Warning shown if field's value is larger than or equal to 30: 'High value! Please control data input or make a comment.'*<br>*Field type*: Numeric field<br>*Variable name*: Pplat_3<br>*Field required*: Required<br>*Field min*: 5<br>*Field max*: 40<br>*Measurement Unit*: cmH2O	cmH2O
11.2.1.2.3.3	***If 'Mode of ventilation' is not equal to 'Spontaneous' answer this question:***<br>Pmax/ Ppeak<br>*Warning shown if field's value is smaller than 10: 'Low value! Please control data input or make a comment.'*<br>*Warning shown if field's value is larger than 29: 'High value! Please control data input or make a comment.'*<br>*Field type*: Numeric field<br>*Variable name*: Pmax_3<br>*Field required*: Required<br>*Field min*: 5<br>*Field max*: 50<br>*Measurement Unit*: cmH2O	cmH2O

11.2.1.2.3.4	***If 'Mode of ventilation' is equal to 'Spontaneous' answer this question:***<br>Pressure support<br>*Warning shown if field's value is smaller than 5: 'Low value! Please control data input or make a comment.'*<br>*Warning shown if field's value is larger than 19: 'High value! Please control data input or make a comment.'*<br>*Field type*: Numeric field<br>*Variable name*: Psup_3<br>*Field required*: Required<br>*Field min*: 0<br>*Field max*: 30<br>*Measurement Unit*: cmH2O	[ ] cmH2O
11.2.1.2.7	***If 'Type of respiratory support' is equal to 'Mechanical ventilation' answer this question:***<br>Unit used for EtCO2, PaO2 and PaCO2<br>*Field type*: Radiobutton<br>*Variable name*: mmhg_kpa_3<br>*Field required*: Required<br>*Option group name*: kPa/mmHg	○ kPa<br>○ mmHg
11.2.1.2.7.1	***If 'Unit used for EtCO2, PaO2 and PaCO2' is equal to 'kPa' answer this question:***<br>EtCO2<br>*Warning shown if field's value is smaller than 2: 'Low value! Please control data input or make a comment.'*<br>*Warning shown if field's value is larger than 8: 'High value! Please control data input or make a comment.'*<br>*Field type*: Numeric field<br>*Variable name*: EtCO2_kpa_3<br>*Field required*: Required<br>*Field min*: 1<br>*Field max*: 20<br>*Measurement Unit*: kPa	[ ] kPa
11.2.1.2.7.2	***If 'Unit used for EtCO2, PaO2 and PaCO2' is equal to 'mmHg' answer this question:***<br>EtCO2<br>*Warning shown if field's value is smaller than 15: 'Low value! Please control data input or make a comment.'*<br>*Warning shown if field's value is larger than 59: 'High value! Please control data input or make a comment.'*<br>*Field type*: Numeric field<br>*Variable name*: EtCO2_mmhg_3<br>*Field required*: Required<br>*Field min*: 7<br>*Field max*: 140<br>*Measurement Unit*: mmHg	[ ] mmHg

11.2.1.2.8	***If 'Type of respiratory support' is equal to 'Mechanical ventilation' answer this question:***<br>FiO2 (closest to AGB, in decimal, e.g. 0.35)<br>*Field type*: Numeric field<br>*Variable name*: FiO2_3<br>*Field required*: Required<br>*Field min*: 0.20999999999999999222284388<br>*Field max*: 1	
11.2.1.2.9	***If 'Type of respiratory support' is equal to 'Mechanical ventilation' answer this question:***<br>SpO2 (closest to AGB)<br>*Notice shown if field's value is smaller than 80: 'Low value! Please control data input or make a comment.'*<br>*Field type*: Numeric field<br>*Variable name*: SpO2_3<br>*Field required*: Required<br>*Field min*: 60<br>*Field max*: 100<br>*Measurement Unit*: %	%
11.2.1.2.10	***If 'Type of respiratory support' is equal to 'Mechanical ventilation' answer this question:***<br>Recruitment maneuvre on this day<br>*Field type*: Radiobutton<br>*Variable name*: Recruit_3<br>*Field required*: Required<br>*Option group name*: Yes/No	◯ Yes<br>◯ No
11.2.1.2.10.1	***If 'Recruitment maneuvre on this day' is equal to 'Yes' answer this question:***<br>Amount of recruitment maneuvres<br>*Warning shown if field's value is larger than 9: 'High value! Please control data input or make a comment'*<br>Times on this day from 00:00 until 23:59.<br>*Field type*: Numeric field<br>*Variable name*: Recruit_times_3<br>*Field required*: Required<br>*Field min*: 0<br>*Field max*: 20<br>*Measurement Unit*: times	times
11.2.1.2.11	***If 'Type of respiratory support' is equal to 'Mechanical ventilation' answer this question:***<br>Prone positioning on this day<br>*Field type*: Radiobutton<br>*Variable name*: Prone_3<br>*Field required*: Required<br>*Option group name*: Yes/No	◯ Yes<br>◯ No

11.2.1.2.11.1 ***If 'Prone positioning on this day' is equal to 'Yes' answer this question:***
Duration of prone posistioning
Hours on this day from 00:00 until 23:59.

Field type: Numeric field
Variable name: Prone_hours_3
Field required: Required
Field min: 0
Field max: 24
Measurement Unit: hours

[ ] hours

11.2.1.2.12 ***If 'Type of respiratory support' is equal to 'Mechanical ventilation' answer this question:***
Do you expect extubation within 48 hours based on the respiration parameters?
If you expect extubation within 48 hours based on the respiration parameters, the ventilation strategy can be abandoned. Please look in our mechanical ventilation handbook for guidelines. When the patient's condition worsens, restart the ventilation strategy according to the protocol.

Field type: Radiobutton
Variable name: ext_3
Field required: Required
Option group name: Yes/No

○ Yes
○ No

ARTERIAL BLOOD GAS AT 6:00

11.2.1.2.14 ***If 'Type of respiratory support' is equal to 'Mechanical ventilation' answer this question:***
Arterial pH
Warning shown if field's value is smaller than 7.0: 'Low value! Please control data input or make a comment.'
Warning shown if field's value is larger than 7.7: 'High value! Please control data input or make a comment.'
Field type: Numeric field
Variable name: ArtpH_3
Field required: Required
Field min: 6.5
Field max: 8
Measurement Unit: pH

[ ] pH

11.2.1.2.15 ***If 'Type of respiratory support' is equal to 'Mechanical ventilation' answer this question:***
Arterial bicarbonate
Warning shown if field's value is smaller than 10: 'Low value! Please control data input or make a comment.'
Warning shown if field's value is larger than 39: 'High value! Please control data input or make a comment.'
Field type: Numeric field
Variable name: Bic_3
Field required: Required
Field min: 2
Field max: 70
Measurement Unit: mmol/l

[ ] mmol/l

11.2.1.2.16	***If 'Type of respiratory support' is equal to 'Mechanical ventilation' answer this question:***<br>Arterial lactate<br>*Warning shown if field's value is larger than 9.9: 'High value! Please control data input or make a comment.'*<br>*Field type*: Numeric field<br>*Variable name*: Lac_3<br>*Field required*: Required<br>*Field min*: 0<br>*Field max*: 25<br>*Measurement Unit*: mmol/L	mmol/L
11.2.1.2.17	***If 'Type of respiratory support' is equal to 'Mechanical ventilation' answer this question:***<br>Arterial saturation<br>*Notice shown if field's value is smaller than 80: 'Low value! Please control data input or make a comment.'*<br>*Field type*: Numeric field<br>*Variable name*: Sat_3<br>*Field required*: Required<br>*Field min*: 60<br>*Field max*: 100<br>*Measurement Unit*: %	%
11.2.1.2.7.3	***If 'Unit used for EtCO2, PaO2 and PaCO2' is equal to 'kPa' answer this question:***<br>Arterial PaO2<br>*Warning shown if field's value is smaller than 7: 'Low value! Please control data input or make a comment.'*<br>*Warning shown if field's value is larger than 18: 'High value! Please control data input or make a comment.'*<br>*Field type*: Numeric field<br>*Variable name*: PaO2_kpa_3<br>*Field required*: Required<br>*Field min*: 4<br>*Field max*: 60<br>*Measurement Unit*: kPa	kPa
11.2.1.2.7.4	***If 'Unit used for EtCO2, PaO2 and PaCO2' is equal to 'mmHg' answer this question:***<br>Arterial PaO2<br>*Warning shown if field's value is smaller than 50: 'Low value! Please control data input or make a comment.'*<br>*Warning shown if field's value is larger than 150: 'High value! Please control data input or make a comment.'*<br>*Field type*: Numeric field<br>*Variable name*: PaO2_mmhg_3<br>*Field required*: Required<br>*Field min*: 28<br>*Field max*: 500<br>*Measurement Unit*: mmHg	mmHg

11.2.1.2.7.5	***If 'Unit used for EtCO2, PaO2 and PaCO2' is equal to 'kPa' answer this question:***<br>Arterial PaCO2<br>*Warning shown if field's value is smaller than 2: 'Low value! Please control data input or make a comment.'*<br>*Warning shown if field's value is larger than 8: 'High value! Please control data input or make a comment.'*<br>*Field type*: Numeric field<br>*Variable name*: PaCO2_kpa_3<br>*Field required*: Required<br>*Field min*: 1<br>*Field max*: 20<br>*Measurement Unit*: kPa	kPa
11.2.1.2.7.6	***If 'Unit used for EtCO2, PaO2 and PaCO2' is equal to 'mmHg' answer this question:***<br>Arterial PaCO2<br>*Warning shown if field's value is smaller than 15: 'Low value! Please control data input or make a comment.'*<br>*Warning shown if field's value is larger than 59: 'High value! Please control data input or make a comment.'*<br>*Field type*: Numeric field<br>*Variable name*: PaCO2_mmhg_3<br>*Field required*: Required<br>*Field min*: 7<br>*Field max*: 140<br>*Measurement Unit*: mmHg	mmHg

12. Daily data - Day 4

Number	Question	Answers
12.1	Date of study day<br>*Field type*: Calculation<br>*Variable name*: date_4<br>*Field required*: Not required	*Template:* var randomization= moment('{castorRandomizedDateAndTime}', 'DD-MM-YYYY'); var newDate = randomization.add(4, 'days'); newDate.format('DD-MM-YYYY');
12.2	***If 'Is the patient hospitalized during any time of the day?' is equal to 'Yes' answer this question:***<br>Is the patient hospitalized during any time of the day?<br>*Notice shown if field's value is equal to No: 'DAILY DATA COMPLETE, GO TO FOLLOW UP.'*<br>*Field type*: Radiobutton<br>*Variable name*: Hospital_patient_4<br>*Field required*: Required<br>*Option group name*: Yes/No	◯ Yes<br>◯ No

12.2.1	***If 'Is the patient hospitalized during any time of the day?' is equal to 'Yes' answer this question:***<br>Location of the patient<br>At 6:00 AM<br><br>*Field type*: Radiobutton<br>*Variable name*: Loc_ICUhosp_4<br>*Field required*: Required<br>*Option group name*: ICU/hospital	◯ Intensive Care Unit<br>◯ Hospital ward
12.2.1.1	***If 'Location of the patient' is equal to 'Hospital ward' answer this question:***<br>Type of respiratory support<br>At 6:00 AM<br><br>*Field type*: Radiobutton<br>*Variable name*: Resp_hospsupp_4<br>*Field required*: Required<br>*Option group name*: resp_supp_hosp	◯ No oxygen therapy<br>◯ Oxygen by mask or nasal prongs<br>◯ High Flow Nasal Oxygen (HFNO) therapy<br>◯ Non-invasive ventilation (NIV)
12.2.1.2	***If 'Location of the patient' is equal to 'Intensive Care Unit' answer this question:***<br>Type of respiratory support<br>At 6:00 AM<br><br>*Field type*: Radiobutton<br>*Variable name*: Resp_supp_4<br>*Field required*: Required<br>*Option group name*: Respiratory support	◯ No oxygen therapy<br>◯ Oxygen by maks or nasal prongs<br>◯ High Flow Nasal Oxygen (HFNO) therapy<br>◯ Non-invasive ventilation (NIV)<br>◯ Mechanical ventilation
12.2.1.3	***If 'Location of the patient' is equal to 'Intensive Care Unit' answer this question:***<br>Daily cumulative fluid balance<br>*Field type*: Radiobutton<br>*Variable name*: Cum_fluid_4<br>*Field required*: Required<br>*Option group name*: Negative/positve	◯ Negative<br>◯ Positive
12.2.1.4	***If 'Location of the patient' is equal to 'Intensive Care Unit' answer this question:***<br>Daily cumulative fluid balance<br>*Warning shown if field's value is larger than or equal to 4000: 'High value! Please control data input or make a comment.'*<br>From 0:00 until 0:00<br><br>*Field type*: Numeric field<br>*Variable name*: Cum_fluid_ml_4<br>*Field required*: Required<br>*Field min*: 0<br>*Field max*: 30000<br>*Measurement Unit*: ml	[ ] ml
	**SOFA SCORE**	

12.2.1.6	***If 'Location of the patient' is equal to 'Intensive Care Unit' answer this question:***<br>PaO2/FiO2 (mmHg (kPa))<br>Worst value of this day. If there is no value for this day please enter missing data.<br><br>*Field type*: Radiobutton<br>*Variable name*: SOFA_Resp_4<br>*Field required*: Required<br>*Option group name*: Respiration	◯ ≥ 400 (53.3)<br>◯ < 400 (53.3)<br>◯ < 300 (40)<br>◯ < 200 (26.7) with respiratory support<br>◯ < 100 (13.3) with respiratory support
12.2.1.7	***If 'Location of the patient' is equal to 'Intensive Care Unit' answer this question:***<br>Platelets (x10^3/μL)<br>Worst value of this day. If there is no value for this day please enter missing data.<br><br>*Field type*: Radiobutton<br>*Variable name*: SOFA_Coag_4<br>*Field required*: Required<br>*Option group name*: Coagulation	◯ ≥ 150<br>◯ < 150<br>◯ < 100<br>◯ < 50<br>◯ < 20
12.2.1.8	***If 'Location of the patient' is equal to 'Intensive Care Unit' answer this question:***<br>Bilirubin (μmol/L(mg/dL))<br>Worst value of this day. If there is no value for this day please enter missing data.<br><br>*Field type*: Radiobutton<br>*Variable name*: SOFA_Live_4<br>*Field required*: Required<br>*Option group name*: Liver	◯ < 20 (< 1.2)<br>◯ 20-32 (1.2-1.9)<br>◯ 33-101 (2.0-5.9)<br>◯ 102-204 (6.0-11.9)<br>◯ > 204 (> 12.0)
12.2.1.9	***If 'Location of the patient' is equal to 'Intensive Care Unit' answer this question:***<br>Cardiovascular<br>Worst value of this day. If there is no value for this day please enter missing data.<br><br>*Field type*: Radiobutton<br>*Variable name*: SOFA_Card_4<br>*Field required*: Required<br>*Option group name*: Cardiovascular	◯ MAP ≥ 70 mm/Hg without dobutamine, epinephrine or norepinephrine<br>◯ MAP < 70 mm/Hg without dobutamine, epinephrine or norepinephrin<br>◯ Dopamine < 5 μg/kg/min or dobutamine (any dose)<br>◯ Dopamine > 5 μg/kg/min OR epinephrine ≤ 0.1 μg/kg/min OR norepinephrine ≤ 0.1 μg/kg/min<br>◯ Dopamine > 15 μg/kg/min OR epinephrine > 0.1 μg/kg/min OR norepinephrine > 0.1 μg/kg/min
12.2.1.10	***If 'Location of the patient' is equal to 'Intensive Care Unit' answer this question:***<br>Glasgow coma scale<br>Only fill in the score when the patient is not sedated. Choose the worst value of this day. If there is no value for this day or the patient is sedated, please enter missing data.<br><br>*Field type*: Radiobutton<br>*Variable name*: SOFA_Nerv_4<br>*Field required*: Required<br>*Option group name*: Nervous	◯ 15<br>◯ 13-14<br>◯ 10-12<br>◯ 6-9<br>◯ < 6

12.2.1.11	***If 'Location of the patient' is equal to 'Intensive Care Unit' answer this question:***<br>Creatinine [or urine output]<br>Worst value of this day. If there is no value for this day please enter missing data.<br><br>*Field type*: Radiobutton<br>*Variable name*: SOFA_Kidn_4<br>*Field required*: Required<br>*Option group name*: Kidneys	◯ < 110 (< 1.2)<br>◯ 110-170 (1.2-1.9)<br>◯ 171-299 (2.0-3.4)<br>◯ 300-440 (3.5-4.9) [or < 500]<br>◯ > 440 (> 5.0) [or < 200]
12.2.1.12	***If 'Location of the patient' is equal to 'Intensive Care Unit' answer this question:***<br>SOFA score<br>*Field type*: Calculation<br>*Variable name*: SOFA_scor_4<br>*Field required*: Not required	*Template:* {SOFA_Resp_4}+{SOFA_Coag_4}+{SOFA_Live_4}+{SOFA_Card_4}+{SOFA_Nerv_4}+{SOFA_Kidn_4}
	**EVENTS**	
12.2.1.14	***If 'Location of the patient' is equal to 'Intensive Care Unit' answer this question:***<br>Did an event occur?<br>• Renal Replacement Therapy during any time of the day.<br>• Placement of a tracheostomy during any time of the day<br>• Use of inhaled vasodilators during any time of the day<br>• Use of airway pressure release ventlation during any time of the day<br>• Use of ECMO during any time of the day<br><br>*Field type*: Checkbox<br>*Variable name*: Event_4<br>*Field required*: Required<br>*Option group name*: Events	☐ None<br>☐ Use of renal replacement therapy<br>☐ Placement of a tracheostomy<br>☐ Use of inhaled vasodilators<br>☐ Use of airway pressure release ventilation<br>☐ Use of ECMO<br>☐ Continuous infusion of neuromuscular blocking agents
12.2.1.15	***If 'Location of the patient' is equal to 'Intensive Care Unit' answer this question:***<br>Calculation for incorrect answer event<br>*Field type*: Calculation<br>*Variable name*: Event_calc_incorrect_4<br>*Field required*: Not required	*Template:* '##allowempty##' var splitted = "{Event_4}".split(';'); if (splitted.indexOf("0") > -1 && (splitted.indexOf("1") > -1 || splitted.indexOf("2") > -1 || splitted.indexOf("3") > -1 || splitted.indexOf("4") > -1 || splitted.indexOf("5") > -1 || splitted.indexOf("6") > -1)) { '1'; } else { '0'; };
	Combination **NOT** possible! **NONE** cannot be chosen when other options are selected!	
	**COMPLICATIONS**	

12.2.1.17	***If 'Location of the patient' is equal to 'Intensive Care Unit' answer this question:***<br>Did a complication occur?<br>Only tick the checkbox at the day of diagnosis.<br>• Pneumothorax: Air in the pleural cavity developed after randomization confirmed by a radiologist on a CT-thorax or chest radiograph for which a drain has been placed.<br>• Ventilator Associated Pneumonia: Clinical Pulmonary Infection Score (CPIS) > 5 with an infiltration on CXR and developed after intubation.<br><br>*Field type*: Checkbox<br>*Variable name*: Compl_4<br>*Field required*: Required<br>*Option group name*: Complications	☐ None<br>☐ Diagnosis of pneumothorax<br>☐ Diagnosis of Ventilator Associated Pneumonia
12.2.1.18	***If 'Location of the patient' is equal to 'Intensive Care Unit' answer this question:***<br>Calculation for incorrect answer complications<br>*Field type*: Calculation<br>*Variable name*: Compl_calc_incorrect_4<br>*Field required*: Not required	*Template:* '##allowempty##' var splitted = "{Compl_4}".split(';'); if (splitted.indexOf("0") > -1 && (splitted.indexOf("1") > -1 || splitted.indexOf("2") > -1 || splitted.indexOf("3") > -1 || splitted.indexOf("4") > -1 || splitted.indexOf("5") > -1 || splitted.indexOf("6") > -1)) { '1'; } else { '0'; };
	Combination **NOT** possible! **NONE** cannot be chosen when other options are selected!	
	**VENTILATION DATA AT 6:00 AM**	
12.2.1.2.2	***If 'Type of respiratory support' is equal to 'Mechanical ventilation' answer this question:***<br>RASS score<br>+4 Combative +3 Very agitated +2 Agitated +1 Restless 0 Alert and calm -1 Drowsy -2 Light sedation -3 Moderate sedation -4 Deep sedation -5 Unarousable<br><br>*Field type*: Radiobutton<br>*Variable name*: Rass_4<br>*Field required*: Required<br>*Option group name*: RASS Score	○ ≥ 0<br>○ - 1<br>○ - 2<br>○ - 3<br>○ - 4<br>○ - 5
12.2.1.2.3	***If 'Type of respiratory support' is equal to 'Mechanical ventilation' answer this question:***<br>Mode of ventilation<br>*Field type*: Radiobutton<br>*Variable name*: Mode_ven_4<br>*Field required*: Required<br>*Option group name*: Mode ventilation	○ Controlled<br>○ Spontaneous<br>○ Adaptive
12.2.1.2.3.1	***If 'Mode of ventilation' is equal to 'Adaptive' answer this question:***<br>Triggered or timed adaptive ventilation<br>*Field type*: Radiobutton<br>*Variable name*: Trig_timed_4<br>*Field required*: Required<br>*Option group name*: Triggered/Timed	○ Triggered<br>○ Timed

12.2.1.2.4	***If 'Type of respiratory support' is equal to 'Mechanical ventilation' answer this question:***<br>Tidal volume<br>*Warning shown if field's value is smaller than 100: 'Low value! Please control data input or make a comment.'*<br>*Warning shown if field's value is larger than 900: 'High value! Please control data input or make a comment.'*<br>*Field type*: Numeric field<br>*Variable name*: Tidal_4<br>*Field required*: Required<br>*Field min*: 50<br>*Field max*: 1200<br>*Measurement Unit*: ml	ml
12.2.1.2.5	***If 'Type of respiratory support' is equal to 'Mechanical ventilation' answer this question:***<br>Total respiratory rate<br>*Warning shown if field's value is smaller than 10: 'Low value! Please control data input or make a comment.'*<br>*Warning shown if field's value is larger than 35: 'High value! Please control data input or make a comment.'*<br>*Field type*: Numeric field<br>*Variable name*: TRR_4<br>*Field required*: Required<br>*Field min*: 4<br>*Field max*: 50<br>*Measurement Unit*: breaths per minut	breaths per minut
12.2.1.2.6	***If 'Type of respiratory support' is equal to 'Mechanical ventilation' answer this question:***<br>PEEP<br>*Warning shown if field's value is smaller than 5: 'Low value! Please control data input or make a comment.'*<br>*Warning shown if field's value is larger than 19: 'High value! Please control data input or make a comment.'*<br>*Field type*: Numeric field<br>*Variable name*: Peep_4<br>*Field required*: Required<br>*Field min*: 0<br>*Field max*: 25<br>*Measurement Unit*: cmH2O	cmH2O
12.2.1.2.3.2	***If 'Mode of ventilation' is not equal to 'Spontaneous' answer this question:***<br>Pplateau<br>*Warning shown if field's value is smaller than or equal to 7: 'Low value! Please control data input or make a comment.'*<br>*Warning shown if field's value is larger than or equal to 30: 'High value! Please control data input or make a comment.'*<br>*Field type*: Numeric field<br>*Variable name*: Pplat_4<br>*Field required*: Required<br>*Field min*: 5<br>*Field max*: 40<br>*Measurement Unit*: cmH2O	cmH2O

12.2.1.2.3.3	***If 'Mode of ventilation' is not equal to 'Spontaneous' answer this question:***<br>Pmax/ Ppeak<br>*Warning shown if field's value is smaller than 10: 'Low value! Please control data input or make a comment.'*<br>*Warning shown if field's value is larger than 29: 'High value! Please control data input or make a comment.'*<br>*Field type*: Numeric field<br>*Variable name*: Pmax_4<br>*Field required*: Required<br>*Field min*: 5<br>*Field max*: 50<br>*Measurement Unit*: cmH2O	[ ] cmH2O
12.2.1.2.3.4	***If 'Mode of ventilation' is equal to 'Spontaneous' answer this question:***<br>Pressure support<br>*Warning shown if field's value is smaller than 5: 'Low value! Please control data input or make a comment.'*<br>*Warning shown if field's value is larger than 19: 'High value! Please control data input or make a comment.'*<br>*Field type*: Numeric field<br>*Variable name*: Psup_4<br>*Field required*: Required<br>*Field min*: 0<br>*Field max*: 30<br>*Measurement Unit*: cmH2O	[ ] cmH2O
12.2.1.2.7	***If 'Type of respiratory support' is equal to 'Mechanical ventilation' answer this question:***<br>Unit used for EtCO2, PaO2 and PaCO2<br>*Field type*: Radiobutton<br>*Variable name*: mmhg_kpa_4<br>*Field required*: Required<br>*Option group name*: kPa/mmHg	○ kPa<br>○ mmHg
12.2.1.2.7.1	***If 'Unit used for EtCO2, PaO2 and PaCO2' is equal to 'kPa' answer this question:***<br>EtCO2<br>*Warning shown if field's value is smaller than 2: 'Low value! Please control data input or make a comment.'*<br>*Warning shown if field's value is larger than 8: 'High value! Please control data input or make a comment.'*<br>*Field type*: Numeric field<br>*Variable name*: EtCO2_kpa_4<br>*Field required*: Required<br>*Field min*: 1<br>*Field max*: 20<br>*Measurement Unit*: kPa	[ ] kPa

12.2.1.2.7.2	***If 'Unit used for EtCO2, PaO2 and PaCO2' is equal to 'mmHg' answer this question:***<br>EtCO2<br>*Warning shown if field's value is smaller than 15: 'Low value! Please control data input or make a comment.'*<br>*Warning shown if field's value is larger than 59: 'High value! Please control data input or make a comment.'*<br>*Field type*: Numeric field<br>*Variable name*: EtCO2_mmhg_4<br>*Field required*: Required<br>*Field min*: 7<br>*Field max*: 140<br>*Measurement Unit*: mmHg	[ ] mmHg
12.2.1.2.8	***If 'Type of respiratory support' is equal to 'Mechanical ventilation' answer this question:***<br>FiO2 (closest to AGB, in decimal, e.g. 0.35)<br>*Field type*: Numeric field<br>*Variable name*: FiO2_4<br>*Field required*: Required<br>*Field min*: 0.2099999999999999922284388<br>*Field max*: 1	[ ]
12.2.1.2.9	***If 'Type of respiratory support' is equal to 'Mechanical ventilation' answer this question:***<br>SpO2 (closest to AGB)<br>*Notice shown if field's value is smaller than 80: 'Low value! Please control data input or make a comment.'*<br>*Field type*: Numeric field<br>*Variable name*: SpO2_4<br>*Field required*: Required<br>*Field min*: 60<br>*Field max*: 100<br>*Measurement Unit*: %	[ ] %
12.2.1.2.10	***If 'Type of respiratory support' is equal to 'Mechanical ventilation' answer this question:***<br>Recruitment maneuvre on this day<br>*Field type*: Radiobutton<br>*Variable name*: Recruit_4<br>*Field required*: Required<br>*Option group name*: Yes/No	○ Yes<br>○ No
12.2.1.2.10.1	***If 'Recruitment maneuvre on this day' is equal to 'Yes' answer this question:***<br>Amount of recruitment maneuvres<br>*Warning shown if field's value is larger than 9: 'High value! Please control data input or make a comment'*<br>Times on this day from 00:00 until 23:59.<br>*Field type*: Numeric field<br>*Variable name*: Recruit_times_4<br>*Field required*: Required<br>*Field min*: 0<br>*Field max*: 20<br>*Measurement Unit*: times	[ ] times

12.2.1.2.11	***If 'Type of respiratory support' is equal to 'Mechanical ventilation' answer this question:***<br>Prone positioning on this day<br>*Field type*: Radiobutton<br>*Variable name*: Prone_4<br>*Field required*: Required<br>*Option group name*: Yes/No	◯ Yes<br>◯ No
12.2.1.2.11.1	***If 'Prone positioning on this day' is equal to 'Yes' answer this question:***<br>Duration of prone posistioning<br>Hours on this day from 00:00 until 23:59.<br><br>*Field type*: Numeric field<br>*Variable name*: Prone_hours_4<br>*Field required*: Required<br>*Field min*: 0<br>*Field max*: 24<br>*Measurement Unit*: hours	[ ] hours
12.2.1.2.12	***If 'Type of respiratory support' is equal to 'Mechanical ventilation' answer this question:***<br>Do you expect extubation within 48 hours based on the respiration parameters?<br>If you expect extubation within 48 hours based on the respiration parameters, the ventilation strategy can be abandoned. Please look in our mechanical ventilation handbook for guidelines. When the patient's condition worsens, restart the ventilation strategy according to the protocol.<br><br>*Field type*: Radiobutton<br>*Variable name*: ext_4<br>*Field required*: Required<br>*Option group name*: Yes/No	◯ Yes<br>◯ No
	**ARTERIAL BLOOD GAS AT 6:00**	
12.2.1.2.14	***If 'Type of respiratory support' is equal to 'Mechanical ventilation' answer this question:***<br>Arterial pH<br>*Warning shown if field's value is smaller than 7.0: 'Low value! Please control data input or make a comment.'*<br>*Warning shown if field's value is larger than 7.7: 'High value! Please control data input or make a comment.'*<br>*Field type*: Numeric field<br>*Variable name*: ArtpH_4<br>*Field required*: Required<br>*Field min*: 6.5<br>*Field max*: 8<br>*Measurement Unit*: pH	[ ] pH

12.2.1.2.15	***If 'Type of respiratory support' is equal to 'Mechanical ventilation' answer this question:***<br>Arterial bicarbonate<br>*Warning shown if field's value is smaller than 10: 'Low value! Please control data input or make a comment.'*<br>*Warning shown if field's value is larger than 39: 'High value! Please control data input or make a comment.'*<br>*Field type*: Numeric field<br>*Variable name*: Bic_4<br>*Field required*: Required<br>*Field min*: 2<br>*Field max*: 70<br>*Measurement Unit*: mmol/l	[ ] mmol/l
12.2.1.2.16	***If 'Type of respiratory support' is equal to 'Mechanical ventilation' answer this question:***<br>Arterial lactate<br>*Warning shown if field's value is larger than 9.9: 'High value! Please control data input or make a comment.'*<br>*Field type*: Numeric field<br>*Variable name*: Lac_4<br>*Field required*: Required<br>*Field min*: 0<br>*Field max*: 25<br>*Measurement Unit*: mmol/L	[ ] mmol/L
12.2.1.2.17	***If 'Type of respiratory support' is equal to 'Mechanical ventilation' answer this question:***<br>Arterial saturation<br>*Notice shown if field's value is smaller than 80: 'Low value! Please control data input or make a comment.'*<br>*Field type*: Numeric field<br>*Variable name*: Sat_4<br>*Field required*: Required<br>*Field min*: 60<br>*Field max*: 100<br>*Measurement Unit*: %	[ ] %
12.2.1.2.7.3	***If 'Unit used for EtCO2, PaO2 and PaCO2' is equal to 'kPa' answer this question:***<br>Arterial PaO2<br>*Warning shown if field's value is smaller than 7: 'Low value! Please control data input or make a comment.'*<br>*Warning shown if field's value is larger than 18: 'High value! Please control data input or make a comment.'*<br>*Field type*: Numeric field<br>*Variable name*: PaO2_kpa_4<br>*Field required*: Required<br>*Field min*: 4<br>*Field max*: 60<br>*Measurement Unit*: kPa	[ ] kPa

12.2.1.2.7.4	***If 'Unit used for EtCO2, PaO2 and PaCO2' is equal to 'mmHg' answer this question:***<br>Arterial PaO2<br>*Warning shown if field's value is smaller than 50: 'Low value! Please control data input or make a comment.'*<br>*Warning shown if field's value is larger than 150: 'High value! Please control data input or make a comment.'*<br>*Field type*: Numeric field<br>*Variable name*: PaO2_mmhg_4<br>*Field required*: Required<br>*Field min*: 28<br>*Field max*: 500<br>*Measurement Unit*: mmHg	[ ] mmHg
12.2.1.2.7.5	***If 'Unit used for EtCO2, PaO2 and PaCO2' is equal to 'kPa' answer this question:***<br>Arterial PaCO2<br>*Warning shown if field's value is smaller than 2: 'Low value! Please control data input or make a comment.'*<br>*Warning shown if field's value is larger than 8: 'High value! Please control data input or make a comment.'*<br>*Field type*: Numeric field<br>*Variable name*: PaCO2_kpa_4<br>*Field required*: Required<br>*Field min*: 1<br>*Field max*: 20<br>*Measurement Unit*: kPa	[ ] kPa
12.2.1.2.7.6	***If 'Unit used for EtCO2, PaO2 and PaCO2' is equal to 'mmHg' answer this question:***<br>Arterial PaCO2<br>*Warning shown if field's value is smaller than 15: 'Low value! Please control data input or make a comment.'*<br>*Warning shown if field's value is larger than 59: 'High value! Please control data input or make a comment.'*<br>*Field type*: Numeric field<br>*Variable name*: PaCO2_mmhg_4<br>*Field required*: Required<br>*Field min*: 7<br>*Field max*: 140<br>*Measurement Unit*: mmHg	[ ] mmHg

13. Daily data - Day 5

Number	Question	Answers
13.1	Date of study day<br>*Field type*: Calculation<br>*Variable name*: date_5<br>*Field required*: Not required	*Template:* var randomization= moment('{castorRandomizedDateAndTime}', 'DD-MM-YYYY'); var newDate = randomization.add(5, 'days'); newDate.format('DD-MM-YYYY');

13.2	***If 'Is the patient hospitalized during any time of the day?' is equal to 'Yes' answer this question:***<br>Is the patient hospitalized during any time of the day?<br>*Notice shown if field's value is equal to No: 'DAILY DATA COMPLETE, GO TO FOLLOW UP.'*<br>*Field type*: Radiobutton<br>*Variable name*: Hospital_patient_5<br>*Field required*: Required<br>*Option group name*: Yes/No	◯ Yes<br>◯ No
13.2.1	***If 'Is the patient hospitalized during any time of the day?' is equal to 'Yes' answer this question:***<br>Location of the patient<br>At 6:00 AM<br>*Field type*: Radiobutton<br>*Variable name*: Loc_ICUhosp_5<br>*Field required*: Required<br>*Option group name*: ICU/hospital	◯ Intensive Care Unit<br>◯ Hospital ward
13.2.1.1	***If 'Location of the patient' is equal to 'Hospital ward' answer this question:***<br>Type of respiratory support<br>At 6:00 AM<br>*Field type*: Radiobutton<br>*Variable name*: Resp_hospsupp_5<br>*Field required*: Required<br>*Option group name*: resp_supp_hosp	◯ No oxygen therapy<br>◯ Oxygen by mask or nasal prongs<br>◯ High Flow Nasal Oxygen (HFNO) therapy<br>◯ Non-invasive ventilation (NIV)
13.2.1.2	***If 'Location of the patient' is equal to 'Intensive Care Unit' answer this question:***<br>Type of respiratory support<br>At 6:00 AM<br>*Field type*: Radiobutton<br>*Variable name*: Resp_supp_5<br>*Field required*: Required<br>*Option group name*: Respiratory support	◯ No oxygen therapy<br>◯ Oxygen by maks or nasal prongs<br>◯ High Flow Nasal Oxygen (HFNO) therapy<br>◯ Non-invasive ventilation (NIV)<br>◯ Mechanical ventilation
13.2.1.3	***If 'Location of the patient' is equal to 'Intensive Care Unit' answer this question:***<br>Daily cumulative fluid balance<br>*Field type*: Radiobutton<br>*Variable name*: Cum_fluid_5<br>*Field required*: Required<br>*Option group name*: Negative/positve	◯ Negative<br>◯ Positive

13.2.1.4	***If 'Location of the patient' is equal to 'Intensive Care Unit' answer this question:***<br>Daily cumulative fluid balance<br>*Warning shown if field's value is larger than or equal to 4000: 'High value! Please control data input or make a comment.'*<br>From 0:00 until 0:00<br><br>*Field type*: Numeric field<br>*Variable name*: Cum_fluid_ml_5<br>*Field required*: Required<br>*Field min*: 0<br>*Field max*: 30000<br>*Measurement Unit*: ml	ml
	**SOFA SCORE**	
13.2.1.6	***If 'Location of the patient' is equal to 'Intensive Care Unit' answer this question:***<br>PaO2/FiO2 (mmHg (kPa))<br>Worst value of this day. If there is no value for this day please enter missing data.<br><br>*Field type*: Radiobutton<br>*Variable name*: SOFA_Resp_5<br>*Field required*: Required<br>*Option group name*: Respiration	◯ ≥ 400 (53.3)<br>◯ < 400 (53.3)<br>◯ < 300 (40)<br>◯ < 200 (26.7) with respiratory support<br>◯ < 100 (13.3) with respiratory support
13.2.1.7	***If 'Location of the patient' is equal to 'Intensive Care Unit' answer this question:***<br>Platelets (x10^3/µL)<br>Worst value of this day. If there is no value for this day please enter missing data.<br><br>*Field type*: Radiobutton<br>*Variable name*: SOFA_Coag_5<br>*Field required*: Required<br>*Option group name*: Coagulation	◯ ≥ 150<br>◯ < 150<br>◯ < 100<br>◯ < 50<br>◯ < 20
13.2.1.8	***If 'Location of the patient' is equal to 'Intensive Care Unit' answer this question:***<br>Bilirubin (µmol/L(mg/dL))<br>Worst value of this day. If there is no value for this day please enter missing data.<br><br>*Field type*: Radiobutton<br>*Variable name*: SOFA_Live_5<br>*Field required*: Required<br>*Option group name*: Liver	◯ < 20 (< 1.2)<br>◯ 20-32 (1.2-1.9)<br>◯ 33-101 (2.0-5.9)<br>◯ 102-204 (6.0-11.9)<br>◯ > 204 (> 12.0)

13.2.1.9	***If 'Location of the patient' is equal to 'Intensive Care Unit' answer this question:***<br>Cardiovascular<br>Worst value of this day. If there is no value for this day please enter missing data.<br><br>*Field type*: Radiobutton<br>*Variable name*: SOFA_Card_5<br>*Field required*: Required<br>*Option group name*: Cardiovascular	◯ MAP ≥ 70 mm/Hg without dobutamine, epinephrine or norepinephrine<br>◯ MAP < 70 mm/Hg without dobutamine, epinephrine or norepinephrin<br>◯ Dopamine < 5 µg/kg/min or dobutamine (any dose)<br>◯ Dopamine > 5 µg/kg/min OR epinephrine ≤ 0.1 µg/kg/min OR norepinephrine ≤ 0.1 µg/kg/min<br>◯ Dopamine > 15 µg/kg/min OR epinephrine > 0.1 µg/kg/min OR norepinephrine > 0.1 µg/kg/min
13.2.1.10	***If 'Location of the patient' is equal to 'Intensive Care Unit' answer this question:***<br>Glasgow coma scale<br>Only fill in the score when the patient is not sedated. Choose the worst value of this day. If there is no value for this day or the patient is sedated, please enter missing data.<br><br>*Field type*: Radiobutton<br>*Variable name*: SOFA_Nerv_5<br>*Field required*: Required<br>*Option group name*: Nervous	◯ 15<br>◯ 13-14<br>◯ 10-12<br>◯ 6-9<br>◯ < 6
13.2.1.11	***If 'Location of the patient' is equal to 'Intensive Care Unit' answer this question:***<br>Creatinine [or urine output]<br>Worst value of this day. If there is no value for this day please enter missing data.<br><br>*Field type*: Radiobutton<br>*Variable name*: SOFA_Kidn_5<br>*Field required*: Required<br>*Option group name*: Kidneys	◯ < 110 (< 1.2)<br>◯ 110-170 (1.2-1.9)<br>◯ 171-299 (2.0-3.4)<br>◯ 300-440 (3.5-4.9) [or < 500]<br>◯ > 440 (> 5.0) [or < 200]
13.2.1.12	***If 'Location of the patient' is equal to 'Intensive Care Unit' answer this question:***<br>SOFA score<br>*Field type*: Calculation<br>*Variable name*: SOFA_scor_5<br>*Field required*: Not required	*Template:* {SOFA_Resp_5}+{SOFA_Coag_5}+{SOFA_Live_5}+{SOFA_Card_5}+{SOFA_Nerv_5}+{SOFA_Kidn_5}
	**EVENTS**	

13.2.1.14	***If 'Location of the patient' is equal to 'Intensive Care Unit' answer this question:***<br>Did an event occur?<br>• Renal Replacement Therapy during any time of the day.<br>• Placement of a tracheostomy during any time of the day<br>• Use of inhaled vasodilators during any time of the day<br>• Use of airway pressure release ventlation during any time of the day<br>• Use of ECMO during any time of the day<br><br>*Field type*: Checkbox<br>*Variable name*: Event_5<br>*Field required*: Required<br>*Option group name*: Events	☐ None<br>☐ Use of renal replacement therapy<br>☐ Placement of a tracheostomy<br>☐ Use of inhaled vasodilators<br>☐ Use of airway pressure release ventilation<br>☐ Use of ECMO<br>☐ Continuous infusion of neuromuscular blocking agents
13.2.1.15	***If 'Location of the patient' is equal to 'Intensive Care Unit' answer this question:***<br>Calculation for incorrect answer event<br>*Field type*: Calculation<br>*Variable name*: Event_calc_incorrect_5<br>*Field required*: Not required	*Template:* '##allowempty##' var splitted = "{Event_5}".split(';'); if (splitted.indexOf("0") > -1 && (splitted.indexOf("1") > -1 || splitted.indexOf("2") > -1 || splitted.indexOf("3") > -1 || splitted.indexOf("4") > -1 || splitted.indexOf("5") > -1 || splitted.indexOf("6") > -1)) { '1'; } else { '0'; };
	Combination **NOT** possible! **NONE** cannot be chosen when other options are selected!	
	**COMPLICATIONS**	
13.2.1.17	***If 'Location of the patient' is equal to 'Intensive Care Unit' answer this question:***<br>Did a complication occur?<br>Only tick the checkbox at the day of diagnosis.<br>• Pneumothorax: Air in the pleural cavity developed after randomization confirmed by a radiologist on a CT-thorax or chest radiograph for which a drain has been placed.<br>• Ventilator Associated Pneumonia: Clinical Pulmonary Infection Score (CPIS) > 5 with an infiltration on CXR and developed after intubation.<br><br>*Field type*: Checkbox<br>*Variable name*: Compl_5<br>*Field required*: Required<br>*Option group name*: Complications	☐ None<br>☐ Diagnosis of pneumothorax<br>☐ Diagnosis of Ventilator Associated Pneumonia
13.2.1.18	***If 'Location of the patient' is equal to 'Intensive Care Unit' answer this question:***<br>Calculation for incorrect answer complications<br>*Field type*: Calculation<br>*Variable name*: Compl_calc_incorrect_5<br>*Field required*: Not required	*Template:* '##allowempty##' var splitted = "{Compl_5}".split(';'); if (splitted.indexOf("0") > -1 && (splitted.indexOf("1") > -1 || splitted.indexOf("2") > -1 || splitted.indexOf("3") > -1 || splitted.indexOf("4") > -1 || splitted.indexOf("5") > -1 || splitted.indexOf("6") > -1)) { '1'; } else { '0'; };
	Combination **NOT** possible! **NONE** cannot be chosen when other options are selected!	

VENTILATION DATA AT 6:00 AM

13.2.1.2.2	***If 'Type of respiratory support' is equal to 'Mechanical ventilation' answer this question:***<br>RASS score<br>+4 Combative +3 Very agitated +2 Agitated +1 Restless 0 Alert and calm -1 Drowsy -2 Light sedation -3 Moderate sedation -4 Deep sedation -5 Unarousable<br>*Field type*: Radiobutton<br>*Variable name*: Rass_5<br>*Field required*: Required<br>*Option group name*: RASS Score	◯ ≥ 0<br>◯ - 1<br>◯ - 2<br>◯ - 3<br>◯ - 4<br>◯ - 5
13.2.1.2.3	***If 'Type of respiratory support' is equal to 'Mechanical ventilation' answer this question:***<br>Mode of ventilation<br>*Field type*: Radiobutton<br>*Variable name*: Mode_ven_5<br>*Field required*: Required<br>*Option group name*: Mode ventilation	◯ Controlled<br>◯ Spontaneous<br>◯ Adaptive
13.2.1.2.3.1	***If 'Mode of ventilation' is equal to 'Adaptive' answer this question:***<br>Triggered or timed adaptive ventilation<br>*Field type*: Radiobutton<br>*Variable name*: Trig_timed_5<br>*Field required*: Required<br>*Option group name*: Triggered/Timed	◯ Triggered<br>◯ Timed
13.2.1.2.4	***If 'Type of respiratory support' is equal to 'Mechanical ventilation' answer this question:***<br>Tidal volume<br>*Warning shown if field's value is smaller than 100: 'Low value! Please control data input or make a comment.'*<br>*Warning shown if field's value is larger than 900: 'High value! Please control data input or make a comment.'*<br>*Field type*: Numeric field<br>*Variable name*: Tidal_5<br>*Field required*: Required<br>*Field min*: 50<br>*Field max*: 1200<br>*Measurement Unit*: ml	[ ] ml

13.2.1.2.5	***If 'Type of respiratory support' is equal to 'Mechanical ventilation' answer this question:***<br>Total respiratory rate<br>*Warning shown if field's value is smaller than 10: 'Low value! Please control data input or make a comment.'*<br>*Warning shown if field's value is larger than 35: 'High value! Please control data input or make a comment.'*<br>*Field type*: Numeric field<br>*Variable name*: TRR_5<br>*Field required*: Required<br>*Field min*: 4<br>*Field max*: 50<br>*Measurement Unit*: breaths per minut	breaths per minut
13.2.1.2.6	***If 'Type of respiratory support' is equal to 'Mechanical ventilation' answer this question:***<br>PEEP<br>*Warning shown if field's value is smaller than 5: 'Low value! Please control data input or make a comment.'*<br>*Warning shown if field's value is larger than 19: 'High value! Please control data input or make a comment.'*<br>*Field type*: Numeric field<br>*Variable name*: Peep_5<br>*Field required*: Required<br>*Field min*: 0<br>*Field max*: 25<br>*Measurement Unit*: cmH2O	cmH2O
13.2.1.2.3.2	***If 'Mode of ventilation' is not equal to 'Spontaneous' answer this question:***<br>Pplateau<br>*Warning shown if field's value is smaller than or equal to 7: 'Low value! Please control data input or make a comment.'*<br>*Warning shown if field's value is larger than or equal to 30: 'High value! Please control data input or make a comment.'*<br>*Field type*: Numeric field<br>*Variable name*: Pplat_5<br>*Field required*: Required<br>*Field min*: 5<br>*Field max*: 40<br>*Measurement Unit*: cmH2O	cmH2O
13.2.1.2.3.3	***If 'Mode of ventilation' is not equal to 'Spontaneous' answer this question:***<br>Pmax/ Ppeak<br>*Warning shown if field's value is smaller than 10: 'Low value! Please control data input or make a comment.'*<br>*Warning shown if field's value is larger than 29: 'High value! Please control data input or make a comment.'*<br>*Field type*: Numeric field<br>*Variable name*: Pmax_5<br>*Field required*: Required<br>*Field min*: 5<br>*Field max*: 50<br>*Measurement Unit*: cmH2O	cmH2O

13.2.1.2.3.4	***If 'Mode of ventilation' is equal to 'Spontaneous' answer this question:***<br>Pressure support<br>*Warning shown if field's value is smaller than 5: 'Low value! Please control data input or make a comment.'*<br>*Warning shown if field's value is larger than 19: 'High value! Please control data input or make a comment.'*<br>*Field type*: Numeric field<br>*Variable name*: Psup_5<br>*Field required*: Required<br>*Field min*: 0<br>*Field max*: 30<br>*Measurement Unit*: cmH2O	[ ] cmH2O
13.2.1.2.7	***If 'Type of respiratory support' is equal to 'Mechanical ventilation' answer this question:***<br>Unit used for EtCO2, PaO2 and PaCO2<br>*Field type*: Radiobutton<br>*Variable name*: mmhg_kpa_5<br>*Field required*: Required<br>*Option group name*: kPa/mmHg	○ kPa<br>○ mmHg
13.2.1.2.7.1	***If 'Unit used for EtCO2, PaO2 and PaCO2' is equal to 'kPa' answer this question:***<br>EtCO2<br>*Warning shown if field's value is smaller than 2: 'Low value! Please control data input or make a comment.'*<br>*Warning shown if field's value is larger than 8: 'High value! Please control data input or make a comment.'*<br>*Field type*: Numeric field<br>*Variable name*: EtCO2_kpa_5<br>*Field required*: Required<br>*Field min*: 1<br>*Field max*: 20<br>*Measurement Unit*: kPa	[ ] kPa
13.2.1.2.7.2	***If 'Unit used for EtCO2, PaO2 and PaCO2' is equal to 'mmHg' answer this question:***<br>EtCO2<br>*Warning shown if field's value is smaller than 15: 'Low value! Please control data input or make a comment.'*<br>*Warning shown if field's value is larger than 59: 'High value! Please control data input or make a comment.'*<br>*Field type*: Numeric field<br>*Variable name*: EtCO2_mmhg_5<br>*Field required*: Required<br>*Field min*: 7<br>*Field max*: 140<br>*Measurement Unit*: mmHg	[ ] mmHg

13.2.1.2.8 ***If 'Type of respiratory support' is equal to 'Mechanical ventilation' answer this question:***
FiO2 (closest to AGB, in decimal, e.g. 0.35)
Field type: Numeric field
Variable name: FiO2_5
Field required: Required
Field min: 0.2099999999999999922284388
Field max: 1

13.2.1.2.9 ***If 'Type of respiratory support' is equal to 'Mechanical ventilation' answer this question:***
SpO2 (closest to AGB)
Notice shown if field's value is smaller than 80: 'Low value! Please control data input or make a comment.'
Field type: Numeric field
Variable name: SpO2_5
Field required: Required
Field min: 60
Field max: 100
Measurement Unit: %

[ ] %

13.2.1.2.10 ***If 'Type of respiratory support' is equal to 'Mechanical ventilation' answer this question:***
Recruitment maneuvre on this day
Times on this day from 00:00 until 23:59.

Field type: Radiobutton
Variable name: Recruit_5
Field required: Required
Option group name: Yes/No

○ Yes
○ No

13.2.1.2.10.1 ***If 'Recruitment maneuvre on this day' is equal to 'Yes' answer this question:***
Amount of recruitment maneuvres
Warning shown if field's value is larger than 9: 'High value! Please control data input or make a comment'
Field type: Numeric field
Variable name: Recruit_times_5
Field required: Required
Field min: 0
Field max: 20
Measurement Unit: times

[ ] times

13.2.1.2.11 ***If 'Type of respiratory support' is equal to 'Mechanical ventilation' answer this question:***
Prone positioning on this day
Field type: Radiobutton
Variable name: Prone_5
Field required: Required
Option group name: Yes/No

○ Yes
○ No

13.2.1.2.11.1 ***If 'Prone positioning on this day' is equal to 'Yes' answer this question:***
Duration of prone posistioning
Hours on this day from 00:00 until 23:59.

Field type: Numeric field
Variable name: Prone_hours_5
Field required: Required
Field min: 0
Field max: 24
Measurement Unit: hours

[ ] hours

13.2.1.2.12 ***If 'Type of respiratory support' is equal to 'Mechanical ventilation' answer this question:***
Do you expect extubation within 48 hours based on the respiration parameters?
If you expect extubation within 48 hours based on the respiration parameters, the ventilation strategy can be abandoned. Please look in our mechanical ventilation handbook for guidelines. When the patient's condition worsens, restart the ventilation strategy according to the protocol.

Field type: Radiobutton
Variable name: ext_5
Field required: Required
Option group name: Yes/No

○ Yes
○ No

ARTERIAL BLOOD GAS AT 6:00

13.2.1.2.14 ***If 'Type of respiratory support' is equal to 'Mechanical ventilation' answer this question:***
Arterial pH
Warning shown if field's value is smaller than 7.0: 'Low value! Please control data input or make a comment.'
Warning shown if field's value is larger than 7.7: 'High value! Please control data input or make a comment.'
Field type: Numeric field
Variable name: ArtpH_5
Field required: Required
Field min: 6.5
Field max: 8
Measurement Unit: pH

[ ] pH

13.2.1.2.15 ***If 'Type of respiratory support' is equal to 'Mechanical ventilation' answer this question:***
Arterial bicarbonate
Warning shown if field's value is smaller than 10: 'Low value! Please control data input or make a comment.'
Warning shown if field's value is larger than 39: 'High value! Please control data input or make a comment.'
Field type: Numeric field
Variable name: Bic_5
Field required: Required
Field min: 2
Field max: 70
Measurement Unit: mmol/l

[ ] mmol/l

13.2.1.2.16	***If 'Type of respiratory support' is equal to 'Mechanical ventilation' answer this question:***<br>Arterial lactate<br>*Warning shown if field's value is larger than 9.9: 'High value! Please control data input or make a comment.'*<br>*Field type*: Numeric field<br>*Variable name*: Lac_5<br>*Field required*: Required<br>*Field min*: 0<br>*Field max*: 25<br>*Measurement Unit*: mmol/L	mmol/L
13.2.1.2.17	***If 'Type of respiratory support' is equal to 'Mechanical ventilation' answer this question:***<br>Arterial saturation<br>*Notice shown if field's value is smaller than 80: 'Low value! Please control data input or make a comment.'*<br>*Field type*: Numeric field<br>*Variable name*: Sat_5<br>*Field required*: Required<br>*Field min*: 60<br>*Field max*: 100<br>*Measurement Unit*: %	%
13.2.1.2.7.3	***If 'Unit used for EtCO2, PaO2 and PaCO2' is equal to 'kPa' answer this question:***<br>Arterial PaO2<br>*Warning shown if field's value is smaller than 7: 'Low value! Please control data input or make a comment.'*<br>*Warning shown if field's value is larger than 18: 'High value! Please control data input or make a comment.'*<br>*Field type*: Numeric field<br>*Variable name*: PaO2_kpa_5<br>*Field required*: Required<br>*Field min*: 4<br>*Field max*: 60<br>*Measurement Unit*: kPa	kPa
13.2.1.2.7.4	***If 'Unit used for EtCO2, PaO2 and PaCO2' is equal to 'mmHg' answer this question:***<br>Arterial PaO2<br>*Warning shown if field's value is smaller than 50: 'Low value! Please control data input or make a comment.'*<br>*Warning shown if field's value is larger than 150: 'High value! Please control data input or make a comment.'*<br>*Field type*: Numeric field<br>*Variable name*: PaO2_mmhg_5<br>*Field required*: Required<br>*Field min*: 28<br>*Field max*: 500<br>*Measurement Unit*: mmHg	mmHg

13.2.1.2.7.5	***If 'Unit used for EtCO2, PaO2 and PaCO2' is equal to 'kPa' answer this question:***<br>Arterial PaCO2<br>*Warning shown if field's value is smaller than 2: 'Low value! Please control data input or make a comment.'*<br>*Warning shown if field's value is larger than 8: 'High value! Please control data input or make a comment.'*<br>*Field type*: Numeric field<br>*Variable name*: PaCO2_kpa_5<br>*Field required*: Required<br>*Field min*: 1<br>*Field max*: 20<br>*Measurement Unit*: kPa	kPa
13.2.1.2.7.6	***If 'Unit used for EtCO2, PaO2 and PaCO2' is equal to 'mmHg' answer this question:***<br>Arterial PaCO2<br>*Warning shown if field's value is smaller than 15: 'Low value! Please control data input or make a comment.'*<br>*Warning shown if field's value is larger than 59: 'High value! Please control data input or make a comment.'*<br>*Field type*: Numeric field<br>*Variable name*: PaCO2_mmhg_5<br>*Field required*: Required<br>*Field min*: 7<br>*Field max*: 140<br>*Measurement Unit*: mmHg	mmHg

14. Daily data - Day 6

Number	Question	Answers
14.1	Date of study day<br>*Field type*: Calculation<br>*Variable name*: date_6<br>*Field required*: Not required	*Template:* var randomization= moment('{castorRandomizedDateAndTime}', 'DD-MM-YYYY'); var newDate = randomization.add(6, 'days'); newDate.format('DD-MM-YYYY');
14.2	***If 'Is the patient hospitalized during any time of the day?' is equal to 'Yes' answer this question:***<br>Is the patient hospitalized during any time of the day?<br>*Notice shown if field's value is equal to No: 'DAILY DATA COMPLETE, GO TO FOLLOW UP.'*<br>*Field type*: Radiobutton<br>*Variable name*: Hospital_patient_6<br>*Field required*: Required<br>*Option group name*: Yes/No	◯ Yes<br>◯ No

14.2.1	***If 'Is the patient hospitalized during any time of the day?' is equal to 'Yes' answer this question:***<br>Location of the patient<br>At 6:00 AM<br><br>*Field type*: Radiobutton<br>*Variable name*: Loc_ICUhosp_6<br>*Field required*: Required<br>*Option group name*: ICU/hospital	○ Intensive Care Unit<br>○ Hospital ward
14.2.1.1	***If 'Location of the patient' is equal to 'Hospital ward' answer this question:***<br>Type of respiratory support<br>At 6:00 AM<br><br>*Field type*: Radiobutton<br>*Variable name*: Resp_hospsupp_6<br>*Field required*: Required<br>*Option group name*: resp_supp_hosp	○ No oxygen therapy<br>○ Oxygen by mask or nasal prongs<br>○ High Flow Nasal Oxygen (HFNO) therapy<br>○ Non-invasive ventilation (NIV)
14.2.1.2	***If 'Location of the patient' is equal to 'Intensive Care Unit' answer this question:***<br>Type of respiratory support<br>At 6:00 AM<br><br>*Field type*: Radiobutton<br>*Variable name*: Resp_supp_6<br>*Field required*: Required<br>*Option group name*: Respiratory support	○ No oxygen therapy<br>○ Oxygen by maks or nasal prongs<br>○ High Flow Nasal Oxygen (HFNO) therapy<br>○ Non-invasive ventilation (NIV)<br>○ Mechanical ventilation
14.2.1.3	***If 'Location of the patient' is equal to 'Intensive Care Unit' answer this question:***<br>Daily cumulative fluid balance<br>*Field type*: Radiobutton<br>*Variable name*: Cum_fluid_6<br>*Field required*: Required<br>*Option group name*: Negative/positve	○ Negative<br>○ Positive
14.2.1.4	***If 'Location of the patient' is equal to 'Intensive Care Unit' answer this question:***<br>Daily cumulative fluid balance<br>*Warning shown if field's value is larger than or equal to 4000: 'High value! Please control data input or make a comment.'*<br>From 0:00 until 0:00<br><br>*Field type*: Numeric field<br>*Variable name*: Cum_fluid_ml_6<br>*Field required*: Required<br>*Field min*: 0<br>*Field max*: 30000<br>*Measurement Unit*: ml	[ ] ml
14.3	Calculation FU LUS<br>*Field type*: Calculation<br>*Variable name*: Cal_FU_LUS_1<br>*Field required*: Not required	*Template:* if ({Confirm_LUS_1} == 0 && {Conf_rando} == 0 && {Resp_supp_6} == 4) { '0' ; } else { '1' ; }

	**Perform a Follow up LUS exam**	
14.3.2	***If 'Calculation FU LUS' is equal to '0' answer this question:***<br>LUS exam performed by<br>*Field type*: Textfield<br>*Variable name*: LUS_performed_2<br>*Field required*: Required	[ ]
14.3.3	***If 'Calculation FU LUS' is equal to '0' answer this question:***<br>PEEP during LUS exam<br>*Warning shown if field's value is larger than 8: 'LUS exam should be performed with a PEEP level of 5 cmH2O (or max 8 cmH2O with hypoxia)'*<br>*Warning shown if field's value is smaller than 5: 'LUS exam should be performed with a PEEP level of 5 cmH2O (or max 8 cmH2O with hypoxia)'*<br>*Field type*: Numeric field<br>*Variable name*: PEEP_LUS_2<br>*Field required*: Required<br>*Field min*: 3<br>*Field max*: 15<br>*Measurement Unit*: cmH2O	[ ] cmH2O
	RIGHT LUNG	
14.3.5	***If 'Calculation FU LUS' is equal to '0' answer this question:***<br>Anterior region 1<br>*Field type*: Radiobutton<br>*Variable name*: RA1_2<br>*Field required*: Required<br>*Option group name*: LUS exam score	◯ A-pattern<br>◯ B-pattern <50%<br>◯ B-pattern >50%<br>◯ Consolidation
14.3.6	***If 'Calculation FU LUS' is equal to '0' answer this question:***<br>Anterior region 2<br>*Field type*: Radiobutton<br>*Variable name*: RA2_2<br>*Field required*: Required<br>*Option group name*: LUS exam score	◯ A-pattern<br>◯ B-pattern <50%<br>◯ B-pattern >50%<br>◯ Consolidation
14.3.7	***If 'Calculation FU LUS' is equal to '0' answer this question:***<br>Lateral region 3<br>*Field type*: Radiobutton<br>*Variable name*: RL3_2<br>*Field required*: Required<br>*Option group name*: LUS exam score	◯ A-pattern<br>◯ B-pattern <50%<br>◯ B-pattern >50%<br>◯ Consolidation

14.3.8	***If 'Calculation FU LUS' is equal to '0' answer this question:***<br>Lateral region 4<br>*Field type*: Radiobutton<br>*Variable name*: RL4_2<br>*Field required*: Required<br>*Option group name*: LUS exam score	◯ A-pattern<br>◯ B-pattern <50%<br>◯ B-pattern >50%<br>◯ Consolidation
14.3.9	***If 'Calculation FU LUS' is equal to '0' answer this question:***<br>Posterior region 5<br>*Field type*: Radiobutton<br>*Variable name*: RP5_2<br>*Field required*: Required<br>*Option group name*: LUS exam score	◯ A-pattern<br>◯ B-pattern <50%<br>◯ B-pattern >50%<br>◯ Consolidation
14.3.10	***If 'Calculation FU LUS' is equal to '0' answer this question:***<br>Posterior region 6<br>*Field type*: Radiobutton<br>*Variable name*: RP6_2<br>*Field required*: Required<br>*Option group name*: LUS exam posterior	◯ A-pattern or B-pattern < 50%<br>◯ B-pattern > 50%<br>◯ Consolidation
	LEFT LUNG	
14.3.12	***If 'Calculation FU LUS' is equal to '0' answer this question:***<br>Anterior region 1<br>*Field type*: Radiobutton<br>*Variable name*: LA1_2<br>*Field required*: Required<br>*Option group name*: LUS exam score	◯ A-pattern<br>◯ B-pattern <50%<br>◯ B-pattern >50%<br>◯ Consolidation
14.3.13	***If 'Calculation FU LUS' is equal to '0' answer this question:***<br>Anterior region 2<br>*Field type*: Radiobutton<br>*Variable name*: LA2_2<br>*Field required*: Required<br>*Option group name*: LUS exam score	◯ A-pattern<br>◯ B-pattern <50%<br>◯ B-pattern >50%<br>◯ Consolidation
14.3.14	***If 'Calculation FU LUS' is equal to '0' answer this question:***<br>Lateral region 3<br>*Field type*: Radiobutton<br>*Variable name*: LL3_2<br>*Field required*: Required<br>*Option group name*: LUS exam score	◯ A-pattern<br>◯ B-pattern <50%<br>◯ B-pattern >50%<br>◯ Consolidation

14.3.15

If 'Calculation FU LUS' is equal to '0' answer this question:
Lateral region 4
Field type: Radiobutton
Variable name: LL4_2
Field required: Required
Option group name: LUS exam score

- ○ A-pattern
- ○ B-pattern <50%
- ○ B-pattern >50%
- ○ Consolidation

14.3.16

If 'Calculation FU LUS' is equal to '0' answer this question:
Posterior region 5
Field type: Radiobutton
Variable name: LP5_2
Field required: Required
Option group name: LUS exam score

- ○ A-pattern
- ○ B-pattern <50%
- ○ B-pattern >50%
- ○ Consolidation

14.3.17

If 'Calculation FU LUS' is equal to '0' answer this question:
Posterior region 6
Field type: Radiobutton
Variable name: LP6_2
Field required: Required
Option group name: LUS exam posterior

- ○ A-pattern or B-pattern < 50%
- ○ B-pattern > 50%
- ○ Consolidation

If there are LUS exam regions missing, use the flowchart below to define the ARDS fenotype

---

14.3.19

If 'Calculation FU LUS' is equal to '0' answer this question:
Flowchart Ultrasound
Missing LUS images are complemented by the mean LUS aeration score of the other available LUS images in the concerning region (anterior, lateral, or posterior region).

Field type: Image
Variable name: Flow_US_2
Field required: Not required

14.3.20

If 'Calculation FU LUS' is equal to '0' answer this question:
Result LUS exam
Field type: Calculation
Variable name: Result_LUS_2
Field required: Not required

Template: if ({cal_anterior_2} >= 2) { 'Non-Focal' } else if ({cal_lateral_2} > {cal_posterior_2}){ 'Non-Focal' } else { 'Focal' }

14.3.21

If 'Calculation FU LUS' is equal to '0' answer this question:
Confirm result LUS exam
Field type: Radiobutton
Variable name: Confirm_LUS_2
Field required: Required
Option group name: LUS exam

- ○ Focal
- ○ Non-focal

14.3.22	***If 'Calculation FU LUS' is equal to '0' answer this question:***<br>Calculation anterior<br>*Field type*: Calculation<br>*Variable name*: cal_anterior_2<br>*Field required*: Not required	*Template:* {RA1_2} + {RA2_2} + {LA1_2} + {LA2_2}
14.3.23	***If 'Calculation FU LUS' is equal to '0' answer this question:***<br>Calculation lateral<br>*Field type*: Calculation<br>*Variable name*: cal_lateral_2<br>*Field required*: Not required	*Template:* {RL3_2}+{RL4_2}+{LL3_2}+{LL4_2}
14.3.24	***If 'Calculation FU LUS' is equal to '0' answer this question:***<br>Calculation posterior<br>*Field type*: Calculation<br>*Variable name*: cal_posterior_2<br>*Field required*: Not required	*Template:* {RP5_2}+{RP6_2}+{LP5_2}+{LP6_2}
	**SOFA SCORE**	
14.2.1.6	***If 'Location of the patient' is equal to 'Intensive Care Unit' answer this question:***<br>PaO2/FiO2 (mmHg (kPa))<br>Worst value of this day. If there is no value for this day please enter missing data.<br>*Field type*: Radiobutton<br>*Variable name*: SOFA_Resp_6<br>*Field required*: Required<br>*Option group name*: Respiration	◯ ≥ 400 (53.3)<br>◯ < 400 (53.3)<br>◯ < 300 (40)<br>◯ < 200 (26.7) with respiratory support<br>◯ < 100 (13.3) with respiratory support
14.2.1.7	***If 'Location of the patient' is equal to 'Intensive Care Unit' answer this question:***<br>Platelets (x10^3/µL)<br>Worst value of this day. If there is no value for this day please enter missing data.<br>*Field type*: Radiobutton<br>*Variable name*: SOFA_Coag_6<br>*Field required*: Required<br>*Option group name*: Coagulation	◯ ≥ 150<br>◯ < 150<br>◯ < 100<br>◯ < 50<br>◯ < 20
14.2.1.8	***If 'Location of the patient' is equal to 'Intensive Care Unit' answer this question:***<br>Bilirubin (µmol/L(mg/dL))<br>Worst value of this day. If there is no value for this day please enter missing data.<br>*Field type*: Radiobutton<br>*Variable name*: SOFA_Live_6<br>*Field required*: Required<br>*Option group name*: Liver	◯ < 20 (< 1.2)<br>◯ 20-32 (1.2-1.9)<br>◯ 33-101 (2.0-5.9)<br>◯ 102-204 (6.0-11.9)<br>◯ > 204 (> 12.0)

14.2.1.9	***If 'Location of the patient' is equal to 'Intensive Care Unit' answer this question:***<br>Cardiovascular<br>Worst value of this day. If there is no value for this day please enter missing data.<br><br>*Field type*: Radiobutton<br>*Variable name*: SOFA_Card_6<br>*Field required*: Required<br>*Option group name*: Cardiovascular	◯ MAP ≥ 70 mm/Hg without dobutamine, epinephrine or norepinephrine<br>◯ MAP < 70 mm/Hg without dobutamine, epinephrine or norepinephrin<br>◯ Dopamine < 5 μg/kg/min or dobutamine (any dose)<br>◯ Dopamine > 5 μg/kg/min OR epinephrine ≤ 0.1 μg/kg/min OR norepinephrine ≤ 0.1 μg/kg/min<br>◯ Dopamine > 15 μg/kg/min OR epinephrine > 0.1 μg/kg/min OR norepinephrine > 0.1 μg/kg/min
14.2.1.10	***If 'Location of the patient' is equal to 'Intensive Care Unit' answer this question:***<br>Glasgow coma scale<br>Only fill in the score when the patient is not sedated. Choose the worst value of this day. If there is no value for this day or the patient is sedated, please enter missing data.<br><br>*Field type*: Radiobutton<br>*Variable name*: SOFA_Nerv_6<br>*Field required*: Required<br>*Option group name*: Nervous	◯ 15<br>◯ 13-14<br>◯ 10-12<br>◯ 6-9<br>◯ < 6
14.2.1.11	***If 'Location of the patient' is equal to 'Intensive Care Unit' answer this question:***<br>Creatinine [or urine output]<br>Worst value of this day. If there is no value for this day please enter missing data.<br><br>*Field type*: Radiobutton<br>*Variable name*: SOFA_Kidn_6<br>*Field required*: Required<br>*Option group name*: Kidneys	◯ < 110 (< 1.2)<br>◯ 110-170 (1.2-1.9)<br>◯ 171-299 (2.0-3.4)<br>◯ 300-440 (3.5-4.9) [or < 500]<br>◯ > 440 (> 5.0) [or < 200]
14.2.1.12	***If 'Location of the patient' is equal to 'Intensive Care Unit' answer this question:***<br>SOFA score<br>*Field type*: Calculation<br>*Variable name*: SOFA_scor_6<br>*Field required*: Not required	*Template:* {SOFA_Resp_6}+{SOFA_Coag_6}+{SOFA_Live_6}+{SOFA_Card_6}+{SOFA_Nerv_6}+{SOFA_Kidn_6}
	**EVENTS**	

14.2.1.14	***If 'Location of the patient' is equal to 'Intensive Care Unit' answer this question:***<br>Did an event occur?<br>• Renal Replacement Therapy during any time of the day.<br>• Placement of a tracheostomy during any time of the day<br>• Use of inhaled vasodilators during any time of the day<br>• Use of airway pressure release ventlation during any time of the day<br>• Use of ECMO during any time of the day<br><br>*Field type*: Checkbox<br>*Variable name*: Event_6<br>*Field required*: Required<br>*Option group name*: Events	☐ None<br>☐ Use of renal replacement therapy<br>☐ Placement of a tracheostomy<br>☐ Use of inhaled vasodilators<br>☐ Use of airway pressure release ventilation<br>☐ Use of ECMO<br>☐ Continuous infusion of neuromuscular blocking agents
14.2.1.15	***If 'Location of the patient' is equal to 'Intensive Care Unit' answer this question:***<br>Calculation for incorrect answer event<br>*Field type*: Calculation<br>*Variable name*: Event_calc_incorrect_6<br>*Field required*: Not required	*Template:* '##allowempty##' var splitted = "{Event_6}".split(';'); if (splitted.indexOf("0") > -1 && (splitted.indexOf("1") > -1 || splitted.indexOf("2") > -1 || splitted.indexOf("3") > -1 || splitted.indexOf("4") > -1 || splitted.indexOf("5") > -1 || splitted.indexOf("6") > -1)) { '1'; } else { '0'; };
	Combination **NOT** possible! **NONE** cannot be chosen when other options are selected!	
	**COMPLICATIONS**	
14.2.1.17	***If 'Location of the patient' is equal to 'Intensive Care Unit' answer this question:***<br>Did a complication occur?<br>Only tick the checkbox at the day of diagnosis.<br>• Pneumothorax: Air in the pleural cavity developed after randomization confirmed by a radiologist on a CT-thorax or chest radiograph for which a drain has been placed.<br>• Ventilator Associated Pneumonia: Clinical Pulmonary Infection Score (CPIS) > 5 with an infiltration on CXR and developed after intubation.<br><br>*Field type*: Checkbox<br>*Variable name*: Compl_6<br>*Field required*: Required<br>*Option group name*: Complications	☐ None<br>☐ Diagnosis of pneumothorax<br>☐ Diagnosis of Ventilator Associated Pneumonia
14.2.1.18	***If 'Location of the patient' is equal to 'Intensive Care Unit' answer this question:***<br>Calculation for incorrect answer complications<br>*Field type*: Calculation<br>*Variable name*: Compl_calc_incorrect_6<br>*Field required*: Not required	*Template:* '##allowempty##' var splitted = "{Compl_6}".split(';'); if (splitted.indexOf("0") > -1 && (splitted.indexOf("1") > -1 || splitted.indexOf("2") > -1 || splitted.indexOf("3") > -1 || splitted.indexOf("4") > -1 || splitted.indexOf("5") > -1 || splitted.indexOf("6") > -1)) { '1'; } else { '0'; };
	Combination **NOT** possible! **NONE** cannot be chosen when other options are selected!	

	**VENTILATION DATA AT 6:00 AM**	
14.2.1.2.2	***If 'Type of respiratory support' is equal to 'Mechanical ventilation' answer this question:***<br>RASS score<br>+4 Combative +3 Very agitated +2 Agitated +1 Restless 0 Alert and calm -1 Drowsy -2 Light sedation -3 Moderate sedation -4 Deep sedation -5 Unarousable<br>*Field type*: Radiobutton<br>*Variable name*: Rass_6<br>*Field required*: Required<br>*Option group name*: RASS Score	○ ≥ 0<br>○ - 1<br>○ - 2<br>○ - 3<br>○ - 4<br>○ - 5
14.2.1.2.3	***If 'Type of respiratory support' is equal to 'Mechanical ventilation' answer this question:***<br>Mode of ventilation<br>*Field type*: Radiobutton<br>*Variable name*: Mode_ven_6<br>*Field required*: Required<br>*Option group name*: Mode ventilation	○ Controlled<br>○ Spontaneous<br>○ Adaptive
14.2.1.2.3.1	***If 'Mode of ventilation' is equal to 'Adaptive' answer this question:***<br>Triggered or timed adaptive ventilation<br>*Field type*: Radiobutton<br>*Variable name*: Trig_timed_6<br>*Field required*: Required<br>*Option group name*: Triggered/Timed	○ Triggered<br>○ Timed
14.2.1.2.4	***If 'Type of respiratory support' is equal to 'Mechanical ventilation' answer this question:***<br>Tidal volume<br>*Warning shown if field's value is smaller than 100: 'Low value! Please control data input or make a comment.'*<br>*Warning shown if field's value is larger than 900: 'High value! Please control data input or make a comment.'*<br>*Field type*: Numeric field<br>*Variable name*: Tidal_6<br>*Field required*: Required<br>*Field min*: 50<br>*Field max*: 1200<br>*Measurement Unit*: ml	[ ] ml

14.2.1.2.5 ***If 'Type of respiratory support' is equal to 'Mechanical ventilation' answer this question:***
Total respiratory rate
Warning shown if field's value is smaller than 10: 'Low value! Please control data input or make a comment.'
Warning shown if field's value is larger than 35: 'High value! Please control data input or make a comment.'
Field type: Numeric field
Variable name: TRR_6
Field required: Required
Field min: 4
Field max: 50
Measurement Unit: breaths per minut

[ ] breaths per minut

14.2.1.2.6 ***If 'Type of respiratory support' is equal to 'Mechanical ventilation' answer this question:***
PEEP
Warning shown if field's value is smaller than 5: 'Low value! Please control data input or make a comment.'
Warning shown if field's value is larger than 19: 'High value! Please control data input or make a comment.'
Field type: Numeric field
Variable name: Peep_6
Field required: Required
Field min: 0
Field max: 25
Measurement Unit: cmH2O

[ ] cmH2O

14.2.1.2.3.2 ***If 'Mode of ventilation' is not equal to 'Spontaneous' answer this question:***
Pplateau
Warning shown if field's value is smaller than or equal to 7: 'Low value! Please control data input or make a comment.'
Warning shown if field's value is larger than or equal to 30: 'High value! Please control data input or make a comment.'
Field type: Numeric field
Variable name: Pplat_6
Field required: Required
Field min: 5
Field max: 40
Measurement Unit: cmH2O

[ ] cmH2O

14.2.1.2.3.3 ***If 'Mode of ventilation' is not equal to 'Spontaneous' answer this question:***
Pmax/ Ppeak
Warning shown if field's value is smaller than 10: 'Low value! Please control data input or make a comment.'
Warning shown if field's value is larger than 29: 'High value! Please control data input or make a comment.'
Field type: Numeric field
Variable name: Pmax_6
Field required: Required
Field min: 5
Field max: 50
Measurement Unit: cmH2O

[ ] cmH2O

14.2.1.2.3.4	***If 'Mode of ventilation' is equal to 'Spontaneous' answer this question:***<br>Pressure support<br>*Warning shown if field's value is smaller than 5: 'Low value! Please control data input or make a comment.'*<br>*Warning shown if field's value is larger than 19: 'High value! Please control data input or make a comment.'*<br>*Field type*: Numeric field<br>*Variable name*: Psup_6<br>*Field required*: Required<br>*Field min*: 0<br>*Field max*: 30<br>*Measurement Unit*: cmH2O	[ ] cmH2O
14.2.1.2.7	***If 'Type of respiratory support' is equal to 'Mechanical ventilation' answer this question:***<br>Unit used for EtCO2, PaO2 and PaCO2<br>*Field type*: Radiobutton<br>*Variable name*: mmhg_kpa_6<br>*Field required*: Required<br>*Option group name*: kPa/mmHg	○ kPa<br>○ mmHg
14.2.1.2.7.1	***If 'Unit used for EtCO2, PaO2 and PaCO2' is equal to 'kPa' answer this question:***<br>EtCO2<br>*Warning shown if field's value is smaller than 2: 'Low value! Please control data input or make a comment.'*<br>*Warning shown if field's value is larger than 8: 'High value! Please control data input or make a comment.'*<br>*Field type*: Numeric field<br>*Variable name*: EtCO2_kpa_6<br>*Field required*: Required<br>*Field min*: 1<br>*Field max*: 20<br>*Measurement Unit*: kPa	[ ] kPa
14.2.1.2.7.2	***If 'Unit used for EtCO2, PaO2 and PaCO2' is equal to 'mmHg' answer this question:***<br>EtCO2<br>*Warning shown if field's value is smaller than 15: 'Low value! Please control data input or make a comment.'*<br>*Warning shown if field's value is larger than 59: 'High value! Please control data input or make a comment.'*<br>*Field type*: Numeric field<br>*Variable name*: EtCO2_mmhg_6<br>*Field required*: Required<br>*Field min*: 7<br>*Field max*: 140<br>*Measurement Unit*: mmHg	[ ] mmHg

14.2.1.2.8	***If 'Type of respiratory support' is equal to 'Mechanical ventilation' answer this question:***<br>FiO2 (closest to AGB, in decimal, e.g. 0.35)<br>*Field type*: Numeric field<br>*Variable name*: FiO2_6<br>*Field required*: Required<br>*Field min*: 0.20999999999999999222284388<br>*Field max*: 1	
14.2.1.2.9	***If 'Type of respiratory support' is equal to 'Mechanical ventilation' answer this question:***<br>SpO2 (closest to AGB)<br>*Notice shown if field's value is smaller than 80: 'Low value! Please control data input or make a comment.'*<br>*Field type*: Numeric field<br>*Variable name*: SpO2_6<br>*Field required*: Required<br>*Field min*: 60<br>*Field max*: 100<br>*Measurement Unit*: %	%
14.2.1.2.10	***If 'Type of respiratory support' is equal to 'Mechanical ventilation' answer this question:***<br>Recruitment maneuvre on this day<br>*Field type*: Radiobutton<br>*Variable name*: Recruit_6<br>*Field required*: Required<br>*Option group name*: Yes/No	◯ Yes<br>◯ No
14.2.1.2.10.1	***If 'Recruitment maneuvre on this day' is equal to 'Yes' answer this question:***<br>Amount of recruitment maneuvres<br>*Warning shown if field's value is larger than 9: 'High value! Please control data input or make a comment'*<br>Times on this day from 00:00 until 23:59.<br>*Field type*: Numeric field<br>*Variable name*: Recruit_times_6<br>*Field required*: Required<br>*Field min*: 0<br>*Field max*: 20<br>*Measurement Unit*: times	times
14.2.1.2.11	***If 'Type of respiratory support' is equal to 'Mechanical ventilation' answer this question:***<br>Prone positioning on this day<br>*Field type*: Radiobutton<br>*Variable name*: Prone_6<br>*Field required*: Required<br>*Option group name*: Yes/No	◯ Yes<br>◯ No

14.2.1.2.11.1 ***If 'Prone positioning on this day' is equal to 'Yes' answer this question:***
Duration of prone posistioning
Hours on this day from 00:00 until 23:59.

Field type: Numeric field
Variable name: Prone_hours_6
Field required: Required
Field min: 0
Field max: 24
Measurement Unit: hours

[ ] hours

14.2.1.2.12 ***If 'Type of respiratory support' is equal to 'Mechanical ventilation' answer this question:***
Do you expect extubation within 48 hours based on the respiration parameters?
If you expect extubation within 48 hours based on the respiration parameters, the ventilation strategy can be abandoned. Please look in our mechanical ventilation handbook for guidelines. When the patient's condition worsens, restart the ventilation strategy according to the protocol.

Field type: Radiobutton
Variable name: ext_6
Field required: Required
Option group name: Yes/No

○ Yes
○ No

ARTERIAL BLOOD GAS AT 6:00

14.2.1.2.14 ***If 'Type of respiratory support' is equal to 'Mechanical ventilation' answer this question:***
Arterial pH
Warning shown if field's value is smaller than 7.0: 'Low value! Please control data input or make a comment.'
Warning shown if field's value is larger than 7.7: 'High value! Please control data input or make a comment.'
Field type: Numeric field
Variable name: ArtpH_6
Field required: Required
Field min: 6.5
Field max: 8
Measurement Unit: pH

[ ] pH

14.2.1.2.15 ***If 'Type of respiratory support' is equal to 'Mechanical ventilation' answer this question:***
Arterial bicarbonate
Warning shown if field's value is smaller than 10: 'Low value! Please control data input or make a comment.'
Warning shown if field's value is larger than 39: 'High value! Please control data input or make a comment.'
Field type: Numeric field
Variable name: Bic_6
Field required: Required
Field min: 2
Field max: 70
Measurement Unit: mmol/l

[ ] mmol/l

14.2.1.2.16	***If 'Type of respiratory support' is equal to 'Mechanical ventilation' answer this question:***<br>Arterial lactate<br>*Warning shown if field's value is larger than 9.9: 'High value! Please control data input or make a comment.'*<br>*Field type*: Numeric field<br>*Variable name*: Lac_6<br>*Field required*: Required<br>*Field min*: 0<br>*Field max*: 25<br>*Measurement Unit*: mmol/L	mmol/L
14.2.1.2.17	***If 'Type of respiratory support' is equal to 'Mechanical ventilation' answer this question:***<br>Arterial saturation<br>*Notice shown if field's value is smaller than 80: 'Low value! Please control data input or make a comment.'*<br>*Field type*: Numeric field<br>*Variable name*: Sat_6<br>*Field required*: Required<br>*Field min*: 60<br>*Field max*: 100<br>*Measurement Unit*: %	%
14.2.1.2.7.3	***If 'Unit used for EtCO2, PaO2 and PaCO2' is equal to 'kPa' answer this question:***<br>Arterial PaO2<br>*Warning shown if field's value is smaller than 7: 'Low value! Please control data input or make a comment.'*<br>*Warning shown if field's value is larger than 18: 'High value! Please control data input or make a comment.'*<br>*Field type*: Numeric field<br>*Variable name*: PaO2_kpa_6<br>*Field required*: Required<br>*Field min*: 4<br>*Field max*: 60<br>*Measurement Unit*: kPa	kPa
14.2.1.2.7.4	***If 'Unit used for EtCO2, PaO2 and PaCO2' is equal to 'mmHg' answer this question:***<br>Arterial PaO2<br>*Warning shown if field's value is smaller than 50: 'Low value! Please control data input or make a comment.'*<br>*Warning shown if field's value is larger than 150: 'High value! Please control data input or make a comment.'*<br>*Field type*: Numeric field<br>*Variable name*: PaO2_mmhg_6<br>*Field required*: Required<br>*Field min*: 28<br>*Field max*: 500<br>*Measurement Unit*: mmHg	mmHg

Number	Question	Answers
14.2.1.2.7.5	***If 'Unit used for EtCO2, PaO2 and PaCO2' is equal to 'kPa' answer this question:***<br>Arterial PaCO2<br>*Warning shown if field's value is smaller than 2: 'Low value! Please control data input or make a comment.'*<br>*Warning shown if field's value is larger than 8: 'High value! Please control data input or make a comment.'*<br>*Field type*: Numeric field<br>*Variable name*: PaCO2_kpa_6<br>*Field required*: Required<br>*Field min*: 1<br>*Field max*: 20<br>*Measurement Unit*: kPa	[ ] kPa
14.2.1.2.7.6	***If 'Unit used for EtCO2, PaO2 and PaCO2' is equal to 'mmHg' answer this question:***<br>Arterial PaCO2<br>*Warning shown if field's value is smaller than 15: 'Low value! Please control data input or make a comment.'*<br>*Warning shown if field's value is larger than 59: 'High value! Please control data input or make a comment.'*<br>*Field type*: Numeric field<br>*Variable name*: PaCO2_mmhg_6<br>*Field required*: Required<br>*Field min*: 7<br>*Field max*: 140<br>*Measurement Unit*: mmHg	[ ] mmHg

15. Daily data - Day 7

Number	Question	Answers
15.1	Date of study day<br>*Field type*: Calculation<br>*Variable name*: date_7<br>*Field required*: Not required	*Template:* var randomization= moment('{castorRandomizedDateAndTime}', 'DD-MM-YYYY'); var newDate = randomization.add(7, 'days'); newDate.format('DD-MM-YYYY');
15.2	***If 'Is the patient hospitalized during any time of the day?' is equal to 'Yes' answer this question:***<br>Is the patient hospitalized during any time of the day?<br>*Notice shown if field's value is equal to No: 'DAILY DATA COMPLETE, GO TO FOLLOW UP.'*<br>*Field type*: Radiobutton<br>*Variable name*: Hospital_patient_7<br>*Field required*: Required<br>*Option group name*: Yes/No	○ Yes<br>○ No

15.2.1	***If 'Is the patient hospitalized during any time of the day?' is equal to 'Yes' answer this question:***<br>Location of the patient<br>At 6:00 AM<br><br>*Field type*: Radiobutton<br>*Variable name*: Loc_ICUhosp_7<br>*Field required*: Required<br>*Option group name*: ICU/hospital	◯ Intensive Care Unit<br>◯ Hospital ward
15.2.1.1	***If 'Location of the patient' is equal to 'Hospital ward' answer this question:***<br>Type of respiratory support<br>At 6:00 AM<br><br>*Field type*: Radiobutton<br>*Variable name*: Resp_hospsupp_7<br>*Field required*: Required<br>*Option group name*: resp_supp_hosp	◯ No oxygen therapy<br>◯ Oxygen by mask or nasal prongs<br>◯ High Flow Nasal Oxygen (HFNO) therapy<br>◯ Non-invasive ventilation (NIV)
15.2.1.2	***If 'Location of the patient' is equal to 'Intensive Care Unit' answer this question:***<br>Type of respiratory support<br>At 6:00 AM<br><br>*Field type*: Radiobutton<br>*Variable name*: Resp_supp_7<br>*Field required*: Required<br>*Option group name*: Respiratory support	◯ No oxygen therapy<br>◯ Oxygen by maks or nasal prongs<br>◯ High Flow Nasal Oxygen (HFNO) therapy<br>◯ Non-invasive ventilation (NIV)<br>◯ Mechanical ventilation
15.2.1.3	***If 'Location of the patient' is equal to 'Intensive Care Unit' answer this question:***<br>Daily cumulative fluid balance<br>*Field type*: Radiobutton<br>*Variable name*: Cum_fluid_7<br>*Field required*: Required<br>*Option group name*: Negative/positve	◯ Negative<br>◯ Positive
15.2.1.4	***If 'Location of the patient' is equal to 'Intensive Care Unit' answer this question:***<br>Daily cumulative fluid balance<br>*Warning shown if field's value is larger than or equal to 4000: 'High value! Please control data input or make a comment.'*<br>From 0:00 until 0:00<br><br>*Field type*: Numeric field<br>*Variable name*: Cum_fluid_ml_7<br>*Field required*: Required<br>*Field min*: 0<br>*Field max*: 30000<br>*Measurement Unit*: ml	[ ] ml
	**SOFA SCORE**	

15.2.1.6	***If 'Location of the patient' is equal to 'Intensive Care Unit' answer this question:***<br>PaO2/FiO2 (mmHg (kPa))<br>Worst value of this day. If there is no value for this day please enter missing data.<br><br>*Field type*: Radiobutton<br>*Variable name*: SOFA_Resp_7<br>*Field required*: Required<br>*Option group name*: Respiration	◯ ≥ 400 (53.3)<br>◯ < 400 (53.3)<br>◯ < 300 (40)<br>◯ < 200 (26.7) with respiratory support<br>◯ < 100 (13.3) with respiratory support
15.2.1.7	***If 'Location of the patient' is equal to 'Intensive Care Unit' answer this question:***<br>Platelets (x10^3/µL)<br>Worst value of this day. If there is no value for this day please enter missing data.<br><br>*Field type*: Radiobutton<br>*Variable name*: SOFA_Coag_7<br>*Field required*: Required<br>*Option group name*: Coagulation	◯ ≥ 150<br>◯ < 150<br>◯ < 100<br>◯ < 50<br>◯ < 20
15.2.1.8	***If 'Location of the patient' is equal to 'Intensive Care Unit' answer this question:***<br>Bilirubin (µmol/L(mg/dL))<br>Worst value of this day. If there is no value for this day please enter missing data.<br><br>*Field type*: Radiobutton<br>*Variable name*: SOFA_Live_7<br>*Field required*: Required<br>*Option group name*: Liver	◯ < 20 (< 1.2)<br>◯ 20-32 (1.2-1.9)<br>◯ 33-101 (2.0-5.9)<br>◯ 102-204 (6.0-11.9)<br>◯ > 204 (> 12.0)
15.2.1.9	***If 'Location of the patient' is equal to 'Intensive Care Unit' answer this question:***<br>Cardiovascular<br>Worst value of this day. If there is no value for this day please enter missing data.<br><br>*Field type*: Radiobutton<br>*Variable name*: SOFA_Card_7<br>*Field required*: Required<br>*Option group name*: Cardiovascular	◯ MAP ≥ 70 mm/Hg without dobutamine, epinephrine or norepinephrine<br>◯ MAP < 70 mm/Hg without dobutamine, epinephrine or norepinephrin<br>◯ Dopamine < 5 µg/kg/min or dobutamine (any dose)<br>◯ Dopamine > 5 µg/kg/min OR epinephrine ≤ 0.1 µg/kg/min OR norepinephrine ≤ 0.1 µg/kg/min<br>◯ Dopamine > 15 µg/kg/min OR epinephrine > 0.1 µg/kg/min OR norepinephrine > 0.1 µg/kg/min
15.2.1.10	***If 'Location of the patient' is equal to 'Intensive Care Unit' answer this question:***<br>Glasgow coma scale<br>Only fill in the score when the patient is not sedated. Choose the worst value of this day. If there is no value for this day or the patient is sedated, please enter missing data.<br><br>*Field type*: Radiobutton<br>*Variable name*: SOFA_Nerv_7<br>*Field required*: Required<br>*Option group name*: Nervous	◯ 15<br>◯ 13-14<br>◯ 10-12<br>◯ 6-9<br>◯ < 6

15.2.1.11	***If 'Location of the patient' is equal to 'Intensive Care Unit' answer this question:***<br>Creatinine [or urine output]<br>Worst value of this day. If there is no value for this day please enter missing data.<br>*Field type*: Radiobutton<br>*Variable name*: SOFA_Kidn_7<br>*Field required*: Required<br>*Option group name*: Kidneys	◯ < 110 (< 1.2)<br>◯ 110-170 (1.2-1.9)<br>◯ 171-299 (2.0-3.4)<br>◯ 300-440 (3.5-4.9) [or < 500]<br>◯ > 440 (> 5.0) [or < 200]
15.2.1.12	***If 'Location of the patient' is equal to 'Intensive Care Unit' answer this question:***<br>SOFA score<br>*Field type*: Calculation<br>*Variable name*: SOFA_scor_7<br>*Field required*: Not required	*Template:* {SOFA_Resp_7}+{SOFA_Coag_7}+{SOFA_Live_7}+{SOFA_Card_7}+{SOFA_Nerv_7}+{SOFA_Kidn_7}
	**EVENTS**	
15.2.1.14	***If 'Location of the patient' is equal to 'Intensive Care Unit' answer this question:***<br>Did an event occur?<br>• Renal Replacement Therapy during any time of the day.<br>• Placement of a tracheostomy during any time of the day<br>• Use of inhaled vasodilators during any time of the day<br>• Use of airway pressure release ventlation during any time of the day<br>• Use of ECMO during any time of the day<br>*Field type*: Checkbox<br>*Variable name*: Event_7<br>*Field required*: Required<br>*Option group name*: Events	☐ None<br>☐ Use of renal replacement therapy<br>☐ Placement of a tracheostomy<br>☐ Use of inhaled vasodilators<br>☐ Use of airway pressure release ventilation<br>☐ Use of ECMO<br>☐ Continuous infusion of neuromuscular blocking agents
15.2.1.15	***If 'Location of the patient' is equal to 'Intensive Care Unit' answer this question:***<br>Calculation for incorrect answer event<br>*Field type*: Calculation<br>*Variable name*: Event_calc_incorrect_7<br>*Field required*: Not required	*Template:* '##allowempty##' var splitted = "{Event_7}".split(';'); if (splitted.indexOf("0") > -1 && (splitted.indexOf("1") > -1 || splitted.indexOf("2") > -1 || splitted.indexOf("3") > -1 || splitted.indexOf("4") > -1 || splitted.indexOf("5") > -1 || splitted.indexOf("6") > -1)) { '1'; } else { '0'; };
	Combination **NOT** possible! **NONE** cannot be chosen when other options are selected!	
	**COMPLICATIONS**	

15.2.1.17	***If 'Location of the patient' is equal to 'Intensive Care Unit' answer this question:***<br>Did a complication occur?<br>Only tick the checkbox at the day of diagnosis.<br>• Pneumothorax: Air in the pleural cavity developed after randomization confirmed by a radiologist on a CT-thorax or chest radiograph for which a drain has been placed.<br>• Ventilator Associated Pneumonia: Clinical Pulmonary Infection Score (CPIS) > 5 with an infiltration on CXR and developed after intubation.<br>*Field type*: Checkbox<br>*Variable name*: Compl_7<br>*Field required*: Required<br>*Option group name*: Complications	☐ None<br>☐ Diagnosis of pneumothorax<br>☐ Diagnosis of Ventilator Associated Pneumonia
15.2.1.18	***If 'Location of the patient' is equal to 'Intensive Care Unit' answer this question:***<br>Calculation for incorrect answer complications<br>*Field type*: Calculation<br>*Variable name*: Compl_calc_incorrect_7<br>*Field required*: Not required	*Template:* '##allowempty##' var splitted = "{Compl_7}".split(';'); if (splitted.indexOf("0") > -1 && (splitted.indexOf("1") > -1 || splitted.indexOf("2") > -1 || splitted.indexOf("3") > -1 || splitted.indexOf("4") > -1 || splitted.indexOf("5") > -1 || splitted.indexOf("6") > -1)) { '1'; } else { '0'; };
	Combination **NOT** possible! **NONE** cannot be chosen when other options are selected!	
	**VENTILATION DATA AT 6:00 AM**	
15.2.1.2.2	***If 'Type of respiratory support' is equal to 'Mechanical ventilation' answer this question:***<br>RASS score<br>+4 Combative +3 Very agitated +2 Agitated +1 Restless 0 Alert and calm -1 Drowsy -2 Light sedation -3 Moderate sedation -4 Deep sedation -5 Unarousable<br>*Field type*: Radiobutton<br>*Variable name*: Rass_7<br>*Field required*: Required<br>*Option group name*: RASS Score	○ ≥ 0<br>○ - 1<br>○ - 2<br>○ - 3<br>○ - 4<br>○ - 5
15.2.1.2.3	***If 'Type of respiratory support' is equal to 'Mechanical ventilation' answer this question:***<br>Mode of ventilation<br>*Field type*: Radiobutton<br>*Variable name*: Mode_ven_7<br>*Field required*: Required<br>*Option group name*: Mode ventilation	○ Controlled<br>○ Spontaneous<br>○ Adaptive
15.2.1.2.3.1	***If 'Mode of ventilation' is equal to 'Adaptive' answer this question:***<br>Triggered or timed adaptive ventilation<br>*Field type*: Radiobutton<br>*Variable name*: Trig_timed_7<br>*Field required*: Required<br>*Option group name*: Triggered/Timed	○ Triggered<br>○ Timed

15.2.1.2.4	***If 'Type of respiratory support' is equal to 'Mechanical ventilation' answer this question:***<br>Tidal volume<br>*Warning shown if field's value is smaller than 100: 'Low value! Please control data input or make a comment.'*<br>*Warning shown if field's value is larger than 900: 'High value! Please control data input or make a comment.'*<br>*Field type*: Numeric field<br>*Variable name*: Tidal_7<br>*Field required*: Required<br>*Field min*: 50<br>*Field max*: 1200<br>*Measurement Unit*: ml	ml
15.2.1.2.5	***If 'Type of respiratory support' is equal to 'Mechanical ventilation' answer this question:***<br>Total respiratory rate<br>*Warning shown if field's value is smaller than 10: 'Low value! Please control data input or make a comment.'*<br>*Warning shown if field's value is larger than 35: 'High value! Please control data input or make a comment.'*<br>*Field type*: Numeric field<br>*Variable name*: TRR_7<br>*Field required*: Required<br>*Field min*: 4<br>*Field max*: 50<br>*Measurement Unit*: breaths per minut	breaths per minut
15.2.1.2.6	***If 'Type of respiratory support' is equal to 'Mechanical ventilation' answer this question:***<br>PEEP<br>*Warning shown if field's value is smaller than 5: 'Low value! Please control data input or make a comment.'*<br>*Warning shown if field's value is larger than 19: 'High value! Please control data input or make a comment.'*<br>*Field type*: Numeric field<br>*Variable name*: Peep_7<br>*Field required*: Required<br>*Field min*: 0<br>*Field max*: 25<br>*Measurement Unit*: cmH2O	cmH2O
15.2.1.2.3.2	***If 'Mode of ventilation' is not equal to 'Spontaneous' answer this question:***<br>Pplateau<br>*Warning shown if field's value is smaller than or equal to 7: 'Low value! Please control data input or make a comment.'*<br>*Warning shown if field's value is larger than or equal to 30: 'High value! Please control data input or make a comment.'*<br>*Field type*: Numeric field<br>*Variable name*: Pplat_7<br>*Field required*: Required<br>*Field min*: 5<br>*Field max*: 40<br>*Measurement Unit*: cmH2O	cmH2O

15.2.1.2.3.3	***If 'Mode of ventilation' is not equal to 'Spontaneous' answer this question:***<br>Pmax/ Ppeak<br>*Warning shown if field's value is smaller than 10: 'Low value! Please control data input or make a comment.'*<br>*Warning shown if field's value is larger than 29: 'High value! Please control data input or make a comment.'*<br>*Field type*: Numeric field<br>*Variable name*: Pmax_7<br>*Field required*: Required<br>*Field min*: 5<br>*Field max*: 50<br>*Measurement Unit*: cmH2O	[ ] cmH2O
15.2.1.2.3.4	***If 'Mode of ventilation' is equal to 'Spontaneous' answer this question:***<br>Pressure support<br>*Warning shown if field's value is smaller than 5: 'Low value! Please control data input or make a comment.'*<br>*Warning shown if field's value is larger than 19: 'High value! Please control data input or make a comment.'*<br>*Field type*: Numeric field<br>*Variable name*: Psup_7<br>*Field required*: Required<br>*Field min*: 0<br>*Field max*: 30<br>*Measurement Unit*: cmH2O	[ ] cmH2O
15.2.1.2.7	***If 'Type of respiratory support' is equal to 'Mechanical ventilation' answer this question:***<br>Unit used for EtCO2, PaO2 and PaCO2<br>*Field type*: Radiobutton<br>*Variable name*: mmhg_kpa_7<br>*Field required*: Required<br>*Option group name*: kPa/mmHg	○ kPa<br>○ mmHg
15.2.1.2.7.1	***If 'Unit used for EtCO2, PaO2 and PaCO2' is equal to 'kPa' answer this question:***<br>EtCO2<br>*Warning shown if field's value is smaller than 2: 'Low value! Please control data input or make a comment.'*<br>*Warning shown if field's value is larger than 8: 'High value! Please control data input or make a comment.'*<br>*Field type*: Numeric field<br>*Variable name*: EtCO2_kpa_7<br>*Field required*: Required<br>*Field min*: 1<br>*Field max*: 20<br>*Measurement Unit*: kPa	[ ] kPa

15.2.1.2.7.2	***If 'Unit used for EtCO2, PaO2 and PaCO2' is equal to 'mmHg' answer this question:***<br>EtCO2<br>*Warning shown if field's value is smaller than 15: 'Low value! Please control data input or make a comment.'*<br>*Warning shown if field's value is larger than 59: 'High value! Please control data input or make a comment.'*<br>*Field type*: Numeric field<br>*Variable name*: EtCO2_mmhg_7<br>*Field required*: Required<br>*Field min*: 7<br>*Field max*: 140<br>*Measurement Unit*: mmHg	mmHg
15.2.1.2.8	***If 'Type of respiratory support' is equal to 'Mechanical ventilation' answer this question:***<br>FiO2 (closest to AGB, in decimal, e.g. 0.35)<br>*Field type*: Numeric field<br>*Variable name*: FiO2_7<br>*Field required*: Required<br>*Field min*: 0.2099999999999999922284388<br>*Field max*: 1	
15.2.1.2.9	***If 'Type of respiratory support' is equal to 'Mechanical ventilation' answer this question:***<br>SpO2 (closest to AGB)<br>*Notice shown if field's value is smaller than 80: 'Low value! Please control data input or make a comment.'*<br>*Field type*: Numeric field<br>*Variable name*: SpO2_7<br>*Field required*: Required<br>*Field min*: 60<br>*Field max*: 100<br>*Measurement Unit*: %	%
15.2.1.2.10	***If 'Type of respiratory support' is equal to 'Mechanical ventilation' answer this question:***<br>Recruitment maneuvre on this day<br>*Field type*: Radiobutton<br>*Variable name*: Recruit_7<br>*Field required*: Required<br>*Option group name*: Yes/No	○ Yes<br>○ No
15.2.1.2.10.1	***If 'Recruitment maneuvre on this day' is equal to 'Yes' answer this question:***<br>Amount of recruitment maneuvres<br>*Warning shown if field's value is larger than 9: 'High value! Please control data input or make a comment'*<br>Times on this day from 00:00 until 23:59.<br>*Field type*: Numeric field<br>*Variable name*: Recruit_times_7<br>*Field required*: Required<br>*Field min*: 0<br>*Field max*: 20<br>*Measurement Unit*: times	times

15.2.1.2.11	***If 'Type of respiratory support' is equal to 'Mechanical ventilation' answer this question:***<br>Prone positioning on this day<br>*Field type*: Radiobutton<br>*Variable name*: Prone_7<br>*Field required*: Required<br>*Option group name*: Yes/No	◯ Yes<br>◯ No
15.2.1.2.11.1	***If 'Prone positioning on this day' is equal to 'Yes' answer this question:***<br>Duration of prone posistioning<br>Hours on this day from 00:00 until 23:59.<br><br>*Field type*: Numeric field<br>*Variable name*: Prone_hours_7<br>*Field required*: Required<br>*Field min*: 0<br>*Field max*: 24<br>*Measurement Unit*: hours	[ ] hours
15.2.1.2.12	***If 'Type of respiratory support' is equal to 'Mechanical ventilation' answer this question:***<br>Do you expect extubation within 48 hours based on the respiration parameters?<br>If you expect extubation within 48 hours based on the respiration parameters, the ventilation strategy can be abandoned. Please look in our mechanical ventilation handbook for guidelines. When the patient's condition worsens, restart the ventilation strategy according to the protocol.<br><br>*Field type*: Radiobutton<br>*Variable name*: ext_7<br>*Field required*: Required<br>*Option group name*: Yes/No	◯ Yes<br>◯ No
	**ARTERIAL BLOOD GAS AT 6:00**	
15.2.1.2.14	***If 'Type of respiratory support' is equal to 'Mechanical ventilation' answer this question:***<br>Arterial pH<br>*Warning shown if field's value is smaller than 7.0: 'Low value! Please control data input or make a comment.'*<br>*Warning shown if field's value is larger than 7.7: 'High value! Please control data input or make a comment.'*<br>*Field type*: Numeric field<br>*Variable name*: ArtpH_7<br>*Field required*: Required<br>*Field min*: 6.5<br>*Field max*: 8<br>*Measurement Unit*: pH	[ ] pH

15.2.1.2.15	***If 'Type of respiratory support' is equal to 'Mechanical ventilation' answer this question:***<br>Arterial bicarbonate<br>*Warning shown if field's value is smaller than 10: 'Low value! Please control data input or make a comment.'*<br>*Warning shown if field's value is larger than 39: 'High value! Please control data input or make a comment.'*<br>*Field type*: Numeric field<br>*Variable name*: Bic_7<br>*Field required*: Required<br>*Field min*: 2<br>*Field max*: 70<br>*Measurement Unit*: mmol/l	mmol/l
15.2.1.2.16	***If 'Type of respiratory support' is equal to 'Mechanical ventilation' answer this question:***<br>Arterial lactate<br>*Warning shown if field's value is larger than 9.9: 'High value! Please control data input or make a comment.'*<br>*Field type*: Numeric field<br>*Variable name*: Lac_7<br>*Field required*: Required<br>*Field min*: 0<br>*Field max*: 25<br>*Measurement Unit*: mmol/L	mmol/L
15.2.1.2.17	***If 'Type of respiratory support' is equal to 'Mechanical ventilation' answer this question:***<br>Arterial saturation<br>*Notice shown if field's value is smaller than 80: 'Low value! Please control data input or make a comment.'*<br>*Field type*: Numeric field<br>*Variable name*: Sat_7<br>*Field required*: Required<br>*Field min*: 60<br>*Field max*: 100<br>*Measurement Unit*: %	%
15.2.1.2.7.3	***If 'Unit used for EtCO2, PaO2 and PaCO2' is equal to 'kPa' answer this question:***<br>Arterial PaO2<br>*Warning shown if field's value is smaller than 7: 'Low value! Please control data input or make a comment.'*<br>*Warning shown if field's value is larger than 18: 'High value! Please control data input or make a comment.'*<br>*Field type*: Numeric field<br>*Variable name*: PaO2_kpa_7<br>*Field required*: Required<br>*Field min*: 4<br>*Field max*: 60<br>*Measurement Unit*: kPa	kPa

15.2.1.2.7.4	***If 'Unit used for EtCO2, PaO2 and PaCO2' is equal to 'mmHg' answer this question:***<br>Arterial PaO2<br>*Warning shown if field's value is smaller than 50: 'Low value! Please control data input or make a comment.'*<br>*Warning shown if field's value is larger than 150: 'High value! Please control data input or make a comment.'*<br>*Field type*: Numeric field<br>*Variable name*: PaO2_mmhg_7<br>*Field required*: Required<br>*Field min*: 28<br>*Field max*: 500<br>*Measurement Unit*: mmHg	[ ] mmHg
15.2.1.2.7.5	***If 'Unit used for EtCO2, PaO2 and PaCO2' is equal to 'kPa' answer this question:***<br>Arterial PaCO2<br>*Warning shown if field's value is smaller than 2: 'Low value! Please control data input or make a comment.'*<br>*Warning shown if field's value is larger than 8: 'High value! Please control data input or make a comment.'*<br>*Field type*: Numeric field<br>*Variable name*: PaCO2_kpa_7<br>*Field required*: Required<br>*Field min*: 1<br>*Field max*: 20<br>*Measurement Unit*: kPa	[ ] kPa
15.2.1.2.7.6	***If 'Unit used for EtCO2, PaO2 and PaCO2' is equal to 'mmHg' answer this question:***<br>Arterial PaCO2<br>*Warning shown if field's value is smaller than 15: 'Low value! Please control data input or make a comment.'*<br>*Warning shown if field's value is larger than 59: 'High value! Please control data input or make a comment.'*<br>*Field type*: Numeric field<br>*Variable name*: PaCO2_mmhg_7<br>*Field required*: Required<br>*Field min*: 7<br>*Field max*: 140<br>*Measurement Unit*: mmHg	[ ] mmHg

16. Follow-up - Day 14

Number	Question	Answers
16.1	Fill in on date<br>*Field type*: Calculation<br>*Variable name*: date_14<br>*Field required*: Not required	*Template:* var randomization= moment('{castorRandomizedDateAndTime}', 'DD-MM-YYYY'); var newDate = randomization.add(14, 'days'); newDate.format('DD-MM-YYYY');

16.2	Status patient<br>*Notice shown if field's value is equal to Deceased: 'GO TO DAY 90'*<br>At 6:00 AM<br><br>*Field type*: Radiobutton<br>*Variable name*: Life_status_14<br>*Field required*: Required<br>*Option group name*: Alive/deceased	◯ Alive<br>◯ Deceased
16.2.1	***If 'Status patient' is equal to 'Alive' answer this question:***<br>Is the patient hospitalized?<br>*Notice shown if field's value is equal to No: 'FOLLOW UP DAY 14 COMPLETE.'*<br>At 6:00 AM<br><br>*Field type*: Radiobutton<br>*Variable name*: Hospital_patient_14<br>*Field required*: Required<br>*Option group name*: Yes/No	◯ Yes<br>◯ No
16.2.1.1	***If 'Is the patient hospitalized?' is equal to 'Yes' answer this question:***<br>Location of the patient<br>At 6:00 AM<br><br>*Field type*: Radiobutton<br>*Variable name*: Loc_ICUhosp_14<br>*Field required*: Required<br>*Option group name*: ICU/hospital	◯ Intensive Care Unit<br>◯ Hospital ward
16.2.1.1.1	***If 'Location of the patient' is equal to 'Hospital ward' answer this question:***<br>Type of respiratory support<br>At 6:00 AM<br><br>*Field type*: Radiobutton<br>*Variable name*: Resp_hospsupp_14<br>*Field required*: Required<br>*Option group name*: resp_supp_hosp	◯ No oxygen therapy<br>◯ Oxygen by mask or nasal prongs<br>◯ High Flow Nasal Oxygen (HFNO) therapy<br>◯ Non-invasive ventilation (NIV)
16.2.1.1.2	***If 'Location of the patient' is equal to 'Intensive Care Unit' answer this question:***<br>Type of respiratory support<br>At 6:00 AM<br><br>*Field type*: Radiobutton<br>*Variable name*: Resp_supp_14<br>*Field required*: Required<br>*Option group name*: Respiratory support	◯ No oxygen therapy<br>◯ Oxygen by maks or nasal prongs<br>◯ High Flow Nasal Oxygen (HFNO) therapy<br>◯ Non-invasive ventilation (NIV)<br>◯ Mechanical ventilation

16.2.1.1.2.1	***If 'Type of respiratory support' is equal to 'Mechanical ventilation' answer this question:***<br>PaO2/FiO2 ratio<br>At 6:00 AM<br><br>*Field type*: Radiobutton<br>*Variable name*: PF_14<br>*Field required*: Required<br>*Option group name*: PF ratio	◯ ≥ 150<br>◯ < 150<br>◯ < 150 and vasopressors, dialysis or ECMO
	**EVENTS**	
16.3	***If 'Is the patient hospitalized during any time of the day?' is equal to 'Yes' answer this question:***<br>Did an event occur?<br>Only report events if they occurred from day 8 until day 14 when the patient was admitted on the ICU.<br><br>• Renal Replacement Therapy during any time of the day.<br>• Placement of a tracheostomy during any time of the day<br>• Use of inhaled vasodilators during any time of the day<br>• Use of airway pressure release ventlation during any time of the day<br>• Use of ECMO during any time of the day<br><br>*Field type*: Checkbox<br>*Variable name*: Event_14<br>*Field required*: Required<br>*Option group name*: Events	☐ None<br>☐ Use of renal replacement therapy<br>☐ Placement of a tracheostomy<br>☐ Use of inhaled vasodilators<br>☐ Use of airway pressure release ventilation<br>☐ Use of ECMO<br>☐ Continuous infusion of neuromuscular blocking agents
16.4	***If 'Is the patient hospitalized during any time of the day?' is equal to 'Yes' answer this question:***<br>Calculation for incorrect answer event<br>*Field type*: Calculation<br>*Variable name*: Event_calc_incorrect_14<br>*Field required*: Not required	*Template:* '##allowempty##' var splitted = "{Event_14}".split(';'); if (splitted.indexOf("0") > -1 && (splitted.indexOf("1") > -1 || splitted.indexOf("2") > -1 || splitted.indexOf("3") > -1 || splitted.indexOf("4") > -1 || splitted.indexOf("5") > -1 || splitted.indexOf("6") > -1)) { '1'; } else { '0'; };
	Combination **NOT** possible! **NONE** cannot be chosen when other options are selected!	
	**COMPLICATIONS**	

16.5	***If 'Is the patient hospitalized during any time of the day?' is equal to 'Yes' answer this question:***<br>Did a complication occur?<br>Only report complications if they occurred from day 8 until day 14 when the patient was admitted on the ICU.<br>• Pneumothorax: Air in the pleural cavity developed after randomization confirmed by a radiologist on a CT-thorax or chest radiograph for which a drain has been placed.<br>• Ventilator Associated Pneumonia: Clinical Pulmonary Infection Score (CPIS) > 5 with an infiltration on CXR and developed after intubation.<br>*Field type*: Checkbox<br>*Variable name*: Compl_14<br>*Field required*: Required<br>*Option group name*: Complications	☐ None<br>☐ Diagnosis of pneumothorax<br>☐ Diagnosis of Ventilator Associated Pneumonia
16.6	***If 'Is the patient hospitalized during any time of the day?' is equal to 'Yes' answer this question:***<br>Calculation for incorrect answer complications<br>*Field type*: Calculation<br>*Variable name*: Compl_calc_incorrect_14<br>*Field required*: Not required	*Template:* '##allowempty##' var splitted = "{Compl_14}".split(';'); if (splitted.indexOf("0") > -1 && (splitted.indexOf("1") > -1 || splitted.indexOf("2") > -1 || splitted.indexOf("3") > -1 || splitted.indexOf("4") > -1 || splitted.indexOf("5") > -1 || splitted.indexOf("6") > -1)) { '1'; } else { '0'; };

Combination **NOT** possible! **NONE** cannot be chosen when other options are selected!

17. Follow-up - Day 21

Number	Question	Answers
17.1	Fill in on date<br>*Field type*: Calculation<br>*Variable name*: date_21<br>*Field required*: Not required	*Template:* var randomization= moment('{castorRandomizedDateAndTime}', 'DD-MM-YYYY'); var newDate = randomization.add(21, 'days'); newDate.format('DD-MM-YYYY');
17.2	***If 'Status patient' is equal to 'Alive' answer this question:***<br>Status patient<br>*Notice shown if field's value is equal to Deceased: 'GO TO DAY 90'*<br>At 6:00 AM<br>*Field type*: Radiobutton<br>*Variable name*: Life_status_21<br>*Field required*: Required<br>*Option group name*: Alive/deceased	○ Alive<br>○ Deceased

17.2.1	***If 'Status patient' is equal to 'Alive' answer this question:***<br>Is the patient hospitalized?<br>*Notice shown if field's value is equal to No: 'FOLLOW UP DAY 21 COMPLETE.'*<br>At 6:00 AM<br><br>*Field type*: Radiobutton<br>*Variable name*: Hospital_patient_21<br>*Field required*: Required<br>*Option group name*: Yes/No	◯ Yes<br>◯ No
17.2.1.1	***If 'Is the patient hospitalized?' is equal to 'Yes' answer this question:***<br>Location of the patient<br>At 6:00 AM<br><br>*Field type*: Radiobutton<br>*Variable name*: Loc_ICUhosp_21<br>*Field required*: Required<br>*Option group name*: ICU/hospital	◯ Intensive Care Unit<br>◯ Hospital ward
17.2.1.1.1	***If 'Location of the patient' is equal to 'Hospital ward' answer this question:***<br>Type of respiratory support<br>At 6:00 AM<br><br>*Field type*: Radiobutton<br>*Variable name*: Resp_hospsupp_21<br>*Field required*: Required<br>*Option group name*: resp_supp_hosp	◯ No oxygen therapy<br>◯ Oxygen by mask or nasal prongs<br>◯ High Flow Nasal Oxygen (HFNO) therapy<br>◯ Non-invasive ventilation (NIV)
17.2.1.1.2	***If 'Location of the patient' is equal to 'Intensive Care Unit' answer this question:***<br>Type of respiratory support<br>At 6:00 AM<br><br>*Field type*: Radiobutton<br>*Variable name*: Resp_supp_21<br>*Field required*: Required<br>*Option group name*: Respiratory support	◯ No oxygen therapy<br>◯ Oxygen by maks or nasal prongs<br>◯ High Flow Nasal Oxygen (HFNO) therapy<br>◯ Non-invasive ventilation (NIV)<br>◯ Mechanical ventilation
17.2.1.1.2.1	***If 'Type of respiratory support' is equal to 'Mechanical ventilation' answer this question:***<br>PaO2/FiO2 ratio<br>At 6:00 AM<br><br>*Field type*: Radiobutton<br>*Variable name*: PF_21<br>*Field required*: Required<br>*Option group name*: PF ratio	◯ ≥ 150<br>◯ < 150<br>◯ < 150 and vasopressors, dialysis or ECMO
	**EVENTS**	

17.3	***If 'Is the patient hospitalized?' is equal to 'Yes' answer this question:***<br>Did an event occur?<br>Only report events if they occurred from day 15 until day 21 when the patient was admitted on the ICU.<br>• Renal Replacement Therapy during any time of the day.<br>• Placement of a tracheostomy during any time of the day<br>• Use of inhaled vasodilators during any time of the day<br>• Use of airway pressure release ventlation during any time of the day<br>• Use of ECMO during any time of the day<br><br>*Field type*: Checkbox<br>*Variable name*: Event_21<br>*Field required*: Required<br>*Option group name*: Events	☐ None<br>☐ Use of renal replacement therapy<br>☐ Placement of a tracheostomy<br>☐ Use of inhaled vasodilators<br>☐ Use of airway pressure release ventilation<br>☐ Use of ECMO<br>☐ Continuous infusion of neuromuscular blocking agents
17.4	***If 'Is the patient hospitalized?' is equal to 'Yes' answer this question:***<br>Calculation for incorrect answer event<br>*Field type*: Calculation<br>*Variable name*: Event_calc_incorrect_21<br>*Field required*: Not required	*Template:* '##allowempty##' var splitted = "{Event_21}".split(';'); if (splitted.indexOf("0") > -1 && (splitted.indexOf("1") > -1 || splitted.indexOf("2") > -1 || splitted.indexOf("3") > -1 || splitted.indexOf("4") > -1 || splitted.indexOf("5") > -1 || splitted.indexOf("6") > -1)) { '1'; } else { '0'; };
	Combination **NOT** possible! **NONE** cannot be chosen when other options are selected!	
	**COMPLICATIONS**	
17.5	***If 'Is the patient hospitalized?' is equal to 'Yes' answer this question:***<br>Did a complication occur?<br>Only report complications if they occurred from day 15 until day 21 when the patient was admitted on the ICU.<br>• Pneumothorax: Air in the pleural cavity developed after randomization confirmed by a radiologist on a CT-thorax or chest radiograph for which a drain has been placed.<br>• Ventilator Associated Pneumonia: Clinical Pulmonary Infection Score (CPIS) > 5 with an infiltration on CXR and developed after intubation.<br><br>*Field type*: Checkbox<br>*Variable name*: Compl_21<br>*Field required*: Required<br>*Option group name*: Complications	☐ None<br>☐ Diagnosis of pneumothorax<br>☐ Diagnosis of Ventilator Associated Pneumonia

17.6	***If 'Is the patient hospitalized?' is equal to 'Yes' answer this question:***<br>Calculation for incorrect answer complications<br>*Field type*: Calculation<br>*Variable name*: Compl_calc_incorrect_21<br>*Field required*: Not required	*Template:* '##allowempty##' var splitted = "{Compl_21}".split(';'); if (splitted.indexOf("0") > -1 && (splitted.indexOf("1") > -1 || splitted.indexOf("2") > -1 || splitted.indexOf("3") > -1 || splitted.indexOf("4") > -1 || splitted.indexOf("5") > -1 || splitted.indexOf("6") > -1)) { '1'; } else { '0'; };
	Combination **NOT** possible! **NONE** cannot be chosen when other options are selected!	

18. Follow-up - Day 28

Number	Question	Answers
18.1	Fill in on date<br>*Field type*: Calculation<br>*Variable name*: date_28<br>*Field required*: Not required	*Template:* var randomization= moment('{castorRandomizedDateAndTime}', 'DD-MM-YYYY'); var newDate = randomization.add(28, 'days'); newDate.format('DD-MM-YYYY');
18.2	***If 'Status patient' is equal to 'Alive' answer this question:***<br>Status patient<br>*Notice shown if field's value is equal to Deceased: 'GO TO DAY 90'*<br>At 6:00 AM<br><br>*Field type*: Radiobutton<br>*Variable name*: Life_status_28<br>*Field required*: Required<br>*Option group name*: Alive/deceased	◯ Alive<br>◯ Deceased
18.2.1	***If 'Status patient' is equal to 'Alive' answer this question:***<br>Is the patient hospitalized?<br>At 6:00 AM<br><br>*Field type*: Radiobutton<br>*Variable name*: Hospital_patient_28<br>*Field required*: Required<br>*Option group name*: Yes/No	◯ Yes<br>◯ No

18.2.1.1	***If 'Is the patient hospitalized?' is equal to 'No' answer this question:***<br>Date of latest successfull extubation from the ventilator before day 28.<br>A patiënt is successfull extubated from the ventilator if he/she is >24 hours released from the ventilator within 28 days after randomisation.<br><br>Example patient with day 28 on 10-feb.<br><br>Example 1: If the patient was extubated on 20-jan and not re-intubated, you need to fill in 20-jan as the successful extubation date.<br><br>Example 2: If the patient was extubated on 20-jan however reintubated from 1-feb until 5-feb, you need to fill in 5-feb as successful extubation date.<br><br>Example 3: If the patient was extubated on 20-jan however reintubated from 11-feb until 15-feb, you need to fill in 20-jan as successful extubation date.<br><br>*Field type*: Date<br>*Variable name*: Date_VFD<br>*Field required*: Required	☐ ☐ ☐ *(dd-mm-yyyy)*
	DATE OF EXTUBATION IS AFTER DAY 28, PLEASE CHECK INPUT	
	DATE OF EXTUBATION IS BEFORE RANDOMIZATION DATE, PLEASE CHECK INPUT	
18.2.1.2	***If 'Is the patient hospitalized?' is equal to 'Yes' answer this question:***<br>Location of the patient<br>At 6:00 AM<br><br>*Field type*: Radiobutton<br>*Variable name*: Loc_ICUhosp_28<br>*Field required*: Required<br>*Option group name*: ICU/hospital	◯ Intensive Care Unit<br>◯ Hospital ward

18.2.1.2.1	***If 'Location of the patient' is equal to 'Hospital ward' answer this question:***<br>Date of latest successfull extubation from the ventilator before day 28.<br>A patiënt is successfull extubated from the ventilator if he/she is >24 hours released from the ventilator within 28 days after randomisation.<br><br>Example patient with day 28 on 10-feb.<br><br>Example 1: If the patient was extubated on 20-jan and not re-intubated, you need to fill in 20-jan as the successful extubation date.<br><br>Example 2: If the patient was extubated on 20-jan however reintubated from 1-feb until 5-feb, you need to fill in 5-feb as successful extubation date.<br><br>Example 3: If the patient was extubated on 20-jan however reintubated from 11-feb until 15-feb, you need to fill in 20-jan as successful extubation date.<br><br>*Field type*: Date<br>*Variable name*: Date_VFD_1<br>*Field required*: Required	☐ ☐ ☐ *(dd-mm-yyyy)*
	DATE OF EXTUBATION IS AFTER DAY 28, PLEASE CHECK INPUT	
	DATE OF EXTUBATION IS BEFORE RANDOMIZATION DATE, PLEASE CHECK INPUT	
18.2.1.2.2	***If 'Location of the patient' is equal to 'Hospital ward' answer this question:***<br>Type of respiratory support<br>At 6:00 AM<br><br>*Field type*: Radiobutton<br>*Variable name*: Resp_hospsupp_28<br>*Field required*: Required<br>*Option group name*: resp_supp_hosp	○ No oxygen therapy<br>○ Oxygen by mask or nasal prongs<br>○ High Flow Nasal Oxygen (HFNO) therapy<br>○ Non-invasive ventilation (NIV)
18.2.1.2.3	***If 'Location of the patient' is equal to 'Intensive Care Unit' answer this question:***<br>Type of respiratory support<br>At 6:00 AM<br><br>*Field type*: Radiobutton<br>*Variable name*: Resp_supp_28<br>*Field required*: Required<br>*Option group name*: Respiratory support	○ No oxygen therapy<br>○ Oxygen by maks or nasal prongs<br>○ High Flow Nasal Oxygen (HFNO) therapy<br>○ Non-invasive ventilation (NIV)<br>○ Mechanical ventilation
18.2.1.2.3.1	***If 'Type of respiratory support' is equal to 'Mechanical ventilation' answer this question:***<br>PaO2/FiO2 ratio<br>At 6:00 AM<br><br>*Field type*: Radiobutton<br>*Variable name*: PF_28<br>*Field required*: Required<br>*Option group name*: PF ratio	○ ≥ 150<br>○ < 150<br>○ < 150 and vasopressors, dialysis or ECMO

18.2.1.2.3.2	***If 'Type of respiratory support' is not equal to 'Mechanical ventilation' answer this question:***<br>Date of latest successfull extubation from the ventilator before day 28.<br>A patiënt is successfull extubated from the ventilator if he/she is >24 hours released from the ventilator within 28 days after randomisation.<br><br>Example patient with day 28 on 10-feb.<br><br>Example 1: If the patient was extubated on 20-jan and not re-intubated, you need to fill in 20-jan as the successful extubation date.<br><br>Example 2: If the patient was extubated on 20-jan however reintubated from 1-feb until 5-feb, you need to fill in 5-feb as successful extubation date.<br><br>Example 3: If the patient was extubated on 20-jan however reintubated from 11-feb until 15-feb, you need to fill in 20-jan as successful extubation date.<br><br>*Field type*: Date<br>*Variable name*: Date_VFD_2<br>*Field required*: Required	☐ ☐ ☐ *(dd-mm-yyyy)*
	DATE OF EXTUBATION IS AFTER DAY 28, PLEASE CHECK INPUT	
	DATE OF EXTUBATION IS BEFORE RANDOMIZATION DATE, PLEASE CHECK INPUT	
	**EVENTS**	
18.3	***If 'Is the patient hospitalized?' is equal to 'Yes' answer this question:***<br>Did an event occur?<br>Only report events if they occurred from day 22 until day 28 when the patient was admitted on the ICU.<br>• Renal Replacement Therapy during any time of the day.<br>• Placement of a tracheostomy during any time of the day<br>• Use of inhaled vasodilators during any time of the day<br>• Use of airway pressure release ventlation during any time of the day<br>• Use of ECMO during any time of the day<br><br>*Field type*: Checkbox<br>*Variable name*: Event_28<br>*Field required*: Required<br>*Option group name*: Events	☐ None<br>☐ Use of renal replacement therapy<br>☐ Placement of a tracheostomy<br>☐ Use of inhaled vasodilators<br>☐ Use of airway pressure release ventilation<br>☐ Use of ECMO<br>☐ Continuous infusion of neuromuscular blocking agents
18.4	***If 'Is the patient hospitalized?' is equal to 'Yes' answer this question:***<br>Calculation for incorrect answer event<br>*Field type*: Calculation<br>*Variable name*: Event_calc_incorrect_28<br>*Field required*: Not required	*Template:* '##allowempty##' var splitted = "{Event_28}".split(';'); if (splitted.indexOf("0") > -1 && (splitted.indexOf("1") > -1 || splitted.indexOf("2") > -1 || splitted.indexOf("3") > -1 || splitted.indexOf("4") > -1 || splitted.indexOf("5") > -1 || splitted.indexOf("6") > -1)) { '1'; } else { '0'; };

	Combination **NOT** possible! **NONE** cannot be chosen when other options are selected!	
	**COMPLICATIONS**	
18.5	***If 'Is the patient hospitalized?' is equal to 'Yes' answer this question:***<br>Did a complication occur?<br>Only report complications if they occurred from day 22 until day 28 when the patient was admitted on the ICU.<br>• Pneumothorax: Air in the pleural cavity developed after randomization confirmed by a radiologist on a CT-thorax or chest radiograph for which a drain has been placed.<br>• Ventilator Associated Pneumonia: Clinical Pulmonary Infection Score (CPIS) > 5 with an infiltration on CXR and developed after intubation.<br>*Field type*: Checkbox<br>*Variable name*: Compl_28<br>*Field required*: Required<br>*Option group name*: Complications	☐ None<br>☐ Diagnosis of pneumothorax<br>☐ Diagnosis of Ventilator Associated Pneumonia
18.6	***If 'Is the patient hospitalized?' is equal to 'Yes' answer this question:***<br>Calculation for incorrect answer complications<br>*Field type*: Calculation<br>*Variable name*: Compl_calc_incorrect_28<br>*Field required*: Not required	*Template:* '##allowempty##' var splitted = "{Compl_28}".split(';'); if (splitted.indexOf("0") > -1 && (splitted.indexOf("1") > -1 || splitted.indexOf("2") > -1 || splitted.indexOf("3") > -1 || splitted.indexOf("4") > -1 || splitted.indexOf("5") > -1 || splitted.indexOf("6") > -1)) { '1'; } else { '0'; };
	Combination **NOT** possible! **NONE** cannot be chosen when other options are selected!	
18.7	Calculation VFD vs day 28<br>*Field type*: Calculation<br>*Variable name*: Cal_VFD_date_1<br>*Field required*: Not required	*Template:* var start = moment('{date_28}', 'DD-MM-YYYY'); var now = moment('{Date_VFD}', 'DD-MM-YYYY'); now.diff(start, 'days');
18.8	Calculation ICUdis vs randomization date<br>*Warning shown if field's value is smaller than 0: 'DATE OF DEATH IS BEFORE RANDOMIZATION DATE, PLEASE CONTROL INPUT'*<br>*Warning shown if field's value is larger than 90: 'DATE OF DEATH IS AFTER DAY 90, PLEASE CONTROL INPUT'*<br>*Field type*: Calculation<br>*Variable name*: Cal_VFD_date_2<br>*Field required*: Not required	*Template:* var start = moment('{castorRandomizedDateAndTime}', 'DD-MM-YYYY'); var now = moment('{Date_VFD}', 'DD-MM-YYYY'); now.diff(start, 'days');
18.9	Calculation VFD vs day 28<br>*Field type*: Calculation<br>*Variable name*: Cal_VFD_1_date_1<br>*Field required*: Not required	*Template:* var start = moment('{date_28}', 'DD-MM-YYYY'); var now = moment('{Date_VFD_1}', 'DD-MM-YYYY'); now.diff(start, 'days');

18.10	Calculation ICUdis vs randomization date<br>*Warning shown if field's value is smaller than 0: 'DATE OF DEATH IS BEFORE RANDOMIZATION DATE, PLEASE CONTROL INPUT'*<br>*Warning shown if field's value is larger than 90: 'DATE OF DEATH IS AFTER DAY 90, PLEASE CONTROL INPUT'*<br>*Field type*: Calculation<br>*Variable name*: Cal_VFD_1_date_2<br>*Field required*: Not required	*Template:* var start = moment('{castorRandomizedDateAndTime}', 'DD-MM-YYYY'); var now = moment('{Date_VFD_1}', 'DD-MM-YYYY'); now.diff(start, 'days');
18.11	Calculation VFD vs day 28<br>*Field type*: Calculation<br>*Variable name*: Cal_VFD_2_date_1<br>*Field required*: Not required	*Template:* var start = moment('{date_28}', 'DD-MM-YYYY'); var now = moment('{Date_VFD_2}', 'DD-MM-YYYY'); now.diff(start, 'days');
18.12	Calculation ICUdis vs randomization date<br>*Warning shown if field's value is smaller than 0: 'DATE OF DEATH IS BEFORE RANDOMIZATION DATE, PLEASE CONTROL INPUT'*<br>*Warning shown if field's value is larger than 90: 'DATE OF DEATH IS AFTER DAY 90, PLEASE CONTROL INPUT'*<br>*Field type*: Calculation<br>*Variable name*: Cal_VFD_2_date_2<br>*Field required*: Not required	*Template:* var start = moment('{castorRandomizedDateAndTime}', 'DD-MM-YYYY'); var now = moment('{Date_VFD_2}', 'DD-MM-YYYY'); now.diff(start, 'days');

19. Follow-up - Day 90

Number	Question	Answers
19.1	Fill in on date<br>*Field type*: Calculation<br>*Variable name*: date_90<br>*Field required*: Not required	*Template:* var randomization= moment('{castorRandomizedDateAndTime}', 'DD-MM-YYYY'); var newDate = randomization.add(90, 'days'); newDate.format('DD-MM-YYYY');
19.2	Status patient<br>At 6:00 AM<br>*Field type*: Radiobutton<br>*Variable name*: Life_status_90<br>*Field required*: Required<br>*Option group name*: Alive/deceased	○ Alive<br>○ Deceased
19.2.1	***If 'Status patient' is equal to 'Deceased' answer this question:***<br>Date of death.<br>*Field type*: Date<br>*Variable name*: Death_date<br>*Field required*: Required	[ ] [ ] [ ] *(dd-mm-yyyy)*

19.2.2	***If 'Status patient' is equal to 'Deceased' answer this question:***<br>Place of death.<br>*Field type*: Radiobutton<br>*Variable name*: Death_location<br>*Field required*: Required<br>*Option group name*: Location death	◯ ICU<br>◯ Hospital ward<br>◯ Outside the hospital
19.2.3	***If 'Status patient' is equal to 'Alive' answer this question:***<br>Is the patient discharged from the ICU?<br>At 6:00 AM<br><br>*Field type*: Radiobutton<br>*Variable name*: Dis_ICU<br>*Field required*: Required<br>*Option group name*: Yes/No	◯ Yes<br>◯ No
19.2.3.1	***If 'Is the patient discharged from the ICU?' is equal to 'Yes' answer this question:***<br>Date of latest successfull discharge from the ICU before day 90.<br>Latest successfull discharge (>24 hours) from the ICU within 90 days after randomisation.<br><br>Example patient with day 90 on 5-april.<br><br>Example 1: If the patient was discharged on 5-feb and not readmitted, you need to fill in 5-feb as the successful discharge date.<br><br>Example 2: If the patient was discharged on 26-jan however readmitted from 1-feb until 21-feb, you need to fill in 21-feb as successful discharge date.<br><br>Example 3: If the patient was discharged on 26-jan however readmitted from 10-april until 21-april, you need to fill in 26-jan as successful discharge date.<br><br>*Field type*: Date<br>*Variable name*: Date_ICUdis<br>*Field required*: Required	[ ] [ ] [ ] *(dd-mm-yyyy)*
	DATE OF ICU DISCHARGE IS BEFORE RANDOMIZATION DATE, PLEASE CHECK INPUT	
	DATE OF ICU DISCHARGE IS AFTER DAY 90, PLEASE CHECK INPUT	

19.3	Days on ICU after randomization until day 90.<br>Count every day that the patient has been in the intensive care unit from randomization until day 90. Don't forget to count the days of readmission. Round the number to whole days.<br><br>Examples with randomization on 10-jan and day 90 on 10-apr.<br><br>Example 1: If the patient was discharged on 5-feb and not readmitted, you need to fill in 27 days.<br><br>Example 2: If the patient was discharged on 26-jan however readmitted from 1-feb until 21-feb, you need to fill in 38 days.<br><br>Example 3: If the patient was discharged on 26-jan however readmitted from 1-apr until 21-apr, you need to fill in 27 days. Days after day 90 does not count.<br><br>*Field type*: Numeric field<br>*Variable name*: ICU_days<br>*Field required*: Required<br>*Field min*: 1<br>*Field max*: 90	
19.2.3.2	***If 'Is the patient discharged from the ICU?' is equal to 'No' answer this question:***<br>Type of respiratory support<br>At 6:00 AM<br><br>*Field type*: Radiobutton<br>*Variable name*: Resp_supp_90<br>*Field required*: Required<br>*Option group name*: Respiratory support	○ No oxygen therapy<br>○ Oxygen by maks or nasal prongs<br>○ High Flow Nasal Oxygen (HFNO) therapy<br>○ Non-invasive ventilation (NIV)<br>○ Mechanical ventilation
19.2.3.2.1	***If 'Type of respiratory support' is equal to 'Mechanical ventilation' answer this question:***<br>PaO2/FiO2 ratio<br>At 6:00 AM<br><br>*Field type*: Radiobutton<br>*Variable name*: PF_90<br>*Field required*: Required<br>*Option group name*: PF ratio	○ ≥ 150<br>○ < 150<br>○ < 150 and vasopressors, dialysis or ECMO
19.2.3.3	***If 'Is the patient discharged from the ICU?' is equal to 'Yes' answer this question:***<br>Is the patient discharged from the hospital?<br>At 6:00 AM<br><br>*Field type*: Radiobutton<br>*Variable name*: Dis_hosp<br>*Field required*: Required<br>*Option group name*: Yes/No	○ Yes<br>○ No

19.2.3.3.1	***If 'Is the patient discharged from the hospital?' is equal to 'Yes' answer this question:***<br>Date of hospital discharge.<br>Fill in the date of discharge from the hospital within 90 days after randomisation.<br><br>*Field type*: Date<br>*Variable name*: Date_hospdis<br>*Field required*: Required	*(dd-mm-yyyy)*
	DATE OF HOSPITAL DISCHARGE IS AFTER DAY 90, PLEASE CHECK INPUT	
	DATE OF HOSPITAL DISCHARGE IS BEFORE ICU DISCHARGE, PLEASE CHECK INPUT	
19.2.3.3.2	***If 'Is the patient discharged from the hospital?' is equal to 'No' answer this question:***<br>Type of respiratory support<br>At 6:00 AM<br><br>*Field type*: Radiobutton<br>*Variable name*: Resp_hospsupp_90<br>*Field required*: Required<br>*Option group name*: resp_supp_hosp	◯ No oxygen therapy<br>◯ Oxygen by mask or nasal prongs<br>◯ High Flow Nasal Oxygen (HFNO) therapy<br>◯ Non-invasive ventilation (NIV)
19.4	Days on ventilator after randomization until day 90.<br>Count every day that the patient received invasive ventilation from randomization to successful extubation. When a patient is not extubated during the study period, please fill in 90 days.<br><br>Examples with randomization on 10-jan and day 90 on 10-apr.<br><br>Example 1: If the patient was extubated on 20-jan and not re-intubated, you need to fill in 11 days.<br><br>Example 2: If the patient was extubated on 20-jan however reintubated from 1-feb until 5-feb, you need to fill in 16 days.<br><br>Example 3: If the patient was extubated on 20-jan however reintubated from 5-apr until 20-apr, you need to fill in 17 days. Days after day 90 does not count.<br><br>*Field type*: Numeric field<br>*Variable name*: Ven_days<br>*Field required*: Required<br>*Field min*: 0<br>*Field max*: 90	
	**EVENTS**	

19.5	***If 'Is the patient hospitalized?' is equal to 'Yes' answer this question:***<br>Did an event occur?<br>Only report events if they occurred from day 29 until day 90 when the patient was admitted on the ICU.<br>• Renal Replacement Therapy during any time of the day.<br>• Placement of a tracheostomy during any time of the day<br>• Use of inhaled vasodilators during any time of the day<br>• Use of airway pressure release ventlation during any time of the day<br>• Use of ECMO during any time of the day<br>*Field type*: Checkbox<br>*Variable name*: Event_90<br>*Field required*: Required<br>*Option group name*: Events	☐ None<br>☐ Use of renal replacement therapy<br>☐ Placement of a tracheostomy<br>☐ Use of inhaled vasodilators<br>☐ Use of airway pressure release ventilation<br>☐ Use of ECMO<br>☐ Continuous infusion of neuromuscular blocking agents
19.6	***If 'Is the patient hospitalized?' is equal to 'Yes' answer this question:***<br>Calculation for incorrect answer event<br>*Field type*: Calculation<br>*Variable name*: Event_calc_incorrect_90<br>*Field required*: Not required	*Template:* '##allowempty##' var splitted = "{Event_90}".split(';'); if (splitted.indexOf("0") > -1 && (splitted.indexOf("1") > -1 || splitted.indexOf("2") > -1 || splitted.indexOf("3") > -1 || splitted.indexOf("4") > -1 || splitted.indexOf("5") > -1 || splitted.indexOf("6") > -1)) { '1'; } else { '0'; };
	Combination **NOT** possible! **NONE** cannot be chosen when other options are selected!	
	**COMPLICATIONS**	
19.7	***If 'Is the patient hospitalized?' is equal to 'Yes' answer this question:***<br>Did a complication occur?<br>Only report complications if they occurred from day 29 until day 90 when the patient was admitted on the ICU.<br>• Pneumothorax: Air in the pleural cavity developed after randomization confirmed by a radiologist on a CT-thorax or chest radiograph for which a drain has been placed.<br>• Ventilator Associated Pneumonia: Clinical Pulmonary Infection Score (CPIS) > 5 with an infiltration on CXR and developed after intubation.<br>*Field type*: Checkbox<br>*Variable name*: Compl_90<br>*Field required*: Required<br>*Option group name*: Complications	☐ None<br>☐ Diagnosis of pneumothorax<br>☐ Diagnosis of Ventilator Associated Pneumonia
19.8	***If 'Is the patient hospitalized?' is equal to 'Yes' answer this question:***<br>Calculation for incorrect answer complications<br>*Field type*: Calculation<br>*Variable name*: Compl_calc_incorrect_90<br>*Field required*: Not required	*Template:* '##allowempty##' var splitted = "{Compl_90}".split(';'); if (splitted.indexOf("0") > -1 && (splitted.indexOf("1") > -1 || splitted.indexOf("2") > -1 || splitted.indexOf("3") > -1 || splitted.indexOf("4") > -1 || splitted.indexOf("5") > -1 || splitted.indexOf("6") > -1)) { '1'; } else { '0'; };
	Combination **NOT** possible! **NONE** cannot be chosen when other options are selected!	

CLINICAL FRAILTY SCALE

19.9	Calculation death vs randomization date<br>*Warning shown if field's value is smaller than 0: 'DATE OF DEATH IS BEFORE RANDOMIZATION DATE, PLEASE CONTROL INPUT'*<br>*Warning shown if field's value is larger than 90: 'DATE OF DEATH IS AFTER DAY 90, PLEASE CONTROL INPUT'*<br>*Field type*: Calculation<br>*Variable name*: Cal_death_date_2<br>*Field required*: Not required	*Template:* var start = moment('{castorRandomizedDateAndTime}', 'DD-MM-YYYY'); var now = moment('{Death_date}', 'DD-MM-YYYY'); now.diff(start, 'days');
	DATE OF DEATH IS BEFORE RANDOMIZATION DATE, PLEASE CHECK INPUT	
19.10	Calculation death vs day 90<br>*Field type*: Calculation<br>*Variable name*: Cal_death_date_1<br>*Field required*: Not required	*Template:* var start = moment('{date_90}', 'DD-MM-YYYY'); var now = moment('{Death_date}', 'DD-MM-YYYY'); now.diff(start, 'days');
	DATE OF DEATH IS AFTER DAY 90, PLEASE CHECK INPUT	
19.2.5	***If 'Status patient' is equal to 'Alive' answer this question:***<br>Clinical Frailty Scale.<br>You can find extra information in the figure below.<br>*Field type*: Radiobutton<br>*Variable name*: Frailty_90<br>*Field required*: Required<br>*Option group name*: Fraily score	○ Very fit<br>○ Well<br>○ Managing Well<br>○ Vulnerable<br>○ Midly Frail<br>○ Moderately Frail<br>○ Severely Frail<br>○ Very Severely Frail<br>○ Terminally ill
19.2.6	***If 'Status patient' is equal to 'Alive' answer this question:***<br>Clinical Frailty Scale.<br>*Field type*: Image<br>*Variable name*: image_frailty_1<br>*Field required*: Not required	
19.11	Calculation ICUdis vs day 90<br>*Field type*: Calculation<br>*Variable name*: Cal_ICUdis_date_1<br>*Field required*: Not required	*Template:* var start = moment('{date_90}', 'DD-MM-YYYY'); var now = moment('{Date_ICUdis}', 'DD-MM-YYYY'); now.diff(start, 'days');
19.12	Calculation ICUdis vs randomization date<br>*Warning shown if field's value is smaller than 0: 'DATE OF DEATH IS BEFORE RANDOMIZATION DATE, PLEASE CONTROL INPUT'*<br>*Warning shown if field's value is larger than 90: 'DATE OF DEATH IS AFTER DAY 90, PLEASE CONTROL INPUT'*<br>*Field type*: Calculation<br>*Variable name*: Cal_ICUdis_date_2<br>*Field required*: Not required	*Template:* var start = moment('{castorRandomizedDateAndTime}', 'DD-MM-YYYY'); var now = moment('{Date_ICUdis}', 'DD-MM-YYYY'); now.diff(start, 'days');

19.13	Calculation Hospdis vs day 90<br>*Field type*: Calculation<br>*Variable name*: Cal_hospdis_date_1<br>*Field required*: Not required	*Template:* var start = moment('{date_90}', 'DD-MM-YYYY'); var now = moment('{Date_hospdis}', 'DD-MM-YYYY'); now.diff(start, 'days');
19.14	Calculation ICUdis vs Hospdis date<br>*Warning shown if field's value is smaller than 0: 'DATE OF DEATH IS BEFORE RANDOMIZATION DATE, PLEASE CONTROL INPUT'*<br>*Warning shown if field's value is larger than 90: 'DATE OF DEATH IS AFTER DAY 90, PLEASE CONTROL INPUT'*<br>*Field type*: Calculation<br>*Variable name*: Cal_hospdis_date_2<br>*Field required*: Not required	*Template:* var start = moment('{Date_ICUdis}', 'DD-MM-YYYY'); var now = moment('{Date_hospdis}', 'DD-MM-YYYY'); now.diff(start, 'days');

20. Protocol Deviations - Protocol Deviations

Number	Question	Answers
	If a protocol deviation occurred please fill in a report for every protocol deviation.	
20.1	Protocol Deviation<br>*Field type*: Add report button<br>*Variable name*: Button_PD<br>*Field required*: Not required	
